# Cryo-EM structure of the budding yeast telomerase holoenzyme

**DOI:** 10.1126/science.adz5344

**Published:** 2026-03-26

**Authors:** Hongmiao Hu, Hannah Neumann, Gabriela M. Teplitz, Elsa Franco-Echevarría, Pascal Chartrand, Raymund J. Wellinger, Thi Hoang Duong Nguyen

**Affiliations:** 1 MRC Laboratory of Molecular Biology, Cambridge CB2 0QH, UK; 2 Department of Biochemistry and Molecular Medicine, Institut Courtois d’Innovation Biomédicale, Université de Montréal, Quebec H3C 3J7, Canada; 3 Department of Microbiology and Infectious Diseases, University of Sherbrooke, Quebec J1E 4K8, Canada

## Abstract

Telomerase is a reverse transcriptase that synthesizes telomeric repeats at chromosome ends, safeguarding genome integrity. We present the cryo-electron microscopy structure of the budding yeast telomerase, which exhibits significant divergence from its ciliate and vertebrate counterparts. The structure reveals a stable core formed by telomerase RNA TLC1, the three Ever Shorter Telomere (Est) proteins, Est1, Est2 and Est3, and the Pop1/Pop6/Pop7 complex (Pop1/6/7). TLC1, Est3, and Pop1/6/7 serve critical roles in complex assembly. We identified a zinc finger (ZnF) motif in the telomerase reverse transcriptase (TERT) subunit Est2 that is crucial for telomerase function. Structure prediction suggests the presence of ZnFs in TERT from diverse species. These findings offer insights into the functional organization of yeast telomerase and underscore the evolutionary diversity of telomerase holoenzymes.

In most eukaryotes, telomerase, a specialized ribonucleoprotein (RNP), counteracts the inherent loss of telomeres caused by incomplete genome replication by adding G-rich telomeric repeats to the chromosome ends (1). Telomerase upregulation is a hallmark of many cancers, whereas its deficiency is linked to premature aging-related disorders (2, 3). Understanding the structure, mechanisms and regulation of telomerase has profound implications for human health.

Telomerase activity can be reconstituted *in vitro* using two core components: the catalytic subunit telomerase reverse transcriptase (TERT) and telomerase RNA (TR), which provides the template for telomere extension (4). Cellular telomerase holoenzymes incorporate additional protein factors that regulate their biogenesis, subcellular localization, and telomere recruitment (5, 6). Dysfunctions in these holoenzyme subunits disrupt telomerase functions *in vivo*, leading to telomere defects, genome instability and severe premature aging disorders in humans (3).

Beyond humans, single-celled organisms such as ciliates and yeasts have been instrumental in advancing our understanding of telomere biology (7–10). While TERT is highly conserved across eukaryotes, TR has evolved diverse structures with species-specific domains and associated telomerase holoenzyme components (6). Of all described TRs, those found in yeasts rank among the largest (e.g. 1157 nucleotides (nt) in the budding yeast *Saccharomyces cerevisiae*), whereas those of ciliates (e.g. 159 nt in *Tetrahymena thermophila*) and humans (451 nt) are considerably shorter (11). Consequently, yeast telomerase holoenzymes are more complex than their human and ciliate counterparts (10, 12, 13). Recent cryo-electron microscopy (cryo-EM) structures of human and *Tetrahymena* telomerases have revealed their distinct architectures and compositions yet highlighted conserved functions of some TR motifs and associated proteins (14, 15). In contrast, structural characterization of an active yeast telomerase has been hindered by its inherent complexity, low natural abundance and the lack of a robust biochemical reconstitution system. It remains unclear how telomerase maintains its conserved function across species, and whether structural differences reflect fundamentally divergent principles of holoenzyme assembly and regulation.

## *In vivo* reconstitution of budding yeast telomerase yields active holoenzyme

**Fig. 1:**
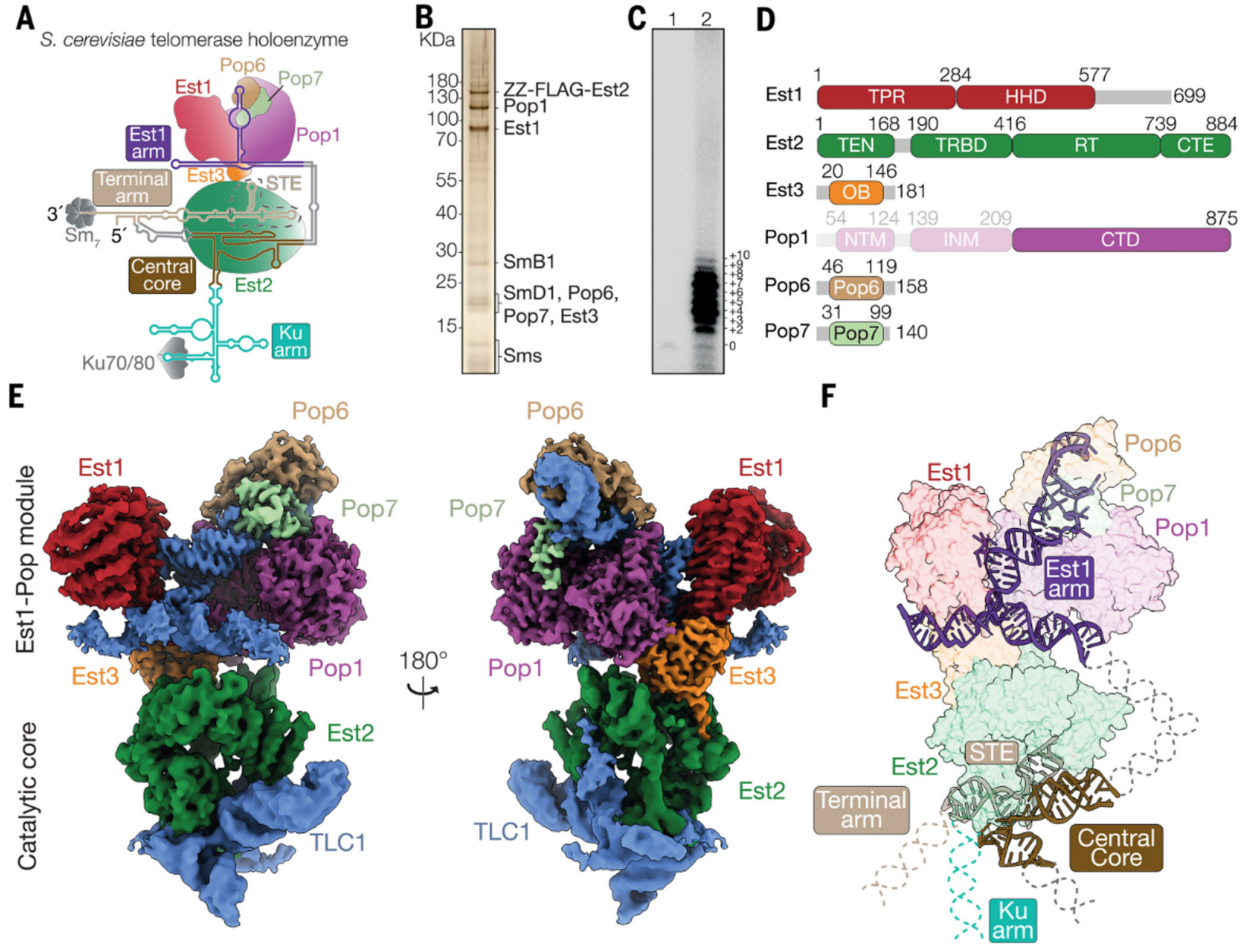
Overall structure of the yeast telomerase holoenzyme. (**A**) Schematic of *S. cerevisiae* (budding yeast) telomerase holoenzyme. (**B**) Silver-stained SDS-PAGE of purified yeast telomerase. (**C**) Telomerase activity assay of the purified yeast telomerase sample. The DNA primer used for the assay was ^32^P-end labelled and loaded in lane 1 without any telomerase to mark the 0 band. The primer extension assay for the purified telomerase sample is shown in lane 2. The ^32^P-end labelled primer shown in lane 1 was also used as a loading control in the telomerase activity assay shown in lane 2. (**D**) Domain architecture of yeast telomerase protein components. TPR, tetratricopeptide repeat-containing subdomain; HHD, helical-hairpin subdomain; TEN, telomerase essential N-terminal domain; TRBD, telomerase RNA-binding domain; CTE, C-terminal extension; RT, reverse transcriptase; OB, oligonucleotide/oligosaccharide-binding domain; NTM, N-terminal motif; INM, internal motif; CTD, C-terminal domain. (**E**) Cryo-EM structure of yeast telomerase holoenzyme in two different views. A composite of a 3.0 Å overall map and a 3.7 Å catalytic core map is shown in the figure. (**F**) Schematic showing the relative positions of protein and RNA components in the yeast telomerase structure. STE, stem-terminus element.

Among yeast species, the composition of *S. cerevisiae* telomerase is one of the most well-characterized. Previous research has identified the 1157 nt RNA component, known as TLC1, along with multiple associated proteins, including the three Est proteins−Est1, Est2 (TERT), and Est3, the heterodimer yKu70/Ku80 (yKu) complex, the heptameric Sm (Sm_7_) complex and the Pop1/Pop6/Pop7 (Pop1/6/7) complex (Fig. 1A) (9, 16–20).

To obtain sufficient *S. cerevisiae* telomerase for structural characterization, we developed a cellular reconstitution system for the complex by co-expressing the limiting Est1, Est2, Est3 and TLC1 in *S. cerevisiae* (fig. S1A) (21). We purified the holoenzyme using an oligonucleotide-based affinity purification via TLC1, followed by FLAG purification through FLAG-tagged Est2 and glycerol gradient centrifugation (Materials and methods, fig. S1, B to E). Silver staining SDS-PAGE, immunoblotting and mass spectrometry confirmed the presence of all holoenzyme components except the yKu complex (Fig. 1B; fig. S1, C to E; Table S1). Previous proteomic studies of yeast telomerase RNP purified using a different method also did not detect yKu proteins, suggesting that their association with the complex is weak or transient (18). A primer-extension assay confirmed that the purified telomerase holoenzyme is catalytically active (Fig. 1C).

## TLC1 scaffolds protein subunits into a Y-shaped core with tethered flexible domains

To gain insight into the architecture of yeast telomerase, we visualized the purified complex using negative stain EM, revealing a Y-shaped structure (fig. S2, A to D). We then used cryo-EM to determine its structure to an overall resolution of 3.0 Å (figs. S3, A to C, S4, A to D; Table S2). This cryo-EM map allowed us to unambiguously identify the three Est proteins and the Pop1/6/7 complex (Fig. 1, D to F; fig. S5, B to I; Table S2). TLC1 features a central core that associates with Est2 and three extended arms that bind Est1, yKu and Sm complexes, respectively (Fig. 1A) (22, 23). These three arms are referred to as the Est1-arm, Ku-arm, and terminal arm. The central core, the Est1-arm and the stem stem-terminus element (STE) as part of the terminal arm of TLC1 are resolved in the cryo-EM map (Fig. 1, E to F; fig. S6, A to D).

3D variability analysis (3DVA) of the consensus reconstruction uncovered considerable conformational heterogeneity in the catalytic subunit, Est2, and the central core of TLC1 with respect to other parts of the holoenzyme (Movie S1, fig. S4D). To improve the resolution of this region for model building, we employed signal subtraction and focused refinement, yielding a 3.7 Å local map for this region (Fig 1E; figs. S3C, S4, E to H, S5A). We built an atomic model consisting of the observed yeast telomerase components based on the 3.0 Å consensus map and the 3.7 Å catalytic core map (Fig. 1F, fig. S4, D and H).

Although the Ku-arm and much of the terminal arm of TLC1, which associate with yKu and Sm_7_ complexes, respectively, are too flexible to be resolved in the consensus reconstruction, their density is evident in the cryo-EM 2D class averages (fig. S3D). Guided by the 2D class averages, we expanded our high-confidence atomic model by incorporating the reported atomic models for Sm_7_ and yKu complexes (24, 25) and the flexible Ku and terminal arms as idealized double-stranded RNA helices to create a plausible model for the entire holoenzyme (fig. S3E).

Telomerase RNA TLC1 has long been proposed to function as a flexible scaffold for protein subunits (22). Consistent with this notion, our structure shows that the yKu and Sm_7_ complexes are flexibly tethered by TLC1. In contrast, despite being connected by flexible linkers, the central core, Est1-arm and STE of TLC1 converge with Est1, Est2 and Pop1/6/7 to form a globular Y-shaped structure. The central core and Est1-arm are essential for telomere maintenance, whereas most of the remaining RNA, except for the Sm binding site, can be deleted without loss of function *in vivo* (26). Together, these findings suggest that formation of the observed globular structure of yeast telomerase is critical for its function.

The globular Y-shaped structure can be divided into two parts: a catalytic core and an Est1–Pop module (Fig. 1, E and F; fig. S6, A to D). In the catalytic core, the reverse transcriptase Est2 binds to the central core and the STE of TLC1. In the Est1–Pop module, Est1 and the Pop1/6/7 complexes are tightly organized around the Est1-arm of TLC1. Est3 does not contact TLC1 directly but stabilizes the entire assembly through extensive protein-protein interactions with Est1, Est2, and Pop1 (Fig. 1E). Our structure highlights the pivotal roles of TLC1 and Est3 in the organization of yeast telomerase.

## The yeast telomerase catalytic core exhibits both conserved and divergent features

**Fig. 2:**
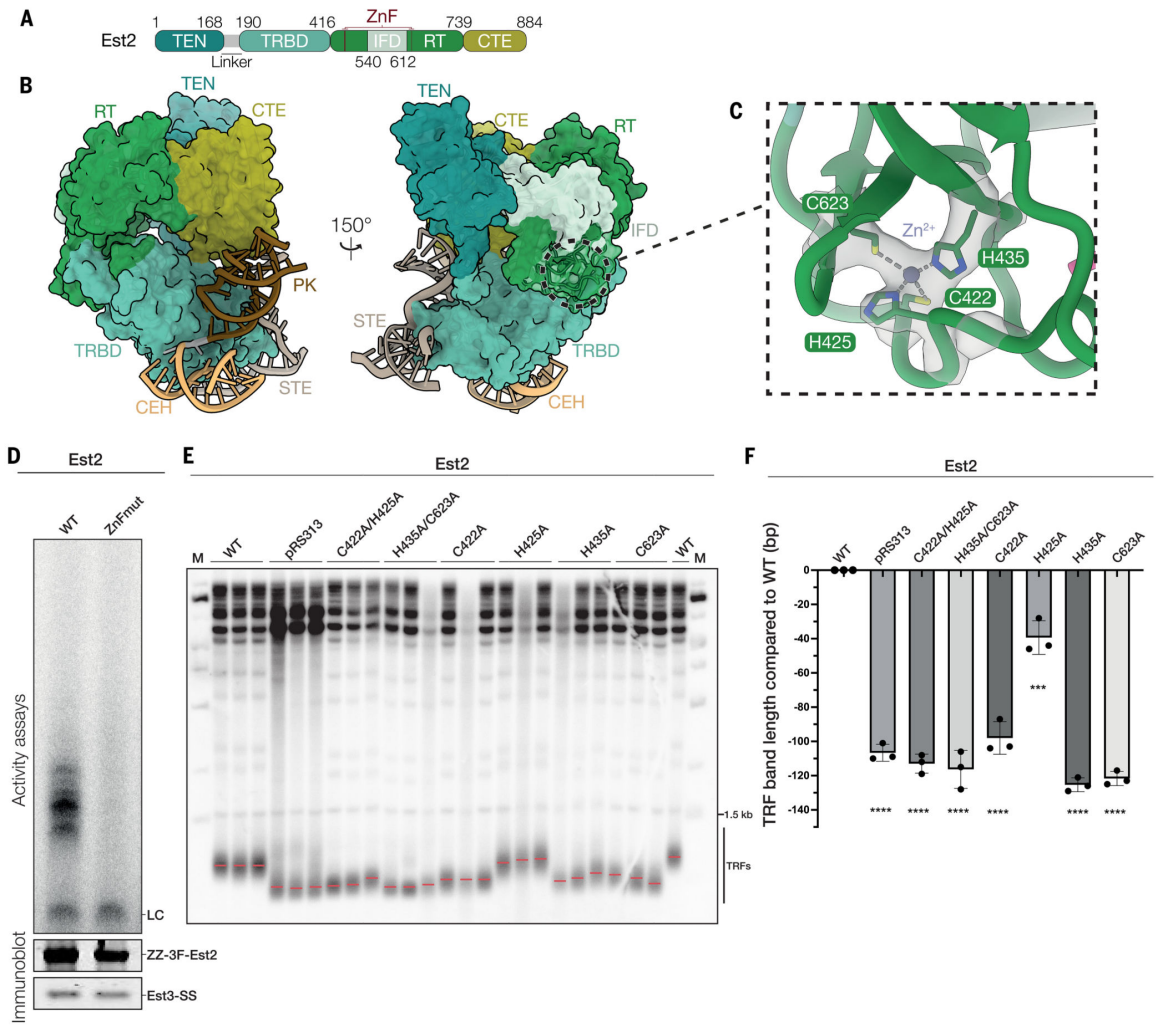
Structure of the yeast telomerase catalytic core. (**A**) Domain organization of Est2. IFD, insertion in fingers subdomain; ZnF, zinc finger. (**B**) Overall structure of the yeast telomerase catalytic core in two different views. CEH, core-enclosing helix; PK, pseudoknot; STE, stem-terminus element. (**C**) Close-up view of the Est2 ZnF modeled into the cryo-EM density. (**D**) Telomerase activity assay of the purified WT (left lane) and the ZnF mutant (right lane) yeast telomerase. Immunoblots detecting Est2 and Est3 levels are shown in the lower panels. These experiments were performed in three biological replicates. See fig. S13A for replicate data. (**E**) Telomere restriction fragment (TRF) assay of *est2Δ* strain expressing either WT Est2 or ZnF mutants or empty pRS313 vector. Red lines indicate the mean telomere length for each lane. M, size markers. For each condition, TRFs from three independent clones are shown. (**F**) Quantification of the telomere length change in Est2 ZnF mutants relative to the WT Est2 in the TRF shown in **E**. Error bars represent standard deviation. ***, *P* ≤ 0.001; ****, *P* ≤ 0.0001.

The catalytic core of yeast telomerase shares a similar overall architecture with the catalytic cores of human and *Tetrahymena* telomerases (15, 27) (fig. S7, A to B). Est2, the catalytic subunit, contains four conserved domains: the telomerase essential N-terminal (TEN) domain, the telomerase RNA binding domain (TRBD), the reverse transcriptase (RT) domain and the C-terminal extension (CTE) (Fig. 2, A and B; figs. S8, A to C, and S9). Est2 adopts a canonical right-hand configuration typical of DNA polymerases, with the RT domain forming the “palm” and “fingers” and the CTE domain forming the “thumb” (fig. S8, D and E). The RT domain also contains an insertion in the fingers subdomain (IFD), which is critical for telomerase processivity (fig. S8F) (27–29).

Within Est2, RNA recognition is mediated by the TRBD and CTE (Fig. 2B). The central core of TLC1 consists of an evolutionary conserved template/pseudoknot and a core-enclosing helix (CEH), linked by a 2-nt junction (Fig. 2B, figs. S6, C and D, S10, A to C). The pseudoknot binds across the Est2 TRBD and CTE, while the 2-nt junction and the CEH interact primarily with the TRBD (Fig. 2B, fig. S10, A to C). Consistent with our structure, TLC1 mutations disrupting the pseudoknot, the 2-nt junction and the 4 base-pair stem at the base of the CEH, each of which directly interacts with Est2, impair telomerase activity *in vitro* and *in vivo* (30–33).

The STE of TLC1, equivalent to conserved regions 4 and 5 (CR4/5) in human telomerase RNA, also binds the TRBD and CTE of Est2 (Fig. 2B; figs. S7, A to B, S10D). However, notable structural and functional differences distinguish the budding yeast STE from the human CR4/5. Unlike the activity-essential human CR4/5, the budding yeast STE is dispensable for telomerase activity in vivo (26, 34). Besides Est2, we found no additional proteins bound to the STE, whereas the human CR4/5 interacts with a histone H2A-H2B dimer (14). The interaction between yeast STE and Est2 is also less extensive than those observed in the human or *Candida* STE–TERT complexes (figs. S7A, S10D) (14, 35). Such divergence has been suggested to have arisen from the coevolution of TERT and TR to adapt to species-specific mechanisms of TR-dependent DNA synthesis (36). Thus, the functional requirement for the STE in budding yeast may have been lost during evolution.

Despite the shared features with the human and *Tetrahymena* catalytic cores, Est2 displays several distinct features. First, Est2 is more compact than both the human and *Tetrahymena* TERTs, exhibiting a smaller IFD, and a shorter, more rigid linker between the TEN domain and the TRBD (fig S8, C and F). A helical element present in the IFD of other TERTs is absent in budding yeast and replaced by a region of Est3 (fig S8F). Second, the Est2–RNA interactions are notably less extensive than those observed in the human and *Tetrahymena* structures (fig. S7A) (14, 15). Third, in previous TERT structures, the TRBD, RT and CTE domains form a ring-like structure, the TERT ring, which encloses the template RNA–DNA substrate duplex (fig. S8B) (14, 15, 37). The ring formation is driven by the extensive interactions between the TRBD and the CTE domain (fig. S8B, middle and right panels). In contrast, in our structure, the TRBD and CTE domain of Est2 form only a single contact (fig. S8B, left panel). This leads to a horseshoe-like configuration for Est2 (fig. S8B, left panel).

Despite adding telomeric DNA substrate in excess to the purified holoenzyme, we did not observe density for the template RNA–substrate DNA duplex in the active site of Est2. This absence persisted even when we used a DNA substrate with locked nucleic acids, which have a methylene bridge between the 2’-oxygen and the 4’-carbon of the ribose group, to enhance duplex stability (38, 39). It is possible that the broader cavity at the center of Est2 (fig. S8B), compared to other TERTs, may not stably accommodate the template-substrate duplex. Such an unstable binding of DNA could explain the low processivity of yeast telomerase *in vitro* (40, 41). Furthermore, the premature dissociation of telomeric DNA from the template RNA would result in the degenerate telomeric repeats (TG_1-3_) commonly observed in yeast telomeres (42–44).

## Est2 contains a zinc finger motif that is important for telomerase function *in vitro* and *in vivo*

We found a zinc finger (ZnF) motif embedded in the conserved “fingers” motifs of the Est2 RT domain, which is absent in reported TERT structures (14, 15, 45) (Fig. 2, A to C; fig. S11, A to D). This ZnF forms an unconventional zinc ribbon structure, where the first β-hairpin provides three zinc ligands (residues Cys422, His425 and His435), and the second β-hairpin contribute to the final ligand (residue Cys623) (Fig. 2C; fig. S11B) (46). Sequence alignment of Est2/TERT shows that residues that form the ZnF motif are conserved among yeast species in the Saccharomycetales and the related Saccharomycodales clades (figs. S9, S12, A and B). We further verified the potential presence of the ZnF motif in some of these Est2/TERT using AlphaFold3 prediction (fig. S12, C to F) (47). Thus, our data suggest that a subset of yeast TERT contains a conserved ZnF motif within the RT domain.

To test the functional importance of the ZnF, we reconstituted and purified telomerase with mutations at the residues that coordinate the Zn^2+^ ion [Est2^C422A/H425A/H435A/C623A^ (ZnFmut), Est2^C422A/C623A^, Est2^H435A/C623A^ and Est2^C422A/H435A^] and examined their activity using primer extension assay. Disrupting the ZnF abolished telomerase activity *in vitro* (Fig. 2D; fig. S13, A and B). We still observed efficient pulldown of Est3 in the purified mutant holoenzymes, suggesting that these mutations did not affect holoenzyme formation (Fig. 2D; fig S13, A and B). To verify whether the defects observed for the ZnF mutant *in vitro* can be recapitulated *in vivo*, we expressed several Est2 ZnF variants with single or double mutation at the zinc-coordinating residues in an *est2Δ* strain and monitored telomere maintenance (Fig. 2E). All strains with Est2 ZnF mutations exhibit significantly shorter telomeres than those in cells expressing wild-type (WT) Est2, some mutations causing telomeres to be as short as in cells with no Est2 (Fig. 2, E and F). The Est2-ZnFmut caused a senescence phenotype after growth for 120 generations at 32 °C (fig. S13, C and D). These observations show that the Est2 ZnF is critical for the catalytic activity of telomerase *in vitro* and in cells.

## Est1 associates with telomerase via interactions with TLC1 RNA

**Fig. 3:**
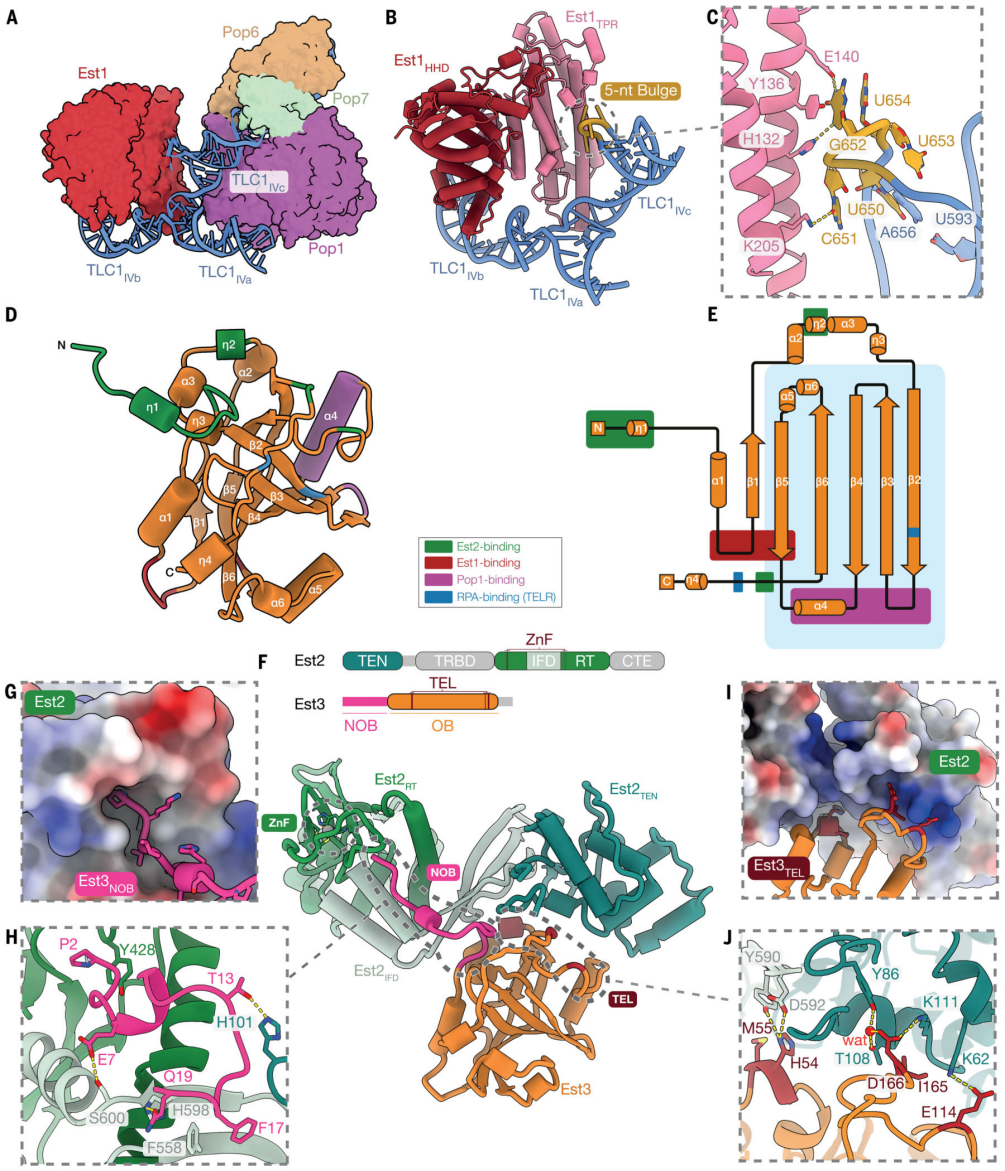
Est1 and Est3 interactions within the holoenzyme. (**A**) Structure of the Est1-arm of TLC1 bound to Est1 and the Pop1/6/7 complex. (**B**) Est1 interaction with TLC1. (**C**) Close-up view of the interactions between Est1 and the TLC1 5-nt bulge in stem IVc of TLC1. (**D**) and (**E**) Structure and topological diagram of Est3 protein, respectively. Est3 motifs that interact Est1, Est2 and Pop1 are mapped on the structure and the diagram in different colors. We also highlight the TELR region that is predicted to interact with replication protein A (RPA) complex (60, 86). (**F**) Domain organization of Est2 and Est3 (top) and interactions between Est3 and Est2 (bottom). Non-interacting domains are shown in grey in the domain schematics, whereas interacting domains are highlighted in other colors. NOB, N-terminus of the OB-fold. (**G**) Electrostatic potentials of the Est3 NOB binding surface on Est2. (**H**) Detailed interactions between the Est3 NOB motif and Est2. (**I**) Electrostatic potentials of the Est3 TEL patch binding surface on Est2. (**J**) Detailed interactions between the Est3 TEL patch and Est2.

Est1 plays a crucial role in telomerase recruitment to telomeres through its interactions with the telomeric protein Cdc13 (48, 49). However, the molecular basis of Est1 association with the telomerase holoenzyme has not been established. In our structure, Est1 interacts with the Est1-arm of TLC1, which encompasses stems IVa, IVb and IVc connected as a three-way junction (Fig. 3, A and B). Est1 consists of a tetratricopeptide repeat-containing (TPR) domain and a helical-hairpin domain (HHD) (fig. S14, A and B) (25). The TPR domain interacts with TLC1 between the IVc stem and the three-way junction, and the HHD domain interacts with the IVb stem (Fig. 3B). These interactions help tether Est1 to the TLC1 RNA and contribute to stabilizing the three-way junction structure.

Previous studies identified a conserved 5-nt bulge (nucleotides 650–654) in the IVc stem that is critical for Est1 association with TLC1 and telomere maintenance *in vivo* (50). In our structure, the co-axial stacking of RNA stems flanking this bulge induces a corkscrew turn, which shows structural similarity to the A-rich bulge structure of the group I intron (Fig. 3C, fig. S14C) (51). The 5-nt bulge is recognized by the TPR domain of Est1 through a combination of RNA backbone and base-specific interactions (Fig. 3C, figs. S5K, S14E). Our structure rationalizes the strict requirement for both primary sequence and length of this bulge structure for telomere maintenance *in vivo*.

Although Est1 is conserved from yeast to humans (fig. S15A), it is not part of the human telomerase holoenzyme (52, 53). In many species, Est1 structural homologs function in nonsense-mediated mRNA decay (NMD), where two Est1 homologs can heterodimerize and recognize phosphorylated UPF1, a central NMD effector, to trigger downstream degradation of target mRNA (54, 55). Comparison of Est1 in our structure with the structure of Est1 homolog SMG5–SMG7 dimeric complex from *Caenorhabditis elegans* shows that the Est1 surface that mediates interactions with the 5-nt RNA bulge is similar to that used for heterodimerization (fig. S14D) (54). This indicates that Est1-like proteins have evolved to utilize a conserved binding interface for interacting with different targets.

## The Pop1/6/7 complex facilitates Est1 association with TLC1 without contacting Est1

The Pop1/6/7 complex, consisting of a large globular protein, Pop1, and a small Pop6–Pop7 heterodimer, is shared between yeast telomerase and the RNase P/MRP complexes (figs. S15, B to D, S16, A and B). While the RNase P/MRP complexes are involved in the processing of tRNA, rRNA and certain mRNAs (56), the Pop1/6/7 complex within budding yeast telomerase was shown to facilitate the assembly and nuclear localization of the telomerase holoenzyme (18, 57, 58). How the Pop proteins are accommodated within the yeast telomerase holoenzyme to fulfil their observed roles was previously unclear.

We find that the Pop1/6/7 complex inserts between the IVa and IVc stems of the TLC1 Est1-arm (Fig. 3A). It binds to a P3 RNA domain within the IVc stem, which is conserved among yeast telomerase, RNase P, and RNase MRP complexes (fig. S16, A and B). Despite the sequence variation in the P3 domain across these three complexes, the structures of their Pop1/6/7-P3 regions share substantial similarities (Fig. S16, A and B), indicating a conserved RNA binding and scaffolding mechanism of the Pop1/6/7 complex. Such similarity explains why substitution of the P3 domain of TLC1 with that of either RNase P or RNase MRP did not affect cell growth or telomere maintenance (18).

Est1 association with telomerase has been shown to depend on both its interaction with the 5-nt bulge and the P3 domain interaction with the Pop1/6/7 complex (Fig. 3, A and B) (58). Despite the lack of direct interactions between Est1 and the Pop1/6/7 complex, our structure reveals a tight organization of the Pop1/6/7 complex and Est1 around the Est1-arm of TLC1 (Fig. 3A). A 2 base-pair insertion between the Est1 binding site and the P3 domain of the Est1-arm would disrupt this organization, leading to a complete loss of Est1 in immunoprecipitated telomerase RNP (58). We also observe that the Pop1/6/7 complex interacts more extensively with the Est1-arm of TLC1 than Est1 (Fig. 3A). Therefore, our structure suggests that the Pop1/6/7 complex facilitates Est1 association with telomerase by scaffolding the Est1-arm of TLC1 into a conformation compatible for Est1 binding.

## Est3 forms a protein-protein interaction hub at the heart of telomerase

Est3 is a small oligonucleotide/oligosaccharide-binding (OB)-fold protein that has been proposed to be a structural homolog of the human TPP1 protein, a component of the shelterin complex that associates with telomeres (59, 60). In humans, TPP1 transiently interacts with TERT to recruit telomerase to telomeres *in vivo* (61–64). Although several prior studies have suggested a role for Est3 in stimulating telomerase activity (65–67), the structural basis underlying its function was unknown. Our structure reveals that Est3 forms a central interaction hub, binding to Est1, Est2 and Pop1 proteins through several separate interfaces. Given that Est1 and Pop1 are not present in human telomerase, our structure suggests a more complex regulatory role for Est3 in yeast telomerase than just being a TPP1 homolog (Figs. 1E, 3, D and E).

## Est3–Est2 interactions are more extensive than those of human TPP1–TERT

Est3 binds to Est2 using a patch surface on the core OB-fold domain and an extended region at the N-terminus of the OB-fold (residues 2–19) (Fig. 3, E and F). These two regions of Est3 are analogous to the TPP1 glutamate (E) and leucine-rich (TEL) patch and the N-terminal region of TPP1 OB-fold domain (NOB) that are crucial for telomerase recruitment and activation in humans (Fig. 3, F to J; fig. S5, L and M) (61, 64). For consistency, we name them the TEL patch and the NOB of Est3, respectively (Fig. 3F). Our structure supports the notion that Est3, at least in this regard, is functionally equivalent to TPP1 (fig. S17, A to C).

Previous studies defined a TEL surface on Est3 (Val75, Tyr78, Thr112, Glu114, Asn117, Asp166 and Val168) that is important for telomere maintenance *in vivo* (figs. S17, D and E, S18) (60). This surface also covers the Est3 interaction site with Pop1 in our structure (Fig. 3, D and E, figs. S5J and S17E) (see the next section). Thus, we re-defined the TEL patch to include residues that interact with Est2 (His54, Met55, E114, Ile165 and D166) (Fig. 3, F, I and J). The Est3 TEL patch docks onto the positively charged surfaces of Est2, interacting with both the TEN domain and the IFD (Fig. 3, I and J).

**Fig. 4:**
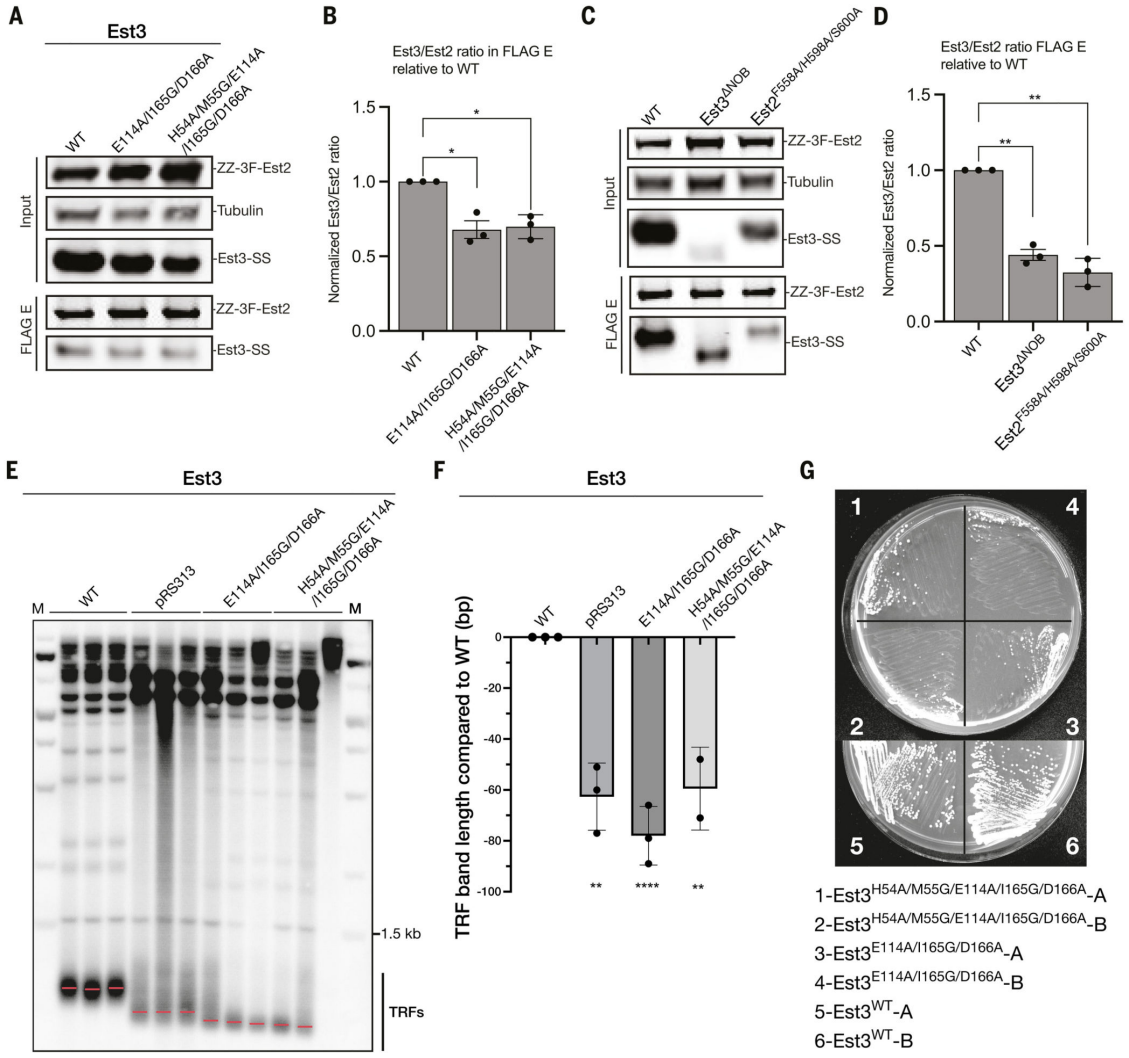
Interactions between Est2 and Est3 are critical for telomerase function. (**A**) Co-IP assays of FLAG-tagged Est2 with Strep-tagged WT Est3 or Est3 TEL patch mutants. Immunoblots detecting Est2 and Est3 levels using antibodies against FLAG and Strep tags, respectively, are shown. (**B**) Quantification of Est3 signal relative to Est2 in the FLAG elution of the co-IP data in **A**. The Est3/Est2 ratios were calculated from the intensities of the Est3 and Est2 bands in the FLAG elution. The Est3/Est2 ratios of the Est3 mutants were then normalized against that of the WT Est3. (**C**) Co-IP of FLAG-tagged Est2 and Strep-tagged Est3 where the Est2-Est3-NOB interactions are disrupted using either Est3^ΔNOB^ mutant or Est2^H598A/S600A/F558A^. Immunoblots detecting Est2 and Est3 levels using antibodies against FLAG and Strep tags, respectively, are shown. (**D**) Quantification of Est3 signals relative to Est2 in the FLAG elution of the co-IP data in **C**. The method for quantification is the same as stated for **B**. (**E**) TRF assays of *est3Δ* strain expressing either WT or mutant Est3 or empty pRS313 vector. Red lines indicate the mean telomere length for each lane. For each condition, TRFs from three independent clones are shown. (**F**) Quantification of the telomere length change in Est3 mutants relative to the WT Est3 in the TRF shown in **E**. (**G**) Yeast growth assay of *est3Δ* strain expressing either WT or mutant Est3. Yeasts were plated after propagating for 100 generations. Experiments shown in **A** and **C** were performed in biological triplicate (see fig. S19 for replicate data). Error bars shown in **B, D** and **F** represent standard deviation. *, *P* ≤ 0.05; **, *P* ≤ 0.01; ****, *P* ≤ 0.0001.

To validate the observed Est2–Est3 interaction, we expressed telomerase variants carrying mutations in the Est3 TEL patch (Est3^E114A/I165G/D166A^ and Est3^H54A/M55G/E114A/I165G/D166A^) or a deletion of the NOB (Est3^ΔNOB^) and performed co-immunoprecipitation (co-IP) assays with Est2. In support of our structure, these Est3 mutations reduced the level of Est3 co-purified with Est2 compared to the WT Est3 (Fig. 4, A to D, fig. S19, A and B).

To further probe the functional importance of the Est3 TEL patch, we expressed the Est3 TEL patch mutants (Est3^E114A/I165G/D166A^ and Est3^H54A/M55G/E114A/I165G/D166A^) in an *est3Δ* strain. These mutants exhibit telomere shortening and senescence *in vivo* (Fig. 4, E to G). Consistent with our data, previous work showed that mutations at several residues on the TEL patch of Est3 led to reduced Est3 association with telomerase and telomere length defects *in vivo* (59, 60, 65, 68, 69). We also mutated residues in Est2 (Est2^K62E/K111E^) that interact with the Est3 TEL patch (Fig. 3J). This Est2^K62E/K111E^ mutant displayed telomere shortening and a senescence phenotype, mirroring the Est3 TEL patch mutations (fig. S20, A to C). Although the previously reported Est2^K111A^ mutant is expressed at lower levels than wild type, it shows only mild telomere shortening and no senescence (70). The strong phenotype of the Est2^K62E/K111E^ mutant, therefore, indicates that interactions between the Est3 TEL patch and both K62 and K111 of Est2 are essential for telomerase function *in vivo*.

Unlike the TEL patch, the functional role of the NOB in Est3 has not been characterized previously. Expression of the Est3^ΔNOB^ mutant in the *est3Δ* strain also led to a senescence phenotype (fig. S20D). However, this protein was expressed at a much lower level than that of the WT Est3 (fig. S20E), making it unclear whether the observed phenotype was due to the disrupted Est2–NOB interaction or the lower Est3^ΔNOB^ expression. Thus, we mutated Est2 residues that interact with the Est3 NOB instead (Est2^F558A/H598A/S600A^) (Fig. 3H). This Est2^F558A/H598A/S600A^ mutant exhibited reduced Est2–Est3 interaction and telomere shortening but did not senesce (Fig. 4, C and D and fig. S20, A to C). Our results, therefore, indicate that the interaction between the Est3 NOB and Est2 is important for telomerase function *in vivo*.

Compared to TPP1 and the *Tetrahymena* homolog p50, the Est3 NOB is longer and interacts more extensively with Est2 (Fig. 3, F to H; fig. S17C). Besides interacting with the IFD and the TEN domain of Est2 as observed previously for TPP1 and p50 (15, 27, 71), the NOB of Est3 extends towards the RT domain and is anchored to the hydrophobic cavity formed by the Est2-ZnF (Fig. 3, F and G). AlphaFold3 prediction of Est2–Est3 complexes in different yeast species suggests that the interaction between the ZnF of Est2 and the NOB of Est3 is conserved in the Saccharomycetales clades (fig. S21, A and B).

### Pop1 stabilizes Est2-Est3 interaction

**Fig. 5:**
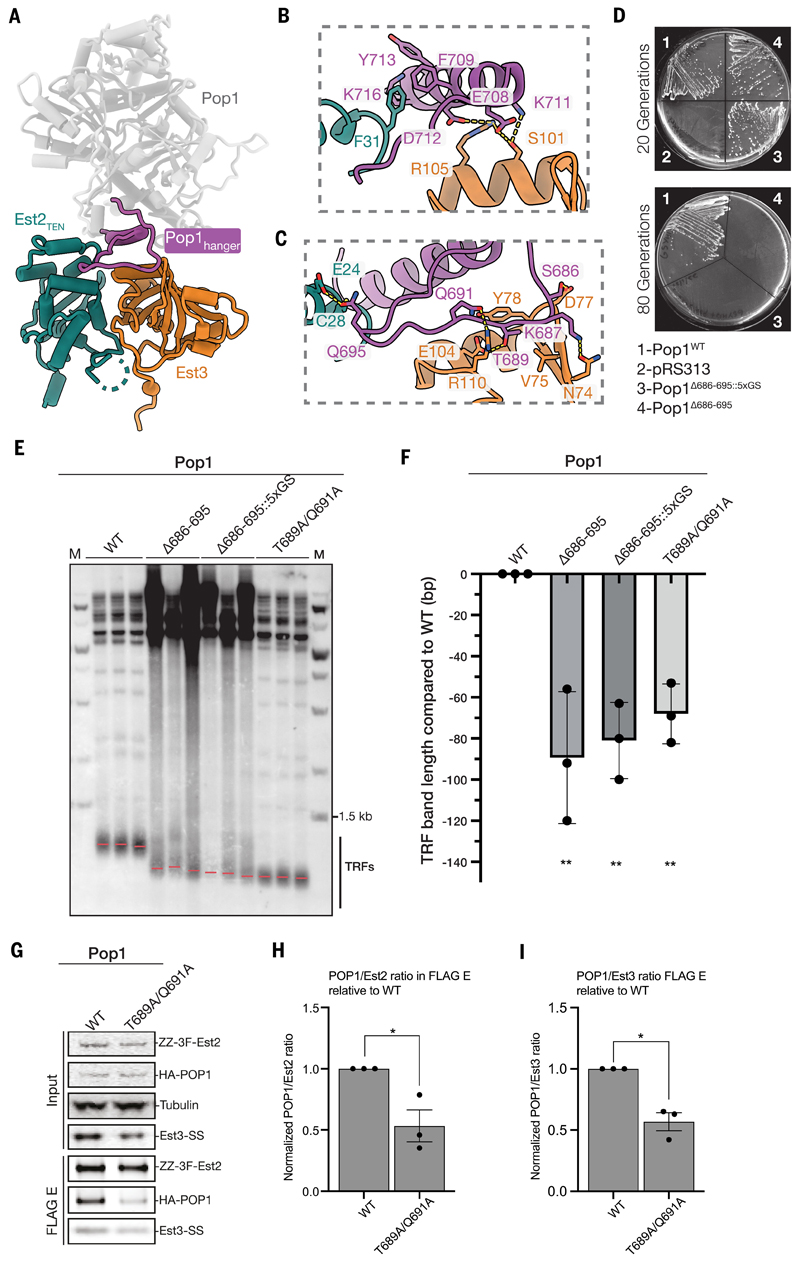
The Pop1-hanger is essential for telomere maintenance. (**A**) Overall view of Pop1–Est2– Est3 interactions. The Pop1-hanger that interacts with both the TEN domain of Est2 and Est3 is highlighted in purple while the rest of Pop1 is colored in grey. (**B**) and (**C**) Close-up view of the interactions between Pop1-hanger with Est3 and Est2 TEN domain. (**D**) Yeast growth assay of *pop1Δ* strain expressing either WT or mutant Pop1 or empty pRS313 vector. The number of generations is indicated. See fig. S22A for intermediate generations. (**E**) TRF assay of *pop1Δ* strain expressing either WT or mutant Pop1. Red lines indicate the mean telomere length for each lane. Yeasts were grown for 100 generations before the TRF assay. For each condition, TRFs from three independent clones are shown (**F**) Quantification of the telomere length change in Pop1 mutants relative to the WT Pop1 in the TRF shown in **E**. (**G**) Co-IP assays of FLAG-tagged Est2 with HA-tagged Pop1 and Strep-tagged Est3. In these assays, WT Est2 and Est3 are expressed in *pop1Δ* strain expressing either WT or Pop1^T689A/Q691A^ mutant. Immunoblots detecting Est2, Est3 and Pop1 levels using antibodies against FLAG, Strep and HA tags, respectively, are shown. (**H**) and (**I**) Quantification of Pop1 relative to Est2 and Est3, respectively, in the FLAG elution of the co-IP data in **G**. The Pop1/Est2 and Pop1/Est3 ratios were calculated from the intensities of the Pop1, Est2 and Est3 bands in the FLAG elution. The resulting ratios of Pop1^T689A/Q691A^ mutant were then normalized against that of the WT Pop1. Experiments shown in **G** were performed in biological triplicate (see fig. S22B for replicate data). Error bars shown in **F, H**, and **I** represent standard deviation. *, *P* ≤ 0.05; **, *P* ≤ 0.01; ****, *P* ≤ 0.0001.

Our structure reveals an unexpected role of Pop1 in facilitating Est2–Est3 interaction. Pop1 consists of an N-terminal motif (NTM), an internal motif (INM) and a large C-terminal domain (CTD) (Fig. 1D). Only the Pop1 CTD domain is visible in our structure. Notably, we observe a long triangle-shaped loop (residues 686–695), along with a perpendicular helix on its backside (residues 702–720), protruding from the core of the Pop1 CTD domain to interact with both Est3 and the TEN domain of Est2 (Fig. 5, A to C; fig. S5J). We refer to this region as the “Pop1-hanger” due to its distinctive shape (Fig. 5, A to C).

In agreement with our observed Pop1–Est2 interaction (Fig. 5, B and C), previous work reported that deletion of the P3 domain of TLC1, which associates with Pop1/6/7, destabilizes Est2 binding to TLC1 (58). Mutation at several Est3 residues that are involved in Pop1 interaction, including V75, Y78 and E104 (Fig. 5, B and C), have been shown to cause telomere length defects and reduction of Est3 association with telomerase (59, 60). These data suggest that the observed Pop1–Est3 interaction also stabilizes Est3 association with telomerase.

The Pop1-hanger is disordered in the reported RNase P and MRP structures, suggesting that it has a specific role in telomere maintenance rather than RNA processing (72, 73). To test this notion, we expressed Pop1, in which the Pop1-hanger was either deleted (Pop1^Δ686–695^) or replaced with a 5xGS linker (Pop1^Δ686–695::5xGS^) in a *pop1Δ* strain. These mutants complement the lethality of the *pop1Δ* strain, suggesting that they are functional in RNA processing pathways performed by RNase P and RNase MRP. However, they exhibited telomere length defects and senesced after 60 to 80 generations (Fig. 5, D to F and fig. S22A). Point mutations in the Pop1-hanger at Thr689 and Gln691 (Pop1^T689A/Q691A^), which disrupt interaction with Est3, also resulted in telomere shortening (Fig. 5, E and F). Taking advantage of the milder growth defects of the Pop1^T689A/Q691A^ mutant compared to Pop1^Δ686–695^ and Pop1^Δ686–695::5xGS^ mutants, we overexpressed telomerase in this mutant and performed co-IP with Est2. Consistent with our structural data, we observed a reduction of the level of Pop1 relative to Est2 and Est3 in the elution fraction (Fig. 5, G to I, fig. S22B). Therefore, our data demonstrate that the stabilization of Est2–Est3 interaction by the Pop1-hanger is essential for telomere maintenance *in vivo*.

### Est3 interacts with Est1 but is not required for Est1 association with telomerase

Est1–Est3 interactions have been shown to be crucial for recruiting Est3 to telomeres, but the molecular details of the interactions were unknown (74–76). Our structure shows that Est1 contacts Est3 through two extended α-helical hairpins located in its TPR domain, adjacent to the binding interface between Est1 and TLC1 (fig. S23, A and B). To test the functional importance of the observed Est1–Est3 interaction, we disrupted the Est1–Est3 interface *in vivo* by mutating residues in the α1–α2 and α3–α5 hairpins of Est1 (Est1 ^R29A/D33A/N35A^ and Est1^D104A/R112A^) (fig S23, C to E). Est1^R29A/D33A/N35A^, but not Est1^D104A/R112A^, exhibit shorter telomeres compared to WT Est1 when expressed in an *est1Δ* strain. These mutants also do not senesce. These observations suggest that this particular Est1–Est3 interaction is not critical for Est1 function in telomere maintenance. Given that Est1 extensively interacts with TLC1 (Fig. 3, A to C, fig. S14E), a disruption of the Est1 interaction with Est3 does not substantially affect Est1 association with telomerase.

## Zinc finger motifs are predicted in TERTs of many other eukaryotic lineages

Due to structural and mechanistic similarities between TERT and retrotransposon reverse transcriptases, telomerase is thought to have evolved from ancient retroelements (77, 78). Recent structures of the reverse transcriptase from the silkworm *Bombyx mori* R2 and human LINE-1 retrotransposons also revealed ZnF motifs, adding another common feature between TERT and retrotransposon reverse transcriptases (79–82). To investigate whether ZnF motifs are present in TERTs beyond yeast, we used AlphaFold3 to predict the structures of TERTs from different eukaryotes, in addition to zinc ion as a ligand (Table S3) (47). We identified ZnF motifs in TERTs from diverse lineages, notably plants, insects, kinetoplastids, dinoflagellates and oomycetes (fig. S24; Table S3). However, the positions of the predicted ZnF motifs vary among species, suggesting that they may have arisen through independent evolution events.

## Discussion

Here we present the structure of the budding yeast telomerase holoenzyme, revealing an intricate assembly that diverges markedly from its human and ciliate counterparts. Our structure not only rationalizes numerous prior observations but also offers unprecedented insights into the specific functions of components within the yeast telomerase complex.

Our work provides a molecular framework for understanding the role of the Pop1/6/7 complex in telomerase. Given its stable, co-transcriptional association with TLC1 independent of other telomerase subunits (83), we propose that the Pop1/6/7 complex serves as an “assembly module” that facilitates the assembly of all three Est proteins in yeast telomerase (fig. S25A). The stabilizing effects of Pop1/6/7 on Est2 and Est3 are primarily mediated by protein-protein interactions (Fig. 5, A to C), which explains why relocating the entire Est1 arm of TLC1 to different regions of the TLC1 still preserves telomerase function *in vivo* (22). A similar acquisition of a multifunctional assembly module has been observed in vertebrate telomerase, where the H/ACA RNP components are shared with the pseudouridylation machineries such as snoRNPs and scaRNPs (fig. S25B) (84, 85). Collectively, our biochemical, structural and genetic data (Fig. 1, B and E, 3A, 5, A to F; fig S1, C and E) support the classification of Pop1/6/7 as a bona fide holoenzyme component.

We identified a conserved ZnF motif within TERT (Est2) in the Saccharomycetales and Saccharomycodales clades. Although the RNA template is not resolved in our structure, comparison with human TERT reveals that the ZnF lies adjacent to a positively charged surface that would interact with the 5′ region of the RNA template (fig. S11, C and D). Disrupting the ZnF would reduce, but not abolish, the binding of the RNA template because the template can still engage with residues within this positively charged channel in Est2. This explains the phenotype of the ZnF mutants, which exhibits shortened telomeres without entering senescence. We propose that the ZnF motif in Est2 contributes to the stabilization of the RNA template within Est2.

Alongside the structures of human and *Tetrahymena* telomerases, our findings underscore the diverse evolutionary strategies telomerase RNPs have adopted to enable holoenzyme assembly and regulation while maintaining conserved catalytic function (fig. S25, A to C). Although TR has undergone substantial evolutionary divergence (36), TERT evolution is thought to be monophyletic. Yet our discovery of ZnF motifs in diverse TERTs, including one in Est2 potentially involved in RNA template binding, suggests the evolutionary ingenuity of TERT to accommodate structurally diverse TRs.

Despite their evolutionary divergence, yeast, ciliate and human telomerase are all regulated by multiple OB-fold proteins. First, Est3, a single OB-fold protein, has at least three protein binding partners and can modulate telomerase through multiple mechanisms (Fig. 3, D and E). By acting as a central hub for protein-protein interactions, Est3 is crucial for the stability of yeast telomerase. Est3 can also enhance telomerase catalytic activity via direct interaction with Est2, in a manner analogous to TPP1 in human telomerase. This interaction likely accounts for the observed increase in telomerase activity in *S. cerevisiae, Candida albicans*, and *Saccharomyces castelli in vitro* (65–67). Moreover, recent studies show that Est3 cooperates with RPA to enhance telomerase activity by stabilizing the telomeric DNA substrate on telomerase (86).

Second, Est1 recruits telomerase to telomeres during late S-phase by interacting with Cdc13, another OB-fold containing protein, which binds the 3′ single-stranded telomeric DNA. The Cdc13–Est1 interaction interface is oriented away and spatially distant from the Est2 active site (Fig. 3A, fig. S23F), consistent with its role primarily in recruitment rather than catalysis. However, this spatial separation raises the question of how the telomeric DNA is coordinated between Cdc13 and Est2. Finally, with the precise timing of RPA interaction with telomerase remaining unclear, these findings point to a dynamic coordination between Est1, Cdc13, RPA, and the telomerase core in handling the telomeric DNA substrate during recruitment and extension.

Within the spectrum of RNP flexibility, yeast telomerase occupies the highly dynamic end, alongside other RNPs that contain long non-coding RNAs, pre-mRNAs and mRNA (13). Yeast telomerase, therefore, serves as model for understanding how a multi-subunit RNP can assemble on a large and flexible RNA scaffold. Although the predicted secondary structure of TLC1 features three similar long RNA arms protruding from the central core, our structure reveals that the Est1-arm and the terminal arm fold back, and integrate into a globular core together with its associated proteins (Fig. 1, A and F). In contrast, the Ku-arm and the Sm binding site of the terminal arm remain flexible extensions. Thus, TLC1 indeed functions as a scaffold to organize protein subunits, as predicted (22), but it does so through two distinct mechanisms that cannot be deduced from secondary structure predictions alone. This architectural principle, combining a globular RNA-protein core with flexible RNA extensions, may represent a common organizational theme among other highly flexible RNPs.

## Materials and Methods

### Yeast strains

*S. cerevisiae* strain [*MATα pep4*::*HIS3 prb1::LEU2 bar1::HIS6 lys2::GAL1/10-GAL4 can1 ade2 trp1 ura3 his3 leu2-3*,*112*] was used for overexpression of telomerase holoenzyme for purification and structural studies (87). The strains EDL221 (*est1Δ::TRP1*), EDL223 (*est3Δ::TRP1)*, JNY262 (*est2Δ::TRP1*) and GSYH12 (*pop1Δ::KanMX*) were used for senescence assays and telomere length analyses of the different *EST1, EST2, EST3* and *POP1* mutants. The pRS313 plasmids (*CEN HIS3*) expressing mutants of *EST1, EST3, EST2* or *POP1* were transformed in EDL221, EDL223, JNY262 or GSYH12, respectively, complemented with wild-type *EST1, EST2* or *POP1* in pRS316 (*CEN URA3*) or YCPlac33-*EST3* (*CEN URA3*). Loss of complementing plasmid was selected on 5-fluoroorotic acid plates without histidine, and senescence was assessed by successive streaks on plates with synthetic media without histidine at 30°C. All the yeast strains used in this study are listed in Table S4.

### Molecular cloning

*S. cerevisiae* Est1, Est2, and Est3 gene fragments were PCR amplified from yeast genomic DNA. The frameshift in the Est3 open reading frame was corrected using site-directed mutagenesis. Genes were then individually inserted into yKN plasmids containing a GAL-GAP promoter (21). For Est2, a ZZ-FLAG sequence was inserted in the 5′ region of the gene. The resulting three plasmids were then assembled into a single pRS426 vector using Gateway cloning method (Invitrogen, Cat# 11791020). Gene encoding yeast TLC1 was cloned under a GAL1 promoter and ligated into a pRS314 vector. Plasmids containing Est2 and Est3 mutations were constructed by PCR-based site-specific mutagenesis using Phanta Flash DNA polymerase (Vazyme, Cat# P510-01). For endogenous expression of Est1, Est2, Est3 or Pop1 mutant proteins, their genes were cloned with their promoter and 3′ UTR in pRS313 vector and mutagenized using gene mutagenesis services at GenScript. Plasmids YCPlac33-*EST3-FLAG*_*3*_ and pRS313-*EST3-FLAG*_*3*_ were created by subcloning the *EST3* frameshift-corrected gene with 3 C-terminal FLAG tags from pVL2076 (*CEN LEU2 EST3-FLAG*_*3*_) from V. Lundblad lab (59). Mutations were confirmed by Sanger sequencing. Plasmids and oligonucleotides used in this study are listed in Tables S5 and S6, respectively.

### Yeast transformation, growth and lysis

Yeast cells were co-transformed with pRS314-TLC1 and pRS426-ZZ-FLAG-Est2-Est1-Est3 plasmids using the LiAc/single-stranded carrier DNA/PEG method, as previously described (88). Yeast cells overexpressing ZZ-FLAG-Est2, Est1, Est3 and TLC1 were first grown in YM4 medium supplemented with 1% (w/v) raffinose at 30 °C overnight and then inoculated into 800 ml YM4 medium supplemented with 1% (w/v) raffinose to OD_600_ of 0.3. Yeast cells were grown at 30 °C until OD_600_ reached 1.0, then galactose was added to the media at a final concentration of 2% (w/v) to induce protein expression. Cells were then grown overnight and harvested at an OD_600_ of 4.0, resuspended with resuspension buffer [200 mM NaCl, 100 mM HEPES NaOH pH 8.0, 2 mM MgCl_2_, 10% glycerol, 0.2% IGEPAL CA-630, 2 mM DTT, 0.4 mM PMSF, cOmplete protease inhibitor tablets (Roche, Cat# 11873580001)] at a 1:1 ratio. Yeast cells were frozen in liquid nitrogen and lysed using freezer mill (SPEX Sample prep Freezer/Mill 6870D).

### Yeast telomerase purification

For WT yeast telomerase purification, yeast cell lysate from 48 L of yeast was thawed and centrifuged at 4 °C 40,000 rpm for 1 h using Type 45 Ti Rotor (Beckman Coulter). Pierce™ High Capacity Streptavidin Agarose (Thermo Fisher Scientific, Cat# 20357) was pre-coupled with 5’-biotinylated antisense template oligonucleotide (5’-biotin-CTAGACCTGTCATTTGUGUGUGGGUGUG-3’, where nucleotides without underlining are DNA, and underlined nucleotides are 2′-*O*-methyl RNA) and then blocked with 0.5 mg/ml biotin solution. After centrifugation, supernatants were pooled and incubated with resin for 2 h at room temperature. The resin was then washed with wash buffer [150 mM NaCl, 20 mM HEPES NaOH pH 8.0, 2 mM MgCl_2_, 10% glycerol, 0.1% IGEPAL CA-630, 1 mM DTT, 0.2 mM PMSF, cOmplete protease inhibitor tablets (Roche, Cat# 11873580001)], and then eluted for 2 h at room temperature with wash buffer containing 30 μM displacement oligo (5’-CACACCCACACACAAATGACAGGTCTAGddC-3’, where ddC stands for 3’-dideoxycytidine). Eluates were incubated with DYKDDDDK Fab-Trap™ Agarose (ChromoTek, Cat# ffa-20) at 4 °C overnight. Resin was washed with wash buffer, and incubated with buffer containing 10 μM 21 nucleotide (nt) DNA substrate (5’-TAGTAGTGTGTGGTGTGTGGG-3’) at 4 °C for 45 min. Resin was further washed and the protein complex was eluted at 4 °C for 3 h using wash buffer containing 500 μg/ml 3xFLAG peptide (ThermoScientific, Cat# A36805). FLAG eluates were concentrated using 50 kD cutoff centrifugal concentrator (Amicon, Cat# UFC505096). Concentrated sample was then applied onto a 200 μl 10%-30% (v/v) glycerol gradient and centrifuged at 55,000 rpm for 1 h in a TLS-55 rotor (Beckman Coulter). Gradient was manually fractionated to 16 μl per fraction, and the peak fractions was pooled and concentrated. Concentrated sample was cross-linked with 0.5 mM BS^3^ (Thermo Fisher Scientific, Cat# A39266) on ice for 1 h. Reaction was quenched by adding quench buffer [150 mM NaCl, 200 mM Tris-HCl pH 8.0, 2 mM MgCl_2_, 10% glycerol, 0.1% IGEPAL CA-630, 1 mM DTT, 0.2 mM PMSF]. Sample was dialyzed in dialysis buffer [150 mM NaCl 200 mM HEPES NaOH pH 8.0, 2 mM MgCl_2_, 1% glycerol, 0.05% IGEPAL CA-630, 1 mM DTT, 0.2 mM PMSF] at 4 °C for 2 h before cryo-EM sample preparation.

Purification of Est2 mutants followed oligo-based purification and FLAG tag purification described above. FLAG eluates from each mutant and the WT control were concentrated to 30 μl, and then aliquoted to 5 μl per tube.

### Co-immunoprecipitation of Est2 with Est3 and/or Pop1

For co-IP experiments of Est2 with Est3 shown in Fig. 4, A and C, BCY123 yeast strain was co-transformed with pRS314-TLC1 and pRS426-ZZ-FLAG-Est2-Est1-Est3-SS plasmids bearing respective Est2 or Est3 mutations. For co-IP experiments of Est2 with Pop1 mutants shown in Fig. 5G, GSYH12 strains bearing a Pop1 mutant plasmid were transformed with pRS314-TLC1 and pRS426-ZZ-FLAG-Est2-Est1-Est3-SS plasmids.

For all co-IP experiments, yeast lysates were incubated with 10 μL DYKDDDDK Fab-Trap™ Agarose (ChromoTek, Cat# ffa-20) at 4 °C overnight. Resin were then washed and eluted at 4 °C for 2 h using wash buffer containing 500 μg/ml 3xFLAG peptide (ThermoScientific, Cat# A36805). FLAG eluates were concentrated to 30 μL using 50 kDa cutoff centrifugal concentrator (Amicon, Cat# UFC505096). Concentrated eluates were aliquoted to 5 μL/tube for immunoblotting. In all co-IPs, Est2, Est3 and Pop1 were added in excess of the beads as examined by the immunoblots of the FLAG flow-through (FLAG-FT) fractions (figs. S19, A and B, S22B). This ensures that similar amounts of Est2 were captured by the beads in each experiment and the levels of Est3 and Pop1 co-purified with Est2 are not limited by its initial expression.

### Immunoblotting of the reconstituted yeast telomerase RNP

For immunoblotting, proteins were first resolved on a 4-12% Bis-Tris NuPAGE gel (Invitrogen, Cat# NP0323BOX). The gel was then transferred onto a nitrocellulose membrane at 500 mA current for 1 h. The membrane was incubated in 5% non-fat milk in phosphate buffered saline (PBS) supplemented with 0.1% Tween-20 (PBST) at room temperature for 1 h. Afterwards, the membrane was incubated with a mixture of mouse anti-FLAG antibody (Proteintech, Cat# 66008-4-Ug, lot 10027647, 1:1000 dilution), rabbit anti-strep antibody (Abcam, Cat# ab76949, lot GR3424188-4, 1:5000 dilution), rabbit anti-alpha tubulin (Abcam, Cat# Ab184970, lot 1123403-1, 1:10000 dilution), and rabbit anti-HA antibody (Abcam, Cat# ab9110, lot 1100696-5, 1:1000 dilution) in PBST with 5% non-fat milk. The membrane was washed in PBST and incubated with a mixture of goat anti-mouse Alexa-Fluor 680 antibody (Abcam, Cat# ab175775, lot GR3273649-2 and 1027918-7, 1:10000 dilution) and goat anti-rabbit Alexa-Fluor 790 (Abcam, Cat# ab175781, lot GR226409-8, 1:10000 dilution) in PBST with 5% non-fat milk at room temperature for 1 h. The membrane was washed with PBST and imaged on Li-COR Odyssey Imager. Quantifications of immunoblots were conducted using ImageJ. Data were analyzed and statistical test was performed using Graphpad Prism (Graphpad). Paired Student’s t-test was used for the statistical analysis, and standard deviations (SD) were shown in the graphs.

### Mass spectrometry

Purified yeast telomerase sample was denatured at 80 °C for 10 min in the presence of RapiGest (Waters), then reduced by adding a final concentration of 4 mM DTT, and incubated at 60 °C for 10 min. The sample was alkylated by adding iodoacetamide to a final concentration of 14 mM and digested with trypsin (Promega) at 37 °C overnight. The sample was acidified with 2 μl trifluoroacetic acid (TFA) and incubated at 37 °C for 45 min. The protein solution was then desalted using C18 stage tips (3M Empore). The extracted peptides were analyzed by liquid chromatography (LC)–MS/MS on a Q Exactive HF-X mass spectrometer (Thermo Fisher Scientific) equipped with an Ultimate 3000 HPLC (Thermo Fisher Scientific). Data were subsequently analyzed and processed in Proteome Discoverer v 3.1 (Thermo Fisher Scientific).

### Telomerase activity assays

5 μl of concentrated yeast telomerase samples were each mixed with 15 μl reaction buffer [50 mM NaCl, 40 mM Tris pH 8.0, 2.5 mM MgCl_2_, 0.5 mM spermidine, 5% glycerol, 1 mM DTT, 250 μM dTTP, 250 μM dATP, 5 μM unlabelled dGTP, 0.15 μM α-^32^P-dGTP (3,000 Ci mmol^-1^, 10 mCi ml^-1^) (Hartmann Analytic Gmbh, Cat# FP204), 500 nM 21 nt substrate DNA (5′-TAGTAGTGTGTGGTGTGTGGG-3′)]. Reactions were performed at 30 °C for 1h and were stopped by adding 80 μl TES buffer (50 mM Tris HCl pH 8.5, 20 mM EDTA and 0.2% SDS). Nucleic acid extraction was performed with phenol:chloroform:isoamyl alcohol (Thermo Scientific, Cat# 17908), and the samples in the aqueous phase were mixed with a ^32^P-labelled 18 nt oligonucleotide as an internal loading control (LC). Samples were then precipitated with ethanol. Pellets were resuspended in water and RNA loading dye, boiled for 98 °C 10 min, and resolved on a 10.5% denaturing polyacrylamide (19:1) TBE gel at 500 V for 2 h. Gels were dried at 80 °C for 1 h and exposed to a phosphor-imaging screen (BAS-IP MS 2040, Fuji Film). The screen was imaged by using Amersham Typhoon Biomolecular Imager (Cytiva) and quantified using ImageJ. For Est2 ZnF mutants (C422A/H425A/H435A/C623A, C623A/C422A, C623A/H435A and C422A/H435A), three independent biological replicates were performed for the activity assay.

### Genomic DNA isolation and Southern blot

For genomic DNA isolation, an overnight 5 ml-culture of cells was pelleted and resuspended in 300 µl lysis buffer (100 mM Tris pH8.0, 50 mM EDTA, 250 mM NaCl and 1% SDS). The equivalent of 200 µl of acid-washed glass beads was added to the samples and cells were mechanically lysed by 15 cycles of 1 min vigorous vortex and 1 min incubation on ice. Cell lysates were recovered and transferred to fresh microtubes. DNA extraction was done using a phenol-chloroform procedure (89). 500 ng of XhoI-digested genomic DNA was migrated on a 0.8% TBE agarose gel and transferred onto an Amersham Hybond-N+ membrane (Cytiva). After transfer, DNA was cross-linked to the membrane using UV exposure (254 nm for 60 seconds) in a SpectroLinker apparatus (XL-1000 UV Crosslinker, Spectronics Corporation). Membranes were hybridized with ^32^P-dCTP-labeled pCT300 and CENIV probes. The pCT300 probe, a 300-base-pair (bp) fragment containing 280 bp TG repeats derived from pYLPV (90), was used to detect telomere restriction fragments (TRFs). The CEN IV probe, which detects a band at ~1.5 Kb, was used as a loading control. Probes were amplified by PCR, followed by random priming labeling procedure (91). Blots were exposed in a phosphor imaging cassette for 2 to 3 days and were visualized using a Personal Molecular Imager apparatus (Bio-Rad). Southern blot experiments of Est2, Est3, Est1, and Pop1 mutants were all performed with three biological replicates.

### Quantification of telomere length by Southern blot

Telomere restriction fragment (TRF) lengths were quantified using GelAnalyzer 23.1 software. Southern blot images were calibrated using the GeneRuler 1 kb+ DNA Ladder to assign a molecular weight (in base pairs) to each band. Bands corresponding to CEN IV (at ~1,5 Kb) and TRFs were individually detected in each lane, and the software identified the peak intensity of their signals. TRF migration in each lane was normalized by subtracting the TRF band from the CEN IV band. Relative telomere shortening was calculated by subtracting the measured differences between CEN IV and TRFs (in base pairs) of each mutant strain from the WT strain.

### Protein extractions

For the validation of the expression of the HA_3_-Pop1, Est1-myc and FLAG-Est3 constructs, 50 ml of exponential growing cell cultures was pelleted and resuspended in lysis buffer (1X PBS, 0.05% IGEPAL CA-630 and 1X Protease inhibitors cocktail). An equivalent of 200 µl of acid-washed glass beads was added to the sample, and cells were mechanically lysed by 15 cycles of 1 min vigorous vortex and 1 min incubation on ice. The resulting cell extracts were spun in a centrifuge for 15 min at max speed and the supernatant was transferred to a fresh tube. Protein concentration was determined by Bradford assay.

### Immunoprecipitations of FLAG-Est2

For the validation of the expression of the FLAG-Est2 in the *est2Δ* strain, total protein extracts and immunoprecipitations were prepared and followed by Western blot analysis. Briefly, 50 ml of exponential growing cell cultures was pelleted and resuspended in buffer 1 (10 mM Tris-HCl pH8.0, 1 mM MgCl_2_, 10% glycerol, 200 mM NaCl, 0.2% Triton X-100, 0.2% IGEPAL CA-630, 100 mM DTT and 1X Protease inhibitors cocktail). An equivalent of 200 µl of acid-washed glass beads was added to the samples and cells were mechanically lysed by 15 cycles of 1 min vigorous vortex and 1 min incubation on ice. The supernatant was transferred to a new tube following a 15 min centrifugation at max speed and the protein concentration was measured using a Bradford assay.

For the immunoprecipitations of FLAG-Est2, 3 mg of total proteins was mixed with 1 µg of anti-FLAG M2 antibody (Sigma-Aldrich, Cat# F1804, Lot SLBQ6349V) and incubated at 4°C, with rotation, overnight. An input fraction was harvested before adding the antibody. The next day, 50 µl of protein-G-coupled Dynabeads (Invitrogen, Cat# 10004D) was added to the samples and further incubated at 4°C, for 3 h. Beads were washed twice with buffer 1 and twice with buffer 2 (10 mM Tris-HCl pH8.0, 1 mM MgCl_2_, 10% glycerol, 200 mM NaCl, 100 mM DTT and 1X Protease inhibitors cocktail). After the washes, the beads were resuspended in buffer 2 and mixed with 2X Laemmli buffer for Western blot analysis.

### Immunoblotting for validation of the expression of HA_3_-Pop1, Est1-myc, FLAG-Est3 and FLAG Est2

Protein extracts and immunoprecipitation fractions were mixed with 2X Laemmli buffer and migrated on 10% SDS-PAGE gels (TGX Stain-Free FastCast Acrylamide Kit, 10%, Bio-Rad, Cat# 1610183). Proteins were transferred onto a 0.45 µm nitrocellulose membrane (Bio-Rad, Cat# 1620115). Membranes were blocked in 5% milk in 1X Tris-buffered saline (TBS) supplemented with 0.1% Tween-20 for 30 min. All subsequent incubation with primary and secondary antibodies was performed in TBST with 1% milk. For analyses of HA_3_-Pop1, the membrane was incubated overnight with mouse anti-HA.11 antibody (Covance, Cat# MMS-101P, Lot 14862002, 1:1000 dilution). For the detection of FLAG-Est2 and FLAG-Est3, membranes were incubated with a mouse anti-FLAG M2 antibody (Sigma-Aldrich, Cat# F1804, Lot SLBQ6349V, 1:1000 dilution). For Est1-myc, the membrane was incubated with mouse anti-c-myc antibody (Roche, clone 9E10, Cat# 11667149001, Lot 77023900, 1:500 dilution). As a loading control, membranes were incubated rabbit anti-α-tubulin antibody (Abcam, Cat# ab184970, Lot GR3316025-3, 1:10,000 dilution). Membranes were incubated 1 h at room temperature with secondary antibody ECL sheep anti-mouse IgG HRP-conjugated (Cytiva, Cat# NA931V, lot 17358735, 1:2000 dilution) for HA_3_-Pop1, FLAG-Est2, FLAG-Est3 and Est1-myc analyses. For the detection of α-tubulin, the secondary antibody ECL goat anti-rabbit IgG HRP-conjugated (Bio-Rad, Cat# 170-6515, Lot L005679A, 1:5000 dilution). After reaction of HRP with Clarity Western ECL Substrate (Bio-Rad, Cat# 170-5061) for the detection of HA_3_-Pop1 and α-tubulin or with SignalFire™ ECL Reagents (Cell Signaling Technology, Cat# 6883P3) for FLAG-Est2, FLAG-Est3 and Est1-myc, blots were visualized with a ChemiDoc MP Imaging System (Bio-Rad).

### Negative staining EM

For negative staining EM sample preparation, a holey carbon copper grid with continuous carbon layer (Zhongjingkeyi Technology Co. Ltd, Cat# BZ31023b) was first glow-discharged for 15 s at 30 mA with a Sputter Coater discharger (Edwards S150B). 3 μl of dialyzed yeast telomerase holoenzyme complex was applied onto the discharged grid and incubated for 90 s. The grid was then stained with 2% (w/v) uranyl formate for a total of 1 min. The stain was then blotted away, and the grid was air-dried at room temperature. For data collection, we used a 200 kV F20 Technai transmission electron microscope equipped with a Falcon II direct electron detector. A dataset of 620 micrographs was collected using a physical pixel size of 3.58 Å and a total dose of 50 e^-^/Å^2^.

Negative staining data processing pipeline was shown in Supplementary Figure 2. Data processing was done in RELION 5.0 (92, 93). Contrast transfer function (CTF) parameters were estimated using CTFFIND 4.1 within RELION (94). Particle picking was first done using RELION LoG picker (95) to get 2D class references. Reference-base picking was carried out and picked a total of 148,346 particles. Particles were extracted and were used to generate a 3D initial reference in RELION. 3D classification was then done with 10 classes and 25 iterations. A class with 28,217 particles was selected and refined to 14.3 Å resolution (fig. S2).

### Cryo-EM sample preparation and data collection

For cryo-EM sample preparation using UltrAufoil R1.2/1.3 300 mesh grids (Quantifoil) coated with graphene oxide, following a published protocol (96). Before use, grids were glow discharged for 2 s at 30 mA using a Sputter Coater discharger (Edwards S150B). 3 μl of dialyzed yeast telomerase holoenzyme complex was applied onto a grid and incubated for 5 min at 4 °C. Sample was blotted away using Whatman blotting paper (Whatman, Cat# WHA1001813) with a blot time of 7.0 s, a blot force of 10. The grid was then vitrified by plunge-freezing in liquid ethane at 4 °C and 100% humidity using an Vitrobot MK IV (Thermo Fisher Scientific).

For data collection, grids were loaded onto a Thermo Fisher Scientific Titan Krios transmission electron microscope operating at 300 kV with a Falcon 4i direct electron detector and a Selectris X energy-filter. Data collection was carried out using EPU software (Thermo Fisher Scientific). A total of 24,936 movies was recorded at 130,000x magnification with a physical pixel size of 0.955 Å/pixel. For each movie, a total electron dose of 50 e^-^/Å^2^ was used over an exposure time of 4.4 s. Data were collected with a defocus range of -1 to -2.4 μm.

### Cryo-EM image processing

Cryo-EM data processing pipeline was shown in fig. S3. After cryo-EM data collection, motion correction was performed in RELION 5.0 (92, 93), and CTF parameters was estimated using CTFFIND 4.1 incorporated in RELION (94). Particle picking was done using Topaz (97), yielding 1,876,851 picked particles. Particles were then extracted and subjected to one round of 2D classification. Particles in good 2D classes were imported into CryoSPARC for heterogenous refinement (98). 372,930 particles were selected for 2D classification. A subset of 299,185 particles from good 2D classes was used for non-uniform refinement (99). The refinement yields a 3.0 Å yeast telomerase overall map.

To further improve the resolution of the catalytic core, we performed particle subtraction to keep only the catalytic core. The resulting particles were used for local refinement, which yielded a map for the yeast telomerase catalytic core at 3.7 Å resolution.

3D variability analysis (3DVA) was carried out for the yeast telomerase consensus structure and the catalytic core (fig. S3) (100). Resolutions of maps were determined using the gold-standard Fourier Shell Correlation (FSC) = 0.143 criterion between the two half-maps resulting from non-uniform refinement or local refinement. Local resolutions were calculated in CryoSPARC. Directional FSC and sphericity were calculated using 3DFSC server (https://3dfsc.salk.edu/). 2D histograms of the Euler angle distribution were generated in CryoSPARC.

### Modelling and refinement

For protein model building, we first performed rigid-body fitting of known protein models into the 3.0 Å consensus cryo-EM map in ChimeraX (101). For Est2 and Est3, Alphafold2 prediction of Est2-Est3 complex was used for initial fitting (102). For the fitting of Pop1/6/7 complex, the cryo-EM structure of yeast ribonuclease P (PDB 6AGB) was used (72). For the initial docking of Est1, crystal structure of *Kluyveromyces lactis* Est1–Cdc13 complex (PDB 5Y5A) was used (25). After fitting, models were subsequently re-built in ISOLDE and COOT (103, 104).

For RNA model building, the P3 domain in the IVc stem was built based on the cryo-EM structure of yeast Ribonuclease P (PDB 6AGB) (72). *K. lactis* telomerase RNA pseudoknot NMR structure (PDB 2M8K) was used for the initial fitting of the pseudoknot region (105). For the STE region, *Candida albicans* TERT-TWJ crystal structure (PDB 6ZDQ) was used for initial fitting (35). Other parts of RNA were built *de novo* in COOT based on RNA secondary structures (104). After fitting, models were then re-built using ISOLDE (103). The geometry of the TLC1 was further improved using ERRASER2.0 (106).

Model refinements were performed using Phenix and Servalcat (107, 108).Models were validated using Molprobity (109) and wwPDB validation system (https://validate-rcsb-1.wwpdb.org/). Model versus map FSC was calculated using Phenix (107).

### Sequence alignment and structural prediction

Protein sequences from different species were obtained from UniProt and NCBI databases. Multiple sequences were performed using T-Coffee (110). The results were used as input to ESPript (111) for illustration.

Structural prediction was performed using Alphafold3 server (47). PAE plot of predicted models were made using a Python script (https://github.com/nayimgr/af3analysis). For the prediction of TERT ZnF motifs, TERT sequences from different eukaryotic species and one or multiple zinc ion(s) were used as input for Alphadold3 structure prediction (Table S3). Results were inspected manually based on zinc ion location and zinc coordination chemistry.

### Map and model visualization

Maps and models were visualized using ChimeraX and Pymol (Schrödinger Inc.). Illustrations were made in Adobe Illustrator (Adobe Inc.).

## Supplementary Material

mdar checklist

Supp Mov1

Supplementary Materials

## Data Availability

The cryo-EM maps of the yeast telomerase holoenzyme and the yeast telomerase catalytic core are deposited with the Electron Microscopy Database under accession codes EMD-55321 and EMD-55322, respectively. The refined atomic models for the yeast telomerase holoenzyme and the yeast telomerase catalytic core are deposited with the Protein Data Bank under accession numbers 9SWN and 9SWO, respectively. Materials are available from T.H.D.N., R.W and P.C. under a material transfer agreement with the MRC Laboratory of Molecular Biology. Correspondence should be addressed to P.C., R. W. and T.H.D.N.

## One-Sentence Summary

Structure of yeast telomerase provides insights into the function and evolution of telomerase holoenzymes
