# Supplementary material for: Cryo-EM structure of the budding yeast telomerase holoenzyme
Source: Science. Author manuscript; Available in PMC 2026 May 7. (PMC7619062; doi:10.1126/science.adz5344)
Supplement: Supplementary Materials [file EMS213641-supplement-Supplementary_Materials.pdf]

Supplementary Materials for

Cryo-EM structure of the budding yeast telomerase holoenzyme

Hongmiao Hu*, Hannah Neumann, Gabriela M. Teplitz, Elsa Franco-Echevarría, Pascal Chartrand*, Raymund J. Wellinger*, Thi Hoang Duong Nguyen*

*Corresponding authors: **p.chartrand@umontreal.ca**, **raymund.wellinger@usherbrooke.ca,hhu@mrc-lmb.cam.ac.uk**, **knguyen@mrc-lmb.cam.ac.uk**

The PDF file includes:

Figs. S1 to S25

Tables S1 to S6

References (*112-118*)

Other Supplementary Materials for this manuscript include the following:

Movies S1

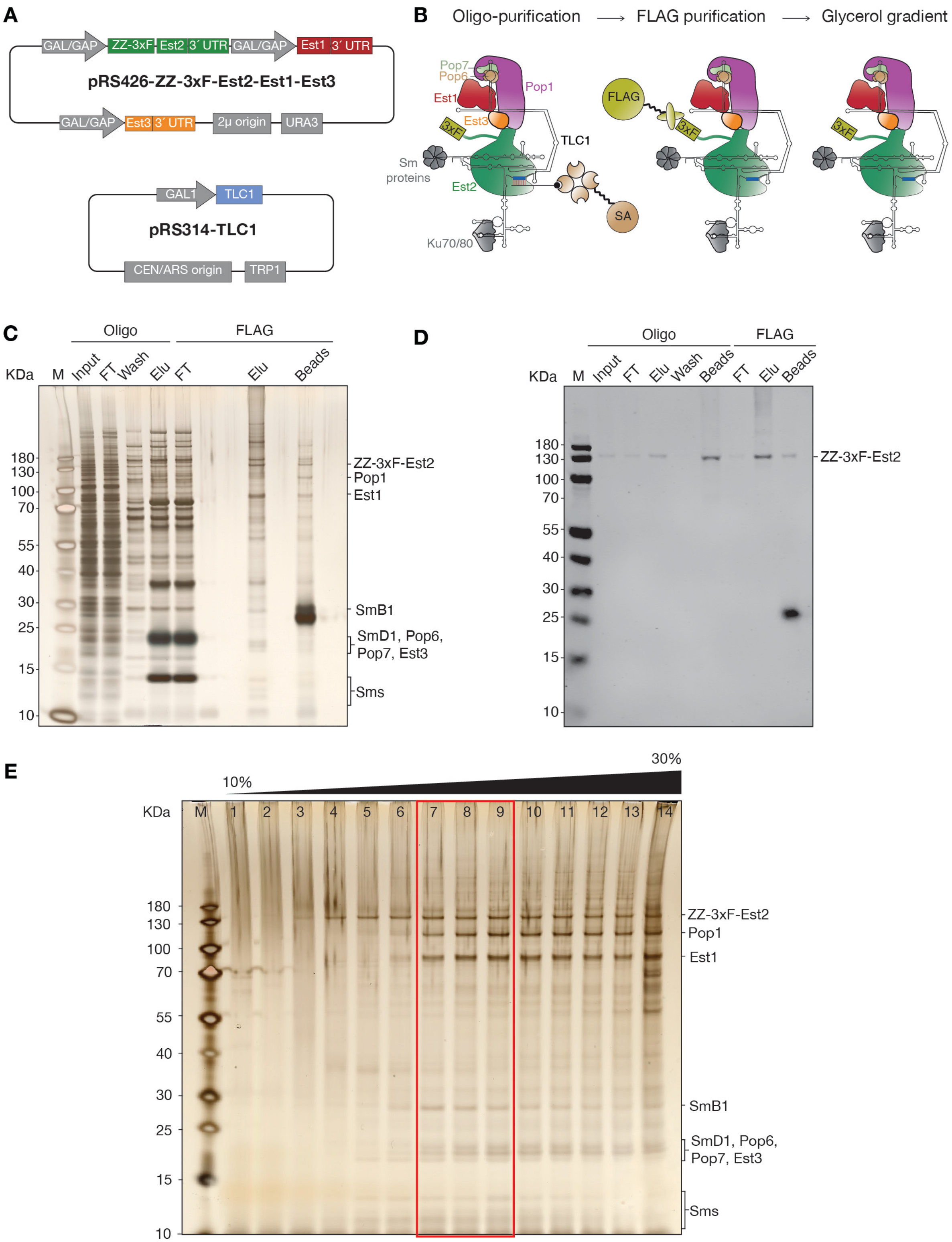
A
GAL/GAP
ZZ-3xF
Est2 3´ UTR
GAL/GAP
Est1 3´ UTR
pRS426-ZZ-3xF-Est2-Est1-Est3
GAL/GAP
Est3 3´ UTR
2μ origin
URA3
GAL1
TLC1
pRS314-TLC1
CEN/ARS origin
TRP1
B
Oligo-purification
FLAG purification
Glycerol gradient
Pop7
Pop6
Pop1
Est1
Est3
3xF
TLC1
Sm proteins
Est2
SA
Ku70/80
FLAG
C
Oligo
FLAG
KDa
M
Input
FT
Wash
Elu
FT
Elu
Beads
ZZ-3xF-Est2
Pop1
Est1
SmB1
SmD1, Pop6, Pop7, Est3
Sms
D
Oligo
FLAG
KDa
M
Input
FT
Elu
Wash
Beads
FT
Elu
Beads
ZZ-3xF-Est2
E
10%
30%
KDa
M
1
2
3
4
5
6
7
8
9
10
11
12
13
14
ZZ-3xF-Est2
Pop1
Est1
SmB1
SmD1, Pop6, Pop7, Est3
Sms

Fig. S1. Biochemical reconstitution and purification of the yeast telomerase holoenzyme. (**A**) Schematic of plasmids used for overexpression of Est1, Est2, Est3 and TLC1. (**B**) Schematic of the yeast telomerase purification. (**C**) Silver-stained SDS-PAGE of samples collected during the oligo and FLAG purification steps. (**D**) Immunoblot of samples collected during oligo and FLAG purification steps. An antibody against the FLAG tag was used. (**E**) Silver-stained SDS-PAGE of fractions from the glycerol gradient. The red box indicates the fractions used for cryo-EM.

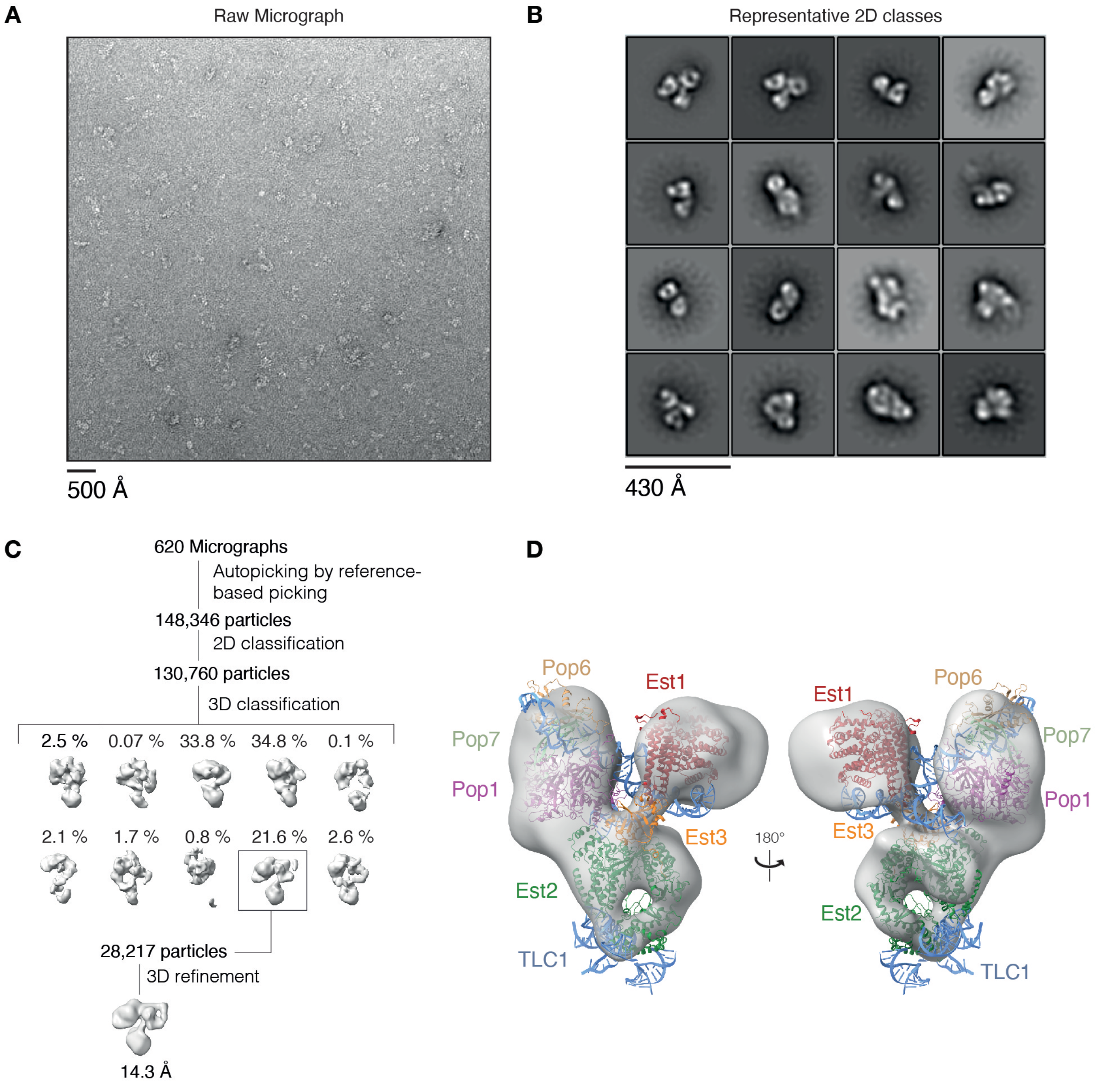

5 **Fig. S2. Negative staining EM analysis of the yeast telomerase holoenzyme.** (**A**) Representative negative staining electron micrograph. (**B**) Representative negative stain 2D averages of the purified yeast telomerase holoenzyme. (**C**) Data processing schematic of the negative staining EM dataset. (**D**) Negative staining EM reconstruction of the yeast telomerase holoenzyme.

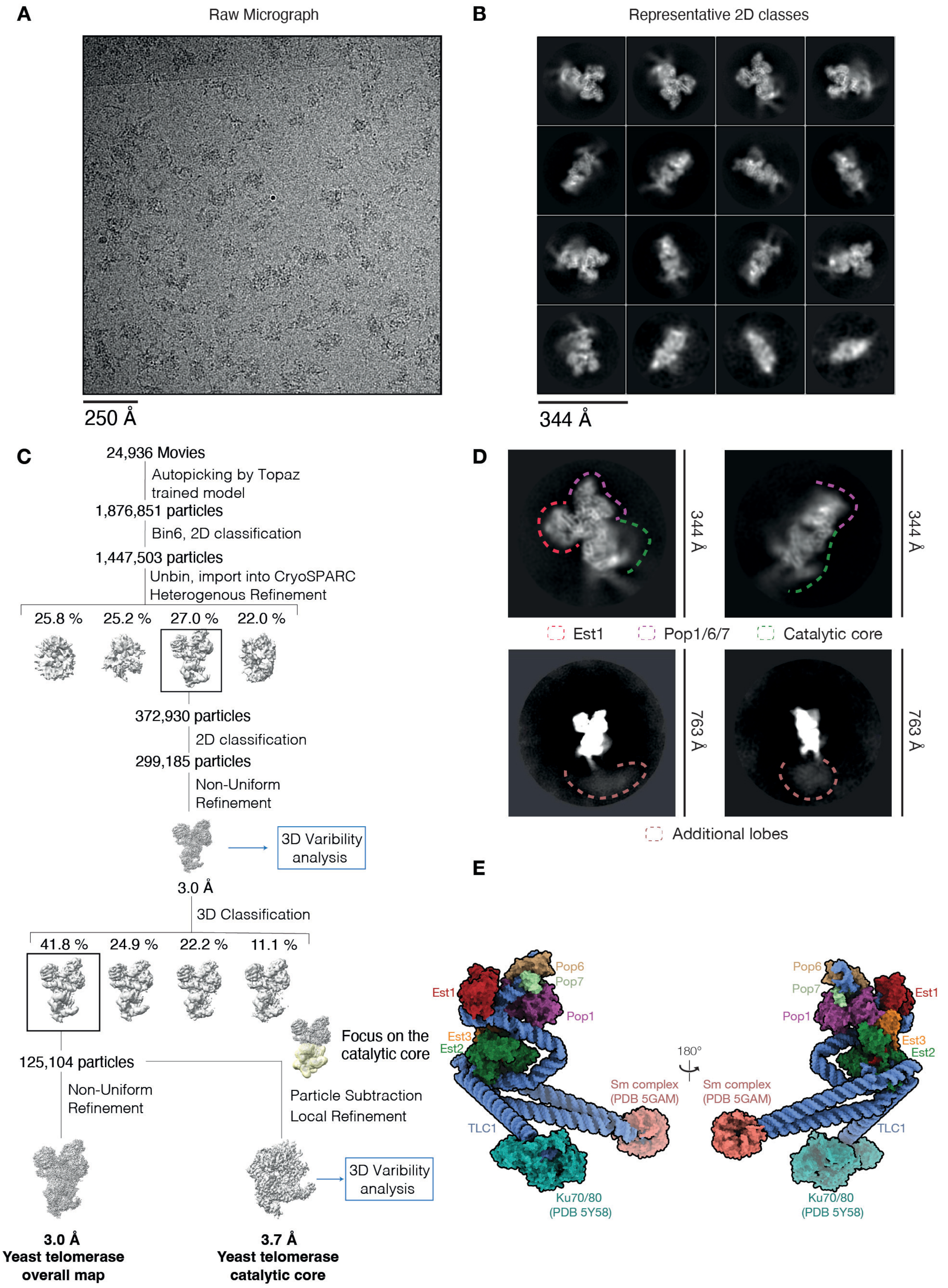
A
Raw Micrograph
250 Å
B
Representative 2D classes
344 Å
C
24,936 Movies
Autopicking by Topaz trained model
1,876,851 particles
Bin6, 2D classification
1,447,503 particles
Unbin, import into CryoSPARC
Heterogenous Refinement
25.8 %
25.2 %
27.0 %
22.0 %
372,930 particles
2D classification
299,185 particles
Non-Uniform Refinement
3D Varibility analysis
3.0 Å
3D Classification
41.8 %
24.9 %
22.2 %
11.1 %
125,104 particles
Non-Uniform Refinement
Focus on the catalytic core
Particle Subtraction
Local Refinement
3D Varibility analysis
3.0 Å
Yeast telomerase overall map
3.7 Å
Yeast telomerase catalytic core
D
344 Å
344 Å
Est1
Pop1/6/7
Catalytic core
763 Å
763 Å
Additional lobes
E
Pop6
Pop7
Est1
Pop1
Est3
Est2
Sm complex (PDB 5GAM)
TLC1
Ku70/80 (PDB 5Y58)
180°
Pop6
Pop7
Est1
Pop1
Est3
Est2
Sm complex (PDB 5GAM)
TLC1
Ku70/80 (PDB 5Y58)

Fig. S3. Cryo-EM data processing of the yeast telomerase holoenzyme. (**A**) Representative cryo-EM image. (**B**) Representative cryo-EM 2D averages of the yeast telomerase holoenzyme. (**C**) Cryo-EM data processing strategy for the yeast telomerase holoenzyme. (**D**) 2D class averages of the yeast telomerase holoenzyme with two different box sizes, as indicated. The upper panel shows the locations of Est1, Pop1/6/7 and the catalytic core. The lower panel shows 2D class averages with a bigger box size. Flexible parts indicated are likely the Sm_7 and Ku complex. (**E**) A model of the yeast telomerase holoenzyme. In this model, we combined the atomic model built into the cryo-EM density shown in Fig. 1E with published structures of the yeast Sm7 and Ku complexes (*24, 25*). The RNA in the Ku arm and the terminal arm is simplified by modelling as perfectly base-paired double-stranded RNA.

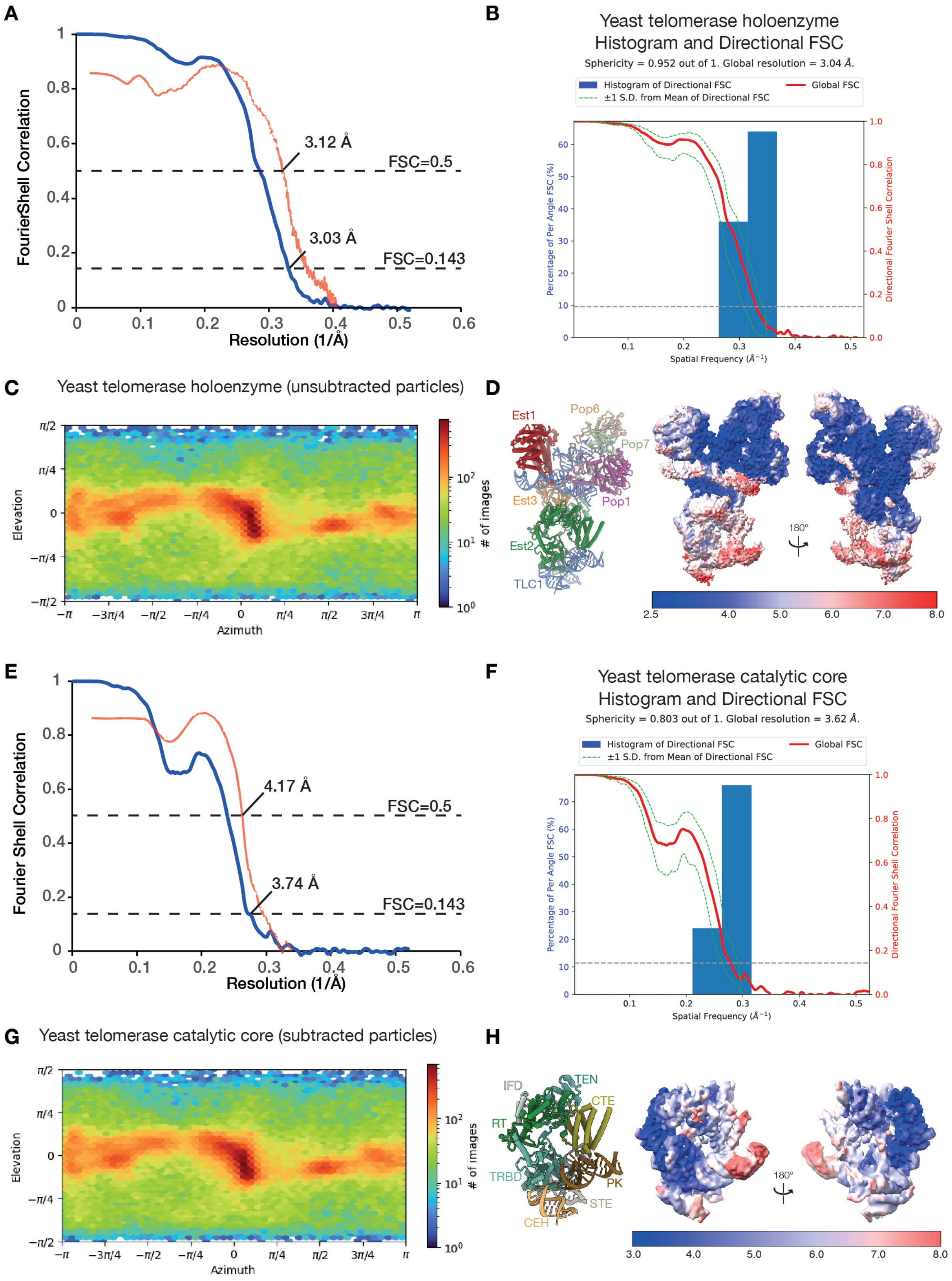
A
FourierShell Correlation
Resolution (1/Å)
3.12 Å
FSC=0.5
3.03 Å
FSC=0.143
B
Yeast telomerase holoenzyme
Histogram and Directional FSC
Sphericity = 0.952 out of 1. Global resolution = 3.04 Å.
Histogram of Directional FSC
±1 S.D. from Mean of Directional FSC
Global FSC
Percentage of Per Angle FSC (%)
Directional Fourier Shell Correlation
Spatial Frequency (Å^{-1})
C
Yeast telomerase holoenzyme (unsubtracted particles)
Elevation
Azimuth
# of images
D
Est1
Pop6
Pop7
Est3
Pop1
Est2
TLC1
180°
E
Fourier Shell Correlation
Resolution (1/Å)
4.17 Å
FSC=0.5
3.74 Å
FSC=0.143
F
Yeast telomerase catalytic core
Histogram and Directional FSC
Sphericity = 0.803 out of 1. Global resolution = 3.62 Å.
Histogram of Directional FSC
±1 S.D. from Mean of Directional FSC
Global FSC
Percentage of Per Angle FSC (%)
Directional Fourier Shell Correlation
Spatial Frequency (Å^{-1})
G
Yeast telomerase catalytic core (subtracted particles)
Elevation
Azimuth
# of images
H
IFD
TEN
CTE
RT
TRBD
PK
STE
CEH
180°

Fig. S4. Overall and local resolution. (**A**) Gold-standard (blue) and model-vs-map (red) FSC plots for the 3.0 Å yeast telomerase consensus map. (**B**) Directional FSC plots and sphericity value for the 3.0 Å yeast telomerase consensus map. (**C**) Euler angle distribution of the particles in the final reconstruction for the 3.0 Å yeast telomerase consensus map. (**D**) Local resolution of the 3.0 Å yeast telomerase consensus map. (**E**) Gold-standard (blue) and model-vs-map (red) FSC plots for the 3.7 Å map of the yeast telomerase catalytic core. (**F**) Directional FSC plots and sphericity value for the 3.7 Å map of the yeast telomerase catalytic core. (**G**) Euler angles distribution of the particles of the 3.7 Å map of the yeast telomerase catalytic core. (**H**) Local resolution of the 3.7 Å map of the yeast telomerase catalytic core.

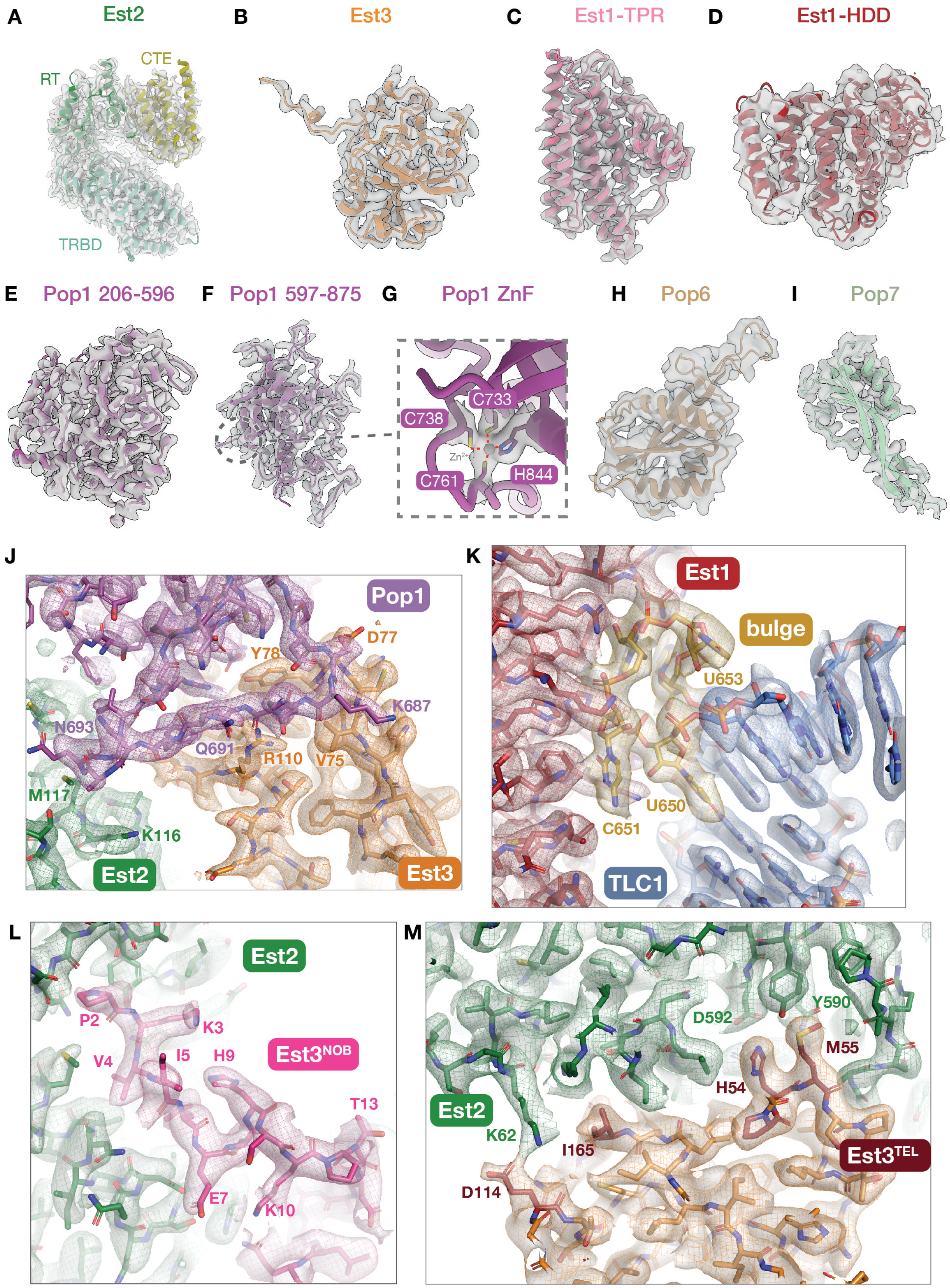
A
Est2
RT
CTE
TRBD
B
Est3
C
Est1-TPR
D
Est1-HDD
E
Pop1 206-596
F
Pop1 597-875
G
Pop1 ZnF
C733
C738
C761
H844
H
Pop6
I
Pop7
J
Pop1
D77
Y78
K687
N693
Q691
R110
V75
M117
K116
Est2
Est3
K
Est1
bulge
U653
U650
C651
TLC1
L
Est2
P2
K3
V4
I5
H9
Est3NOB
T13
E7
K10
M
D592
Y590
M55
H54
Est2
K62
I165
Est3TEL
D114

Fig. S5. Representative densities of the protein components in yeast telomerase. (**A**) Cryo-EM density of Est2 in the catalytic core map. (**B**) Cryo-EM density of Est3 in the consensus map. (**C**) Cryo-EM density of the TPR domain of Est1. (**D**) Cryo-EM density of the HDD domain of Est1. (**E**) Cryo-EM density of residues 206–596 of Pop1. (**F**) Cryo-EM density of residues 597–875 of Pop1. (**G**) Cryo-EM density of the Pop1 ZnF. (**H**) Cryo-EM density of Pop6 in the consensus map. (**I**) Cryo-EM density of Pop7 in the consensus map. (**J**) Cryo-EM density of the Pop1–Est2–Est3 interface. (**K**) Cryo-EM density of the interface between Est1 and the 5-nt bulge on stem IVc of TLC1. (**L**) Cryo-EM density of the Est2–Est3 NOB motif interface. (**M**) Cryo-EM density of the Est2–Est3 TEL patch interface.

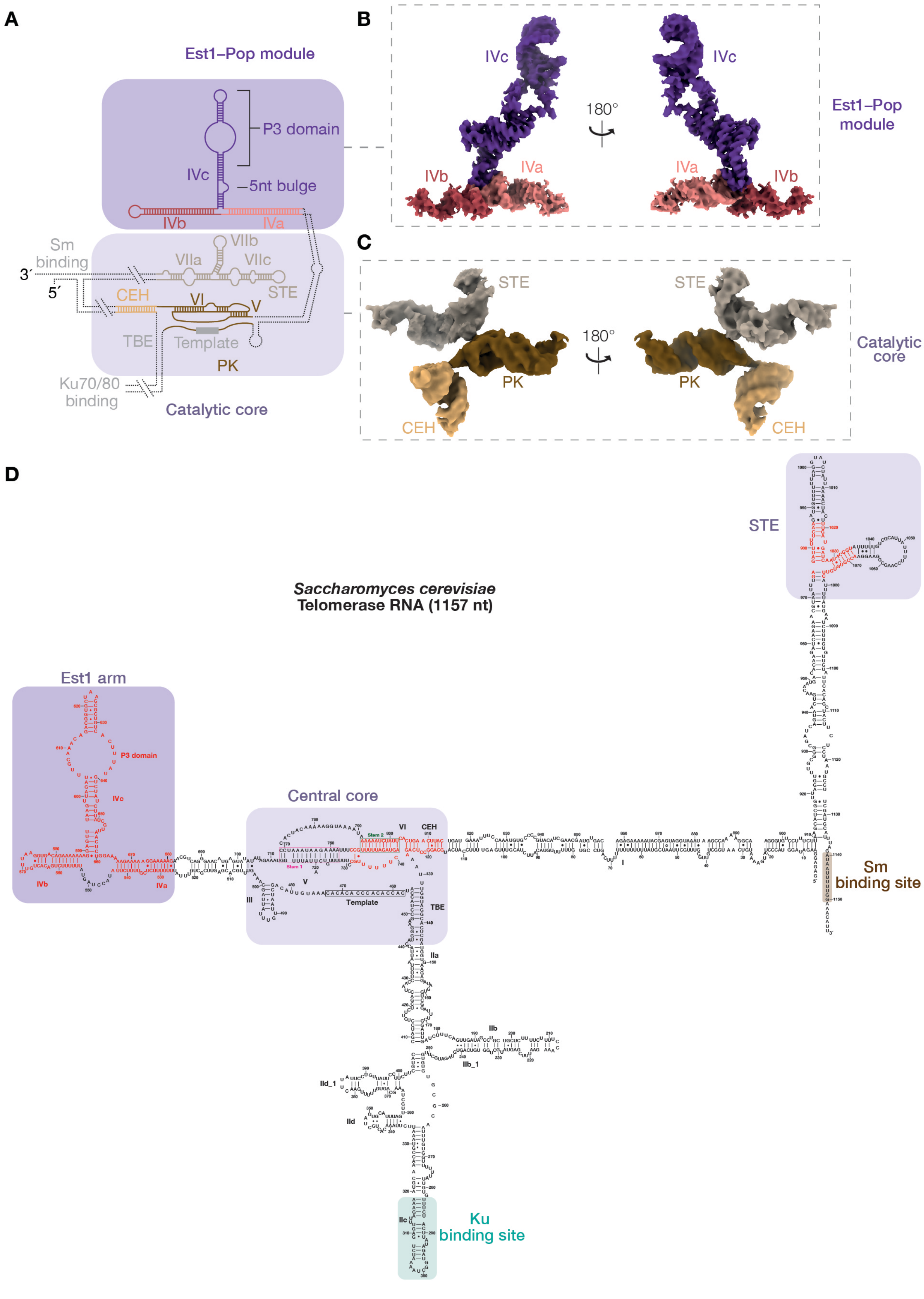
A
Est1–Pop module
P3 domain
IVc
5nt bulge
IVb
IVa
Sm binding
VIIb
VIIa
VIIc
3´
5´
CEH
VI
STE
V
TBE
Template
PK
Ku70/80 binding
Catalytic core
B
IVc
180°
IVb
IVa
Est1–Pop module
C
STE
PK
CEH
Catalytic core
D
STE
Saccharomyces cerevisiae
Telomerase RNA (1157 nt)
Est1 arm
P3 domain
Central core
Stem 2
Stem 1
Template
TBE
Sm binding site
IIa
IIb
IIb_1
IId_1
IId
IIc
Ku binding site

Fig. S6. Representative densities of the RNA component in yeast telomerase. (**A**) Schematic of TLC1 and its domains. (**B**) Cryo-EM density of the TLC1 Est1-arm. (**C**) Cryo-EM density of the central core of TLC1. (**D**) Sequence and secondary structure of TLC1. Nucleotides modeled in the structure are colored in red. PK, pseudoknot; CEH, core-enclosing helix; STE, stem-terminus element.

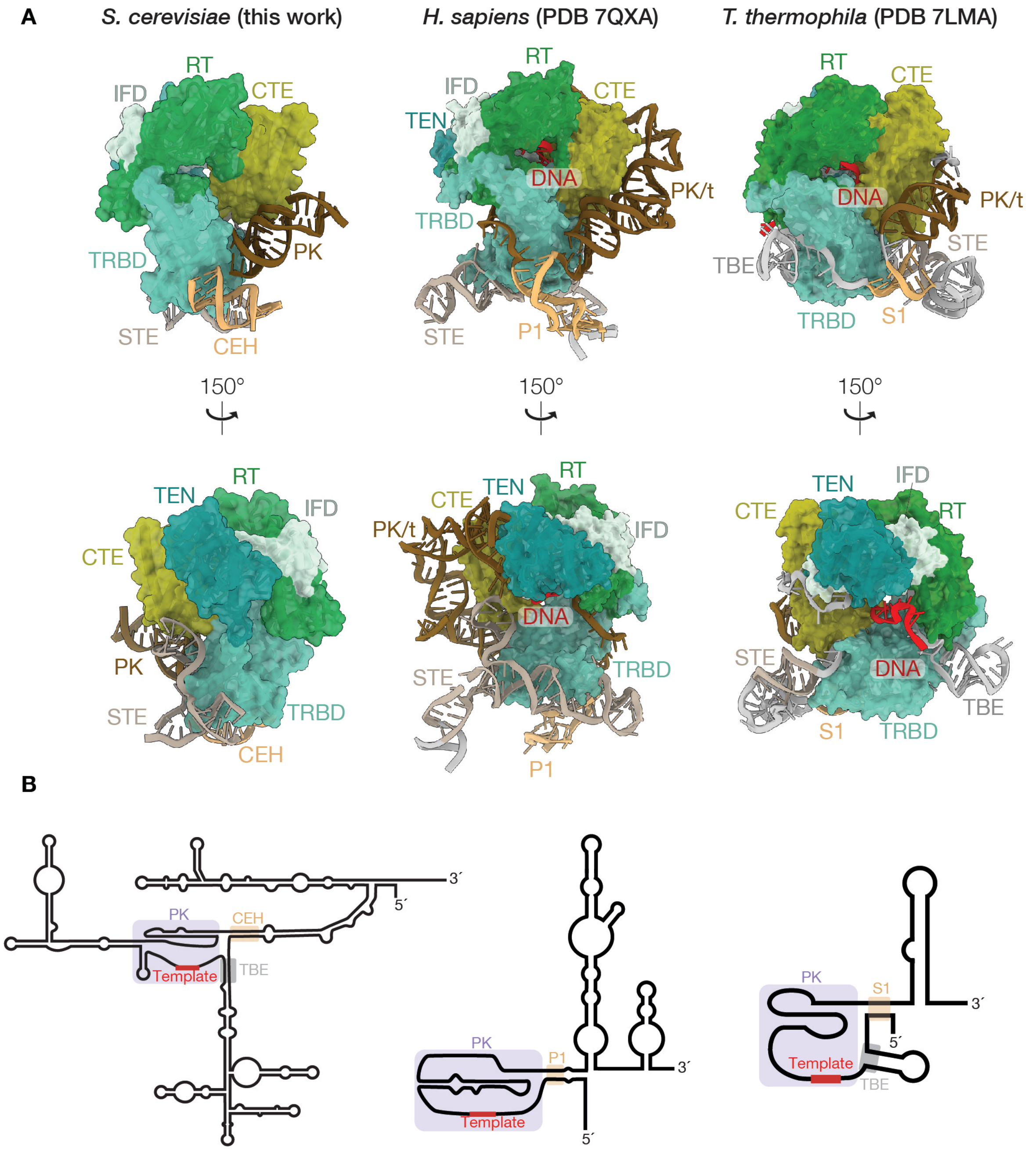

Fig. S7. Comparison of the structure of the catalytic core of yeast, human and *Tetrahymena* telomerase holoenzymes. (**A**) Structures of Est2/TERT bound to domains of TR (*15, 27*). The domains of TERT and TR are colored as shown in Fig. 2A. Structural comparison suggests that the CEH is analogous to human P1 stem and *Tetrahymena* stem 1 (S1). (**B**) Secondary structure schematics of yeast, human and *Tetrahymena* TRs. RNA motifs bound to TERT in the telomerase holoenzyme structures are labelled.

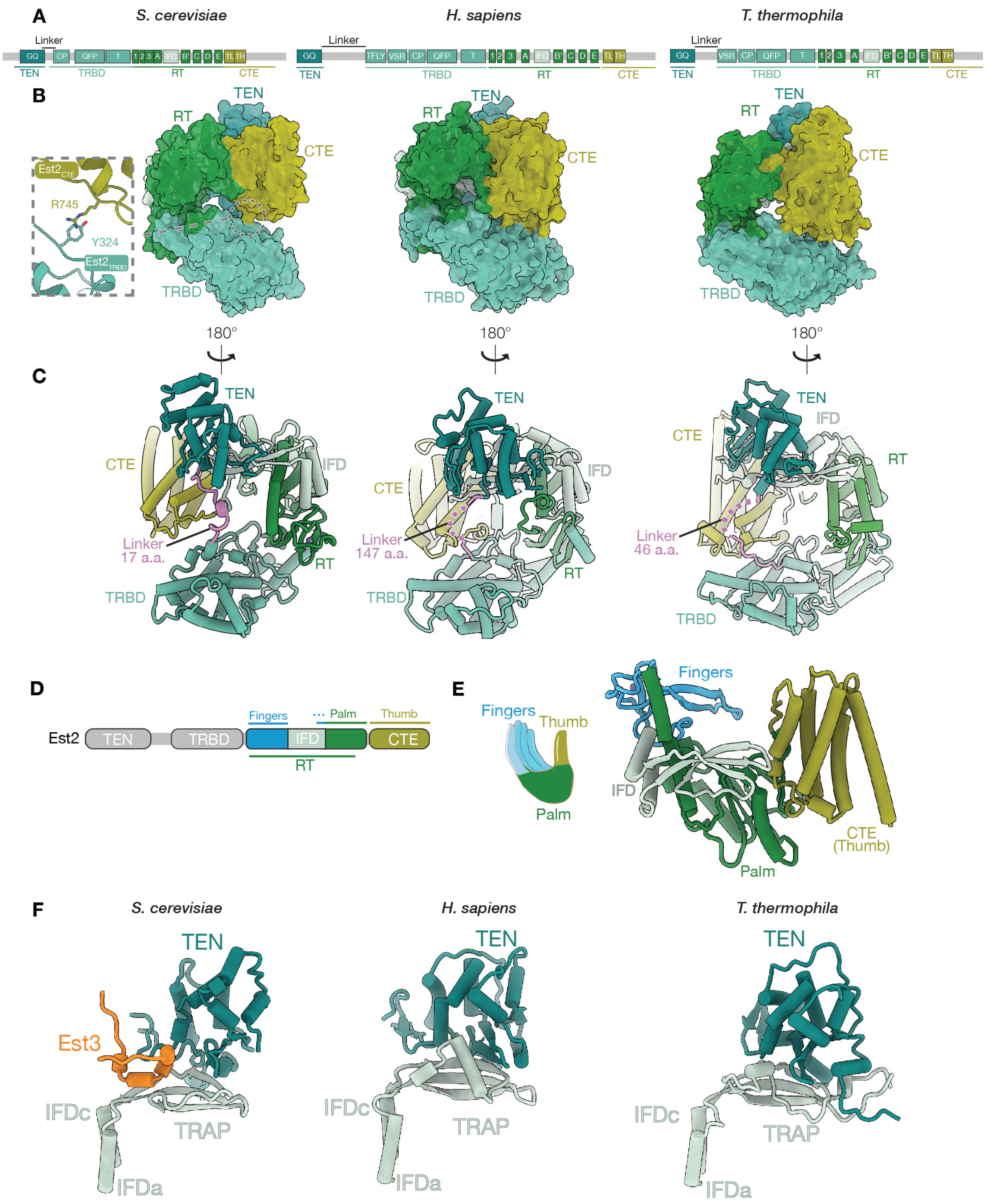

Fig. S8. Comparison of Est2/TERT in yeast, human and *Tetrahymena* telomerase. (**A**) Domain architecture of Est2/TERT from the selected three species whose telomerase structures are available. Conserved motifs important for TERT catalytic activity are shown. (**B**) Structures of Est2/TERT in a space-filling presentation from the selected three species. In the left panel, the inset represents a close-up view of the interaction between the TRBD and CTE of Est2. (**C**)

Structures of Est2/TERT in a cartoon presentation from the selected three species. The linker between the TEN domain and TRBD is indicated. (**D**) Domain schematic of Est2 highlighting regions that form the fingers, palm and thumb motifs in the canonical DNA polymerase right-hand configuration within Est2. (**E**) Structure of the DNA polymerase fingers, palm and thumb motifs of Est2. (**F**) Structures of the TEN domain and the IFD of Est2/TERT in a cartoon presentation from the selected three species. In the left panel, Est3 is also shown.

Est2 sequence alignment

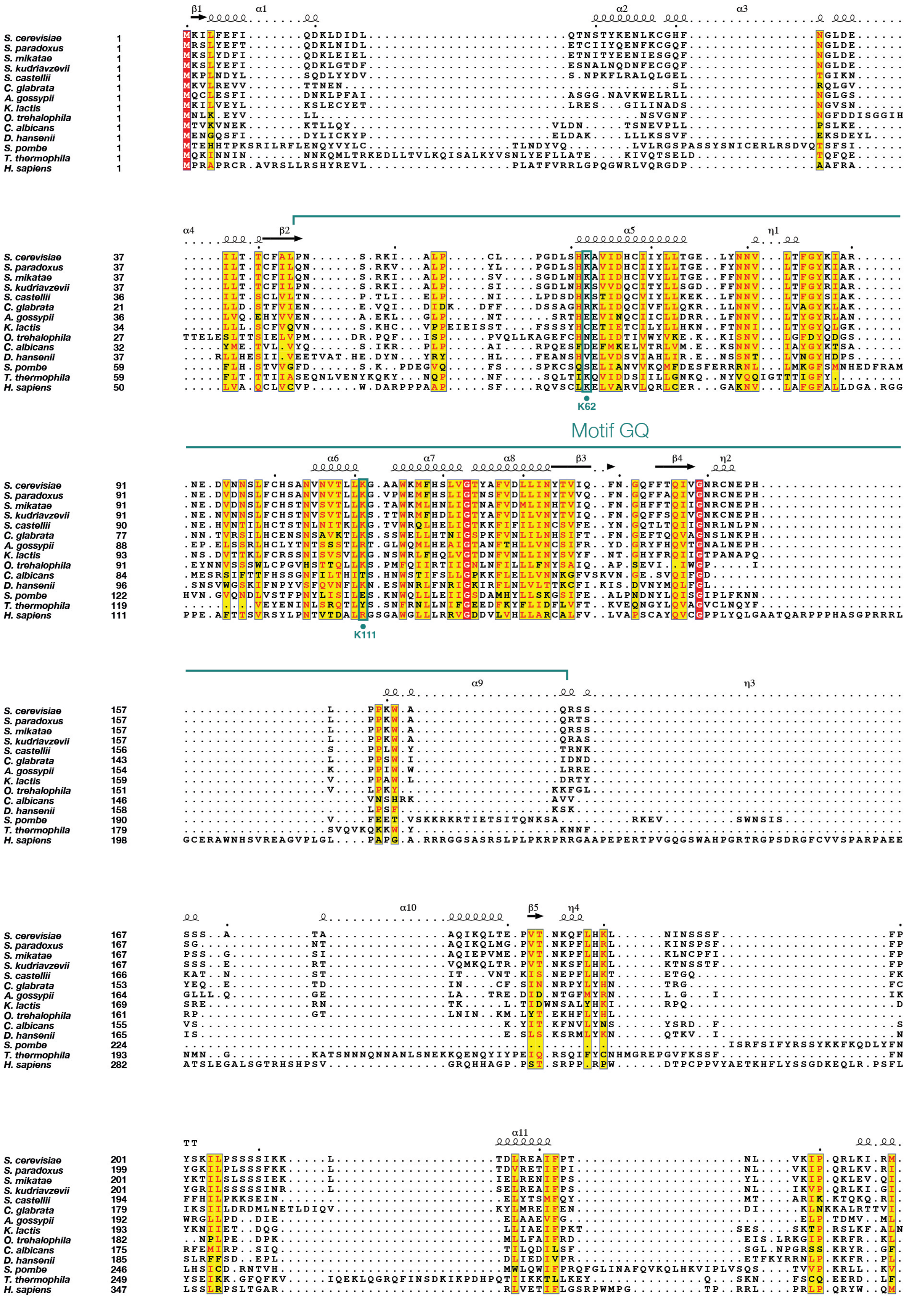

Est2 sequence alignment (continued)

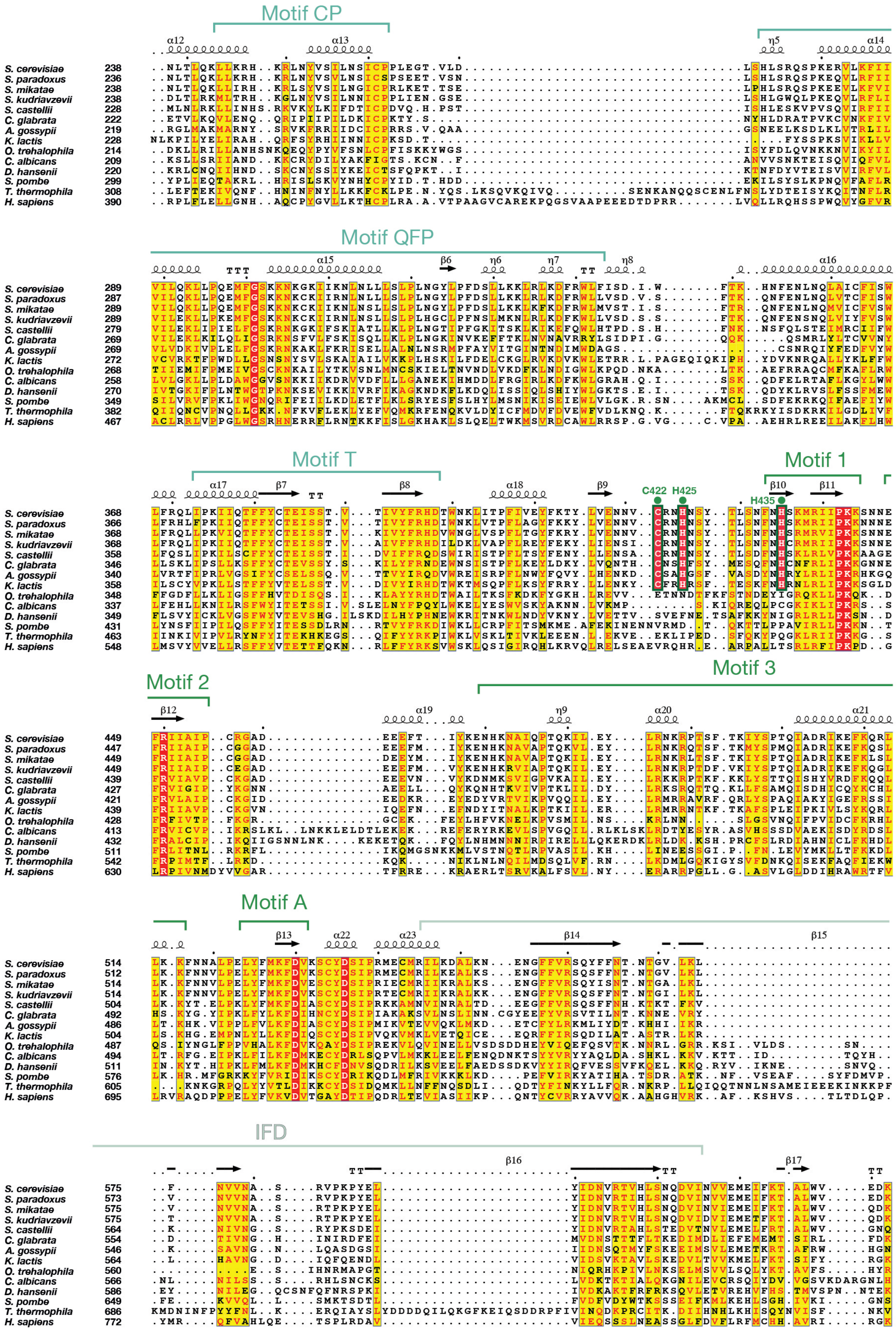

Est2 sequence alignment (continued)

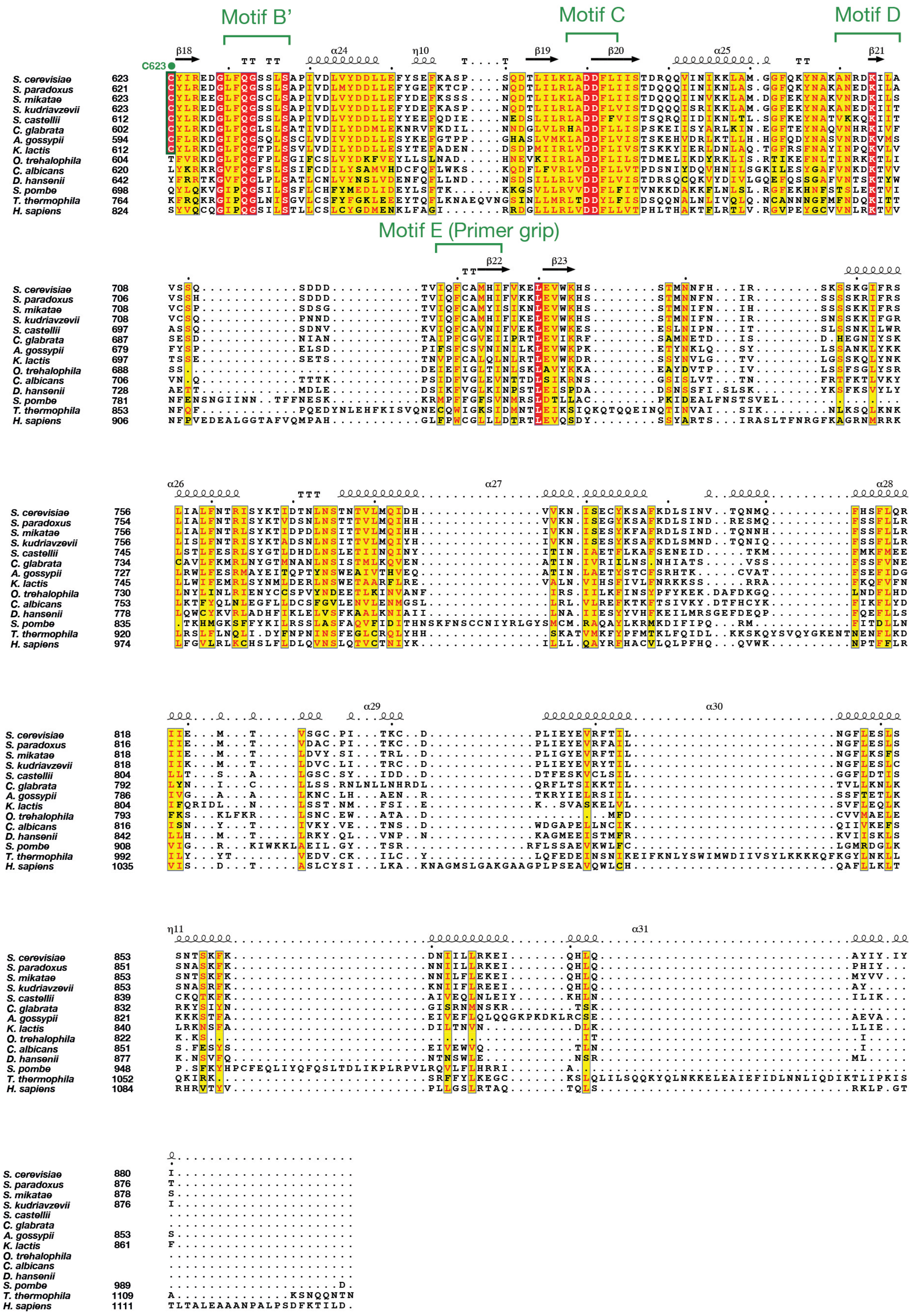

Fig. S9. Sequence alignments of Est2/TERT. Conserved motifs important for telomerase activity are indicated.

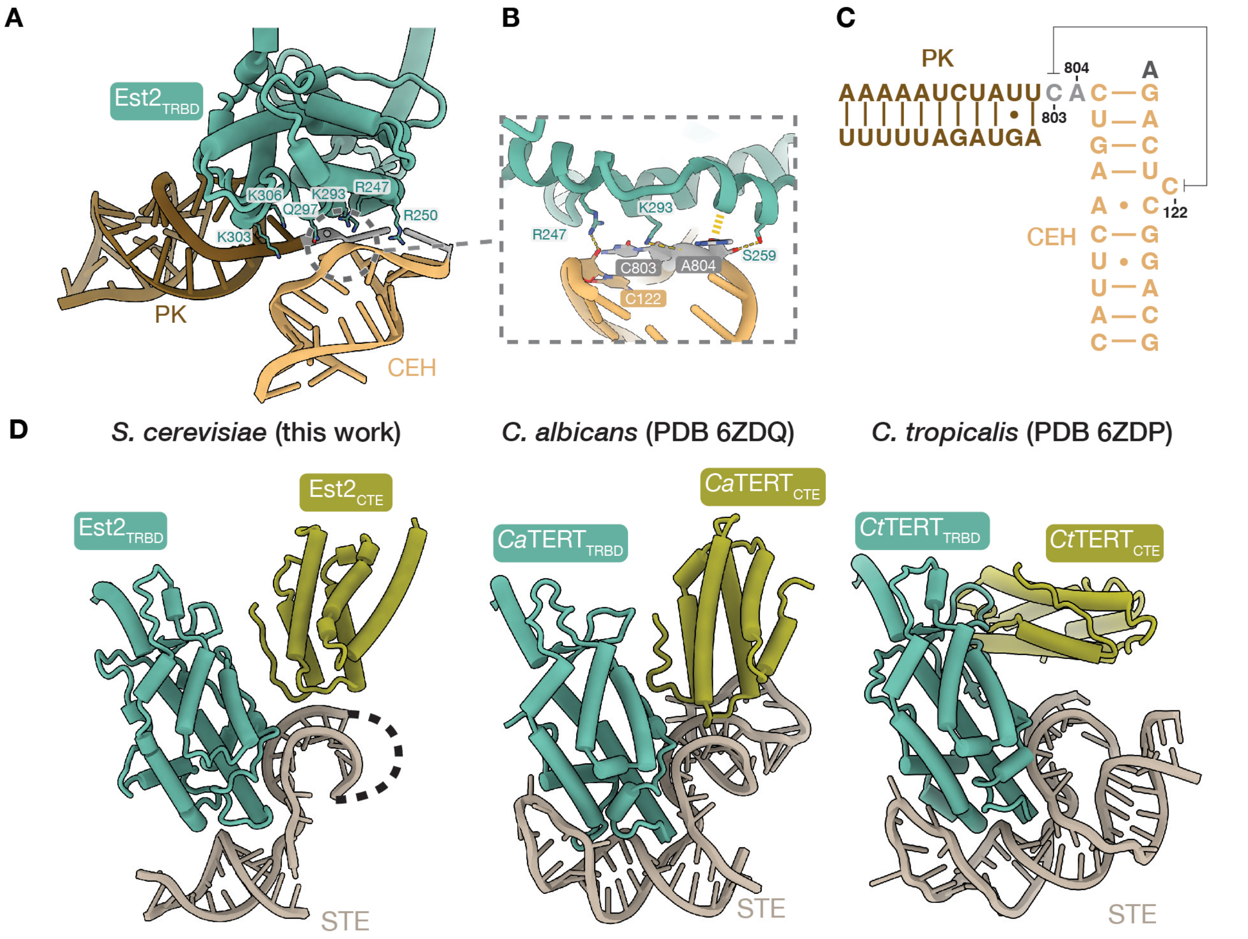

Fig. S10. RNA recognition by Est2 in the yeast catalytic core. (**A**) Est2 TRBD interaction with the CEH and the 2-nt junction between the pseudoknot (PK) and the CEH. (**B**) Inset showing a close-up view of the interaction shown in **A**. (**C**) Sequence and secondary structure of part of the PK, CEH and the junction between them. C803 of the junction and C122 of the CEH form a base stacking interaction with each other. (**D**) Comparison of the Est2 TRBD and CTE bound to the STE of TLC1 with those of *C. albicans* and *C. tropicalis* (*35*).

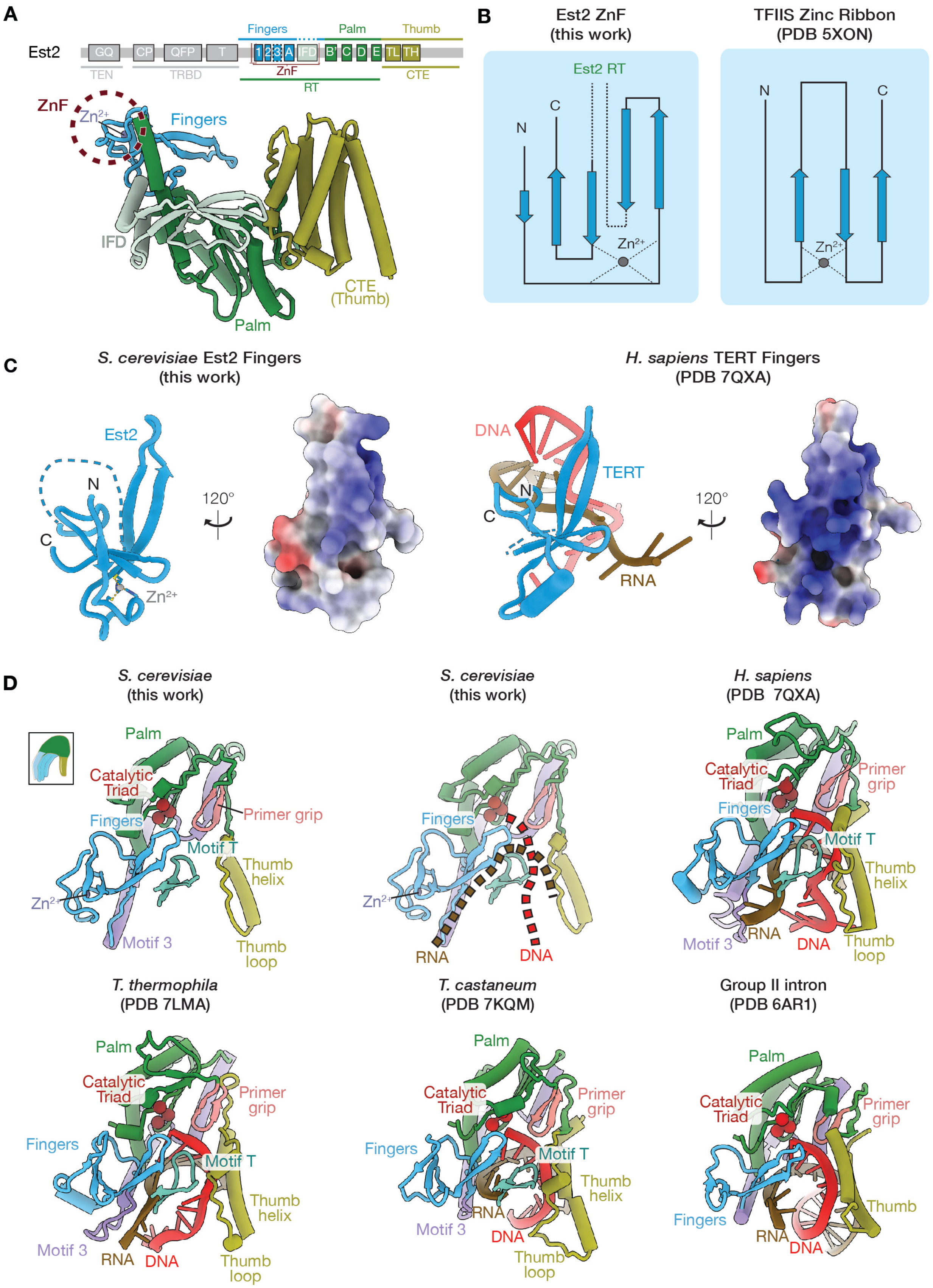
A
Est2
GQ
CP
QFP
T
TEN
TRBD
Fingers
Palm
Thumb
1
2
3
A
IFD
B'
C
D
E
TL
TH
ZnF
RT
CTE
ZnF
Zn2+
Fingers
IFD
CTE
(Thumb)
Palm
B
Est2 ZnF
(this work)
TFIIS Zinc Ribbon
(PDB 5XON)
Est2 RT
N
C
Zn2+
N
C
Zn2+
C
S. cerevisiae Est2 Fingers
(this work)
H. sapiens TERT Fingers
(PDB 7QXA)
Est2
N
C
Zn2+
120°
DNA
TERT
N
C
RNA
120°
D
S. cerevisiae
(this work)
S. cerevisiae
(this work)
H. sapiens
(PDB 7QXA)
Palm
Catalytic
Triad
Primer grip
Fingers
Motif T
Thumb
helix
Zn2+
Motif 3
Thumb
loop
Fingers
Zn2+
RNA
DNA
Palm
Catalytic
Triad
Primer
grip
Fingers
Motif T
Thumb
helix
Motif 3
RNA
DNA
Thumb
loop
T. thermophila
(PDB 7LMA)
T. castaneum
(PDB 7KQM)
Group II intron
(PDB 6AR1)
Palm
Catalytic
Triad
Primer
grip
Fingers
Motif T
Thumb
helix
Motif 3
RNA
DNA
Thumb
loop
Palm
Catalytic
Triad
Primer
grip
Fingers
Motif T
Thumb
helix
Motif 3
RNA
DNA
Thumb
loop
Palm
Catalytic
Triad
Primer
grip
Fingers
Thumb
RNA
DNA

Fig. S11. Conserved motifs in TERT and the ZnF motif in Est2. (**A**) Domain architecture (top) and structure of the conserved polymerase palm, fingers and thumb of Est2 (bottom). The ZnF motif in Est2 is also indicated. (**B**) Topological diagrams of the ZnF of Est2 and that of transcription factor II S (*112*). (**C**) Comparison of the fingers domain of Est2 and human TERT. The right panel shows the electrostatic potential of the fingers domain of Est2 and human TERT. In the human telomerase structure, the fingers domain binds the RNA template (*27*). (**D**) Comparison of conserved motifs in TERT and group II intron RT (*15, 37, 113*).

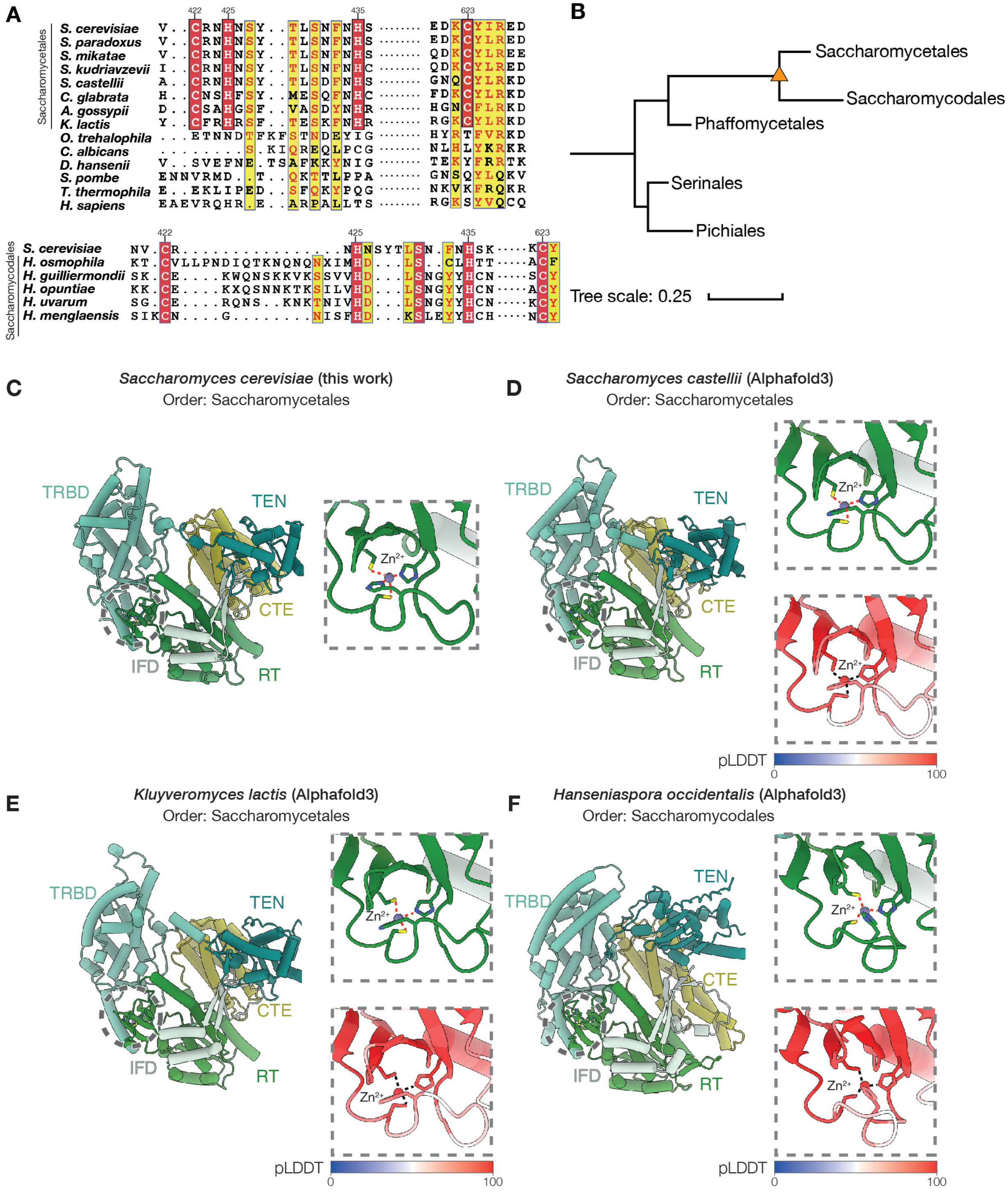

Fig. S12. Conservation of the ZnF motif of Est2 among Saccharomycetales and Saccharomycodales. (**A**) Sequence alignment of the region of Est2/TERT that contains the ZnF motif in Est2. (**B**) Phylogenetic tree of representative yeast orders. The orange triangle indicates the last common ancestor of the Saccharomycetales and the Saccharomycodales, which is likely the origin of ZnF motif in the Est2 protein. Data were derived from y1000+ project (*114*). The

distance shows the divergence of genes in one species from its common ancestors. The unit is arbitrary. (**C**) Structure of Est2 determined in this study. The location of the ZnF motif is indicated in the dashed grey circle. Inset shows a close-up view of the ZnF motif. (**D–F**) AlphaFold3 prediction of TERT from representative species in the Saccharomycetales and Saccharomycodales clades. The location of the ZnF motif is indicated in the dashed grey circle. Inset shows a close-up view of the ZnF motif. The predicted local distance difference test (pLDDT) scores, which represents a per-residue measure of local confidence, are also shown for the prediction of each ZnF.

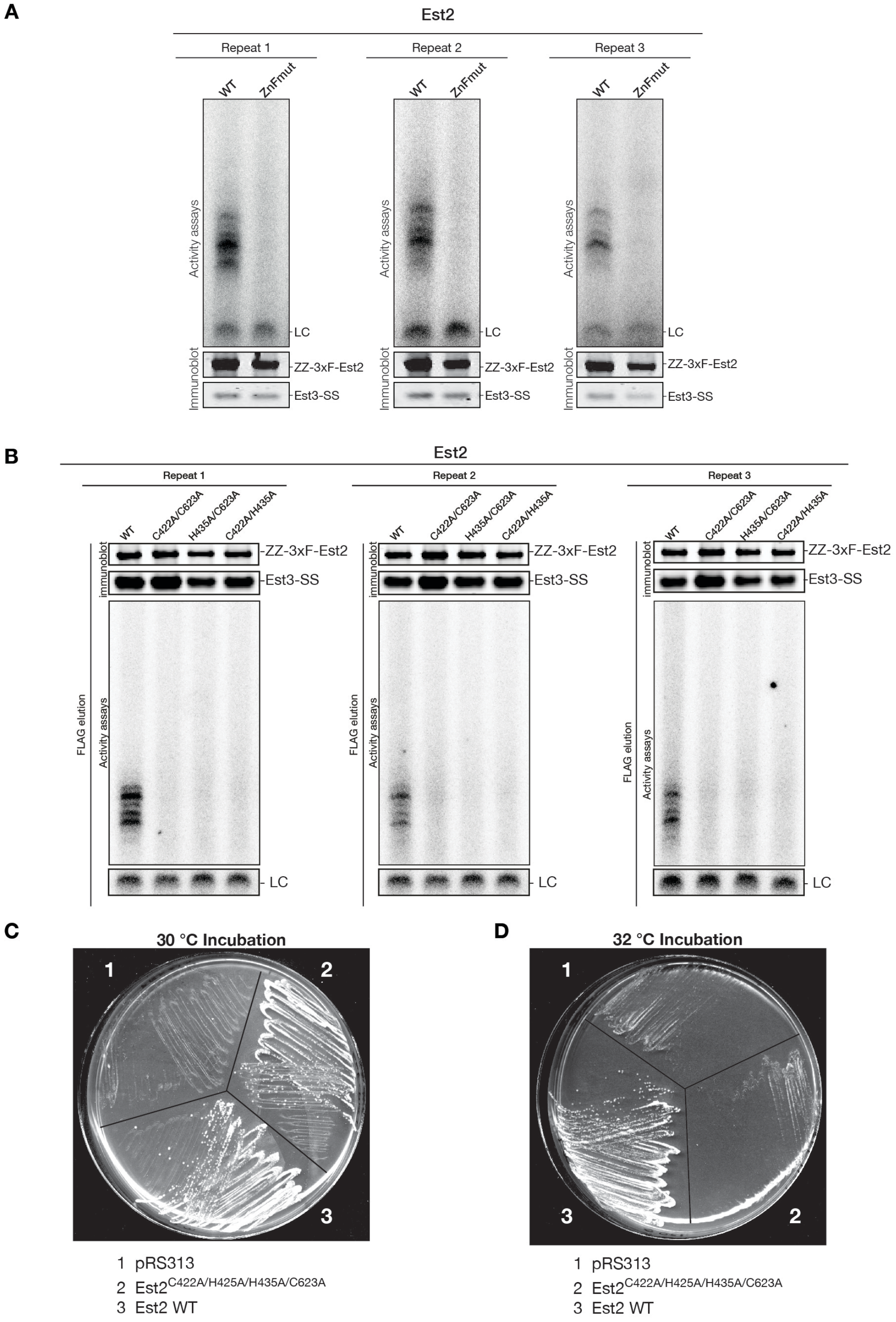

Fig. S13. The Est2 ZnF is critical for telomerase activity *in vitro* and *in vivo*. (**A**) and (**B**) Triplicates of experiments characterizing the effects of disrupting the ZnF motif of Est2 on telomerase activity *in vitro*. Also see Fig. 2D. For these assays, the Est2 ZnF mutant was expressed and purified using the method shown in fig. S1, A and B. (**C**) and (**D**) Yeast growth assay of *est2Δ* strain expressing either WT or mutant Est2 or empty pRS313 vector at 30 °C and 32 °C, respectively. Yeasts were plated after propagating for 120 generations.

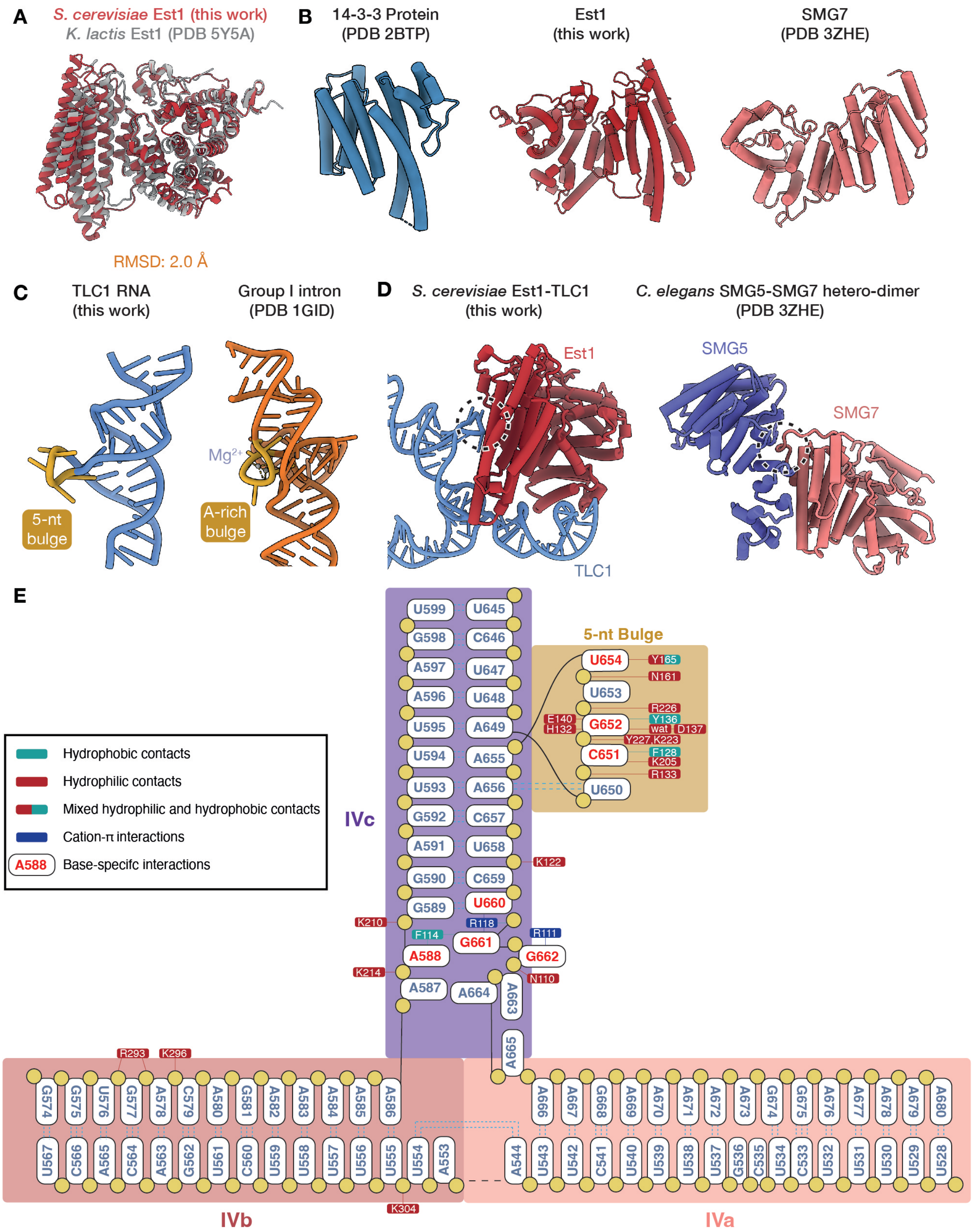

Fig. S14. Structure of Est1 in yeast telomerase. (**A**) Comparison of Est1 in our yeast telomerase structure with *K. lactis* Est1 (*25*). (**B**) Comparison of Est1 with published structures of its structural homologs, 14-3-3 and SMG7 proteins (*54, 115*). (**C**) Comparison of the 5-nt

bulge in stem IVc of TLC1 and the A-rich bulge in group I intron (*51*). (**D**) Comparison of Est1–TLC1 and SMG5–SMG7 (*54*). (**E**) Schematic showing Est1–TLC1 interacting residues.

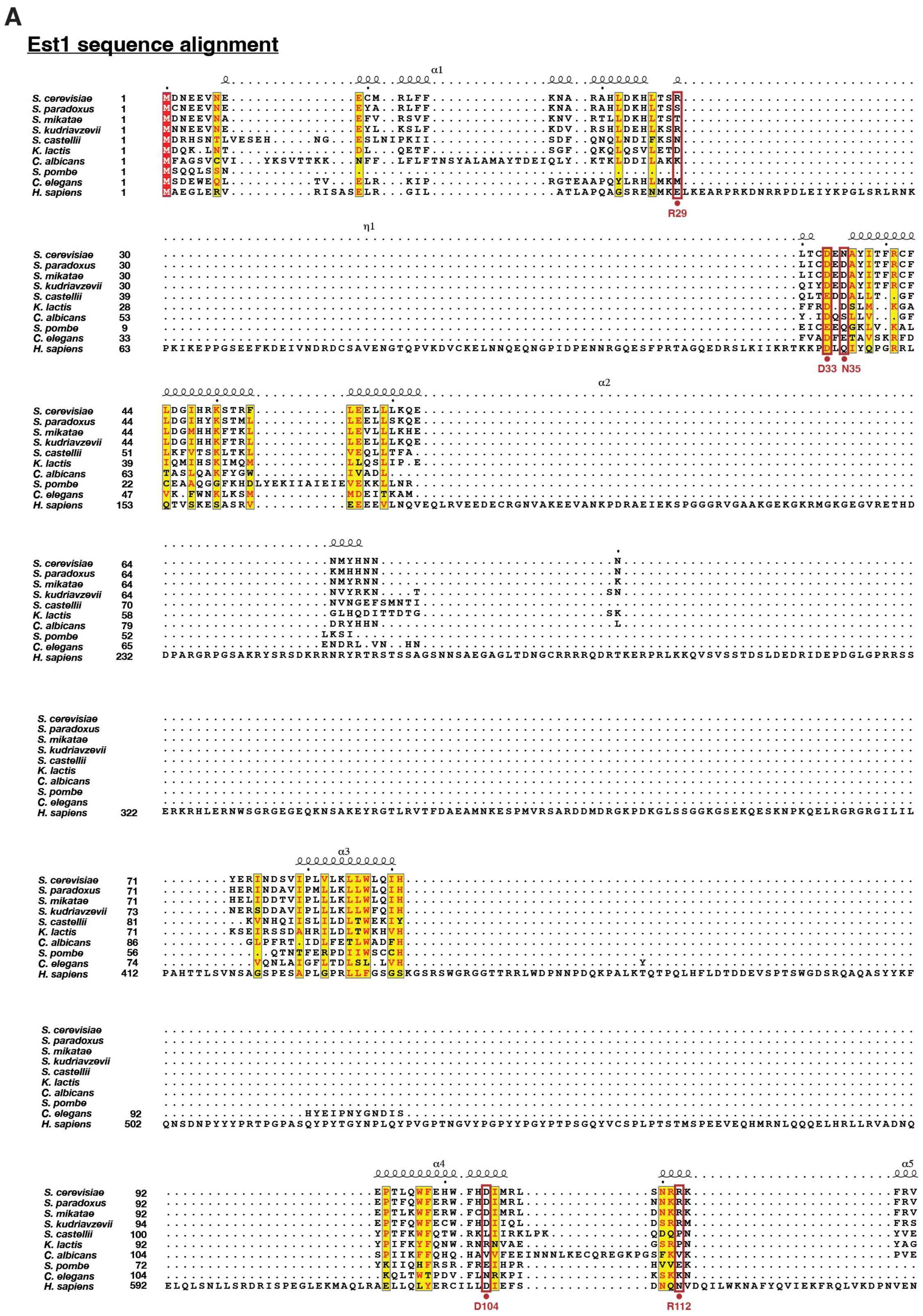
A
Est1 sequence alignment
S. cerevisiae
S. paradoxus
S. mikatae
S. kudriavzevii
S. castellii
K. lactis
C. albicans
S. pombe
C. elegans
H. sapiens
α1
η1
α2
α3
α4
α5
R29
D33
N35
D104
R112

Est1 sequence alignment (continued)

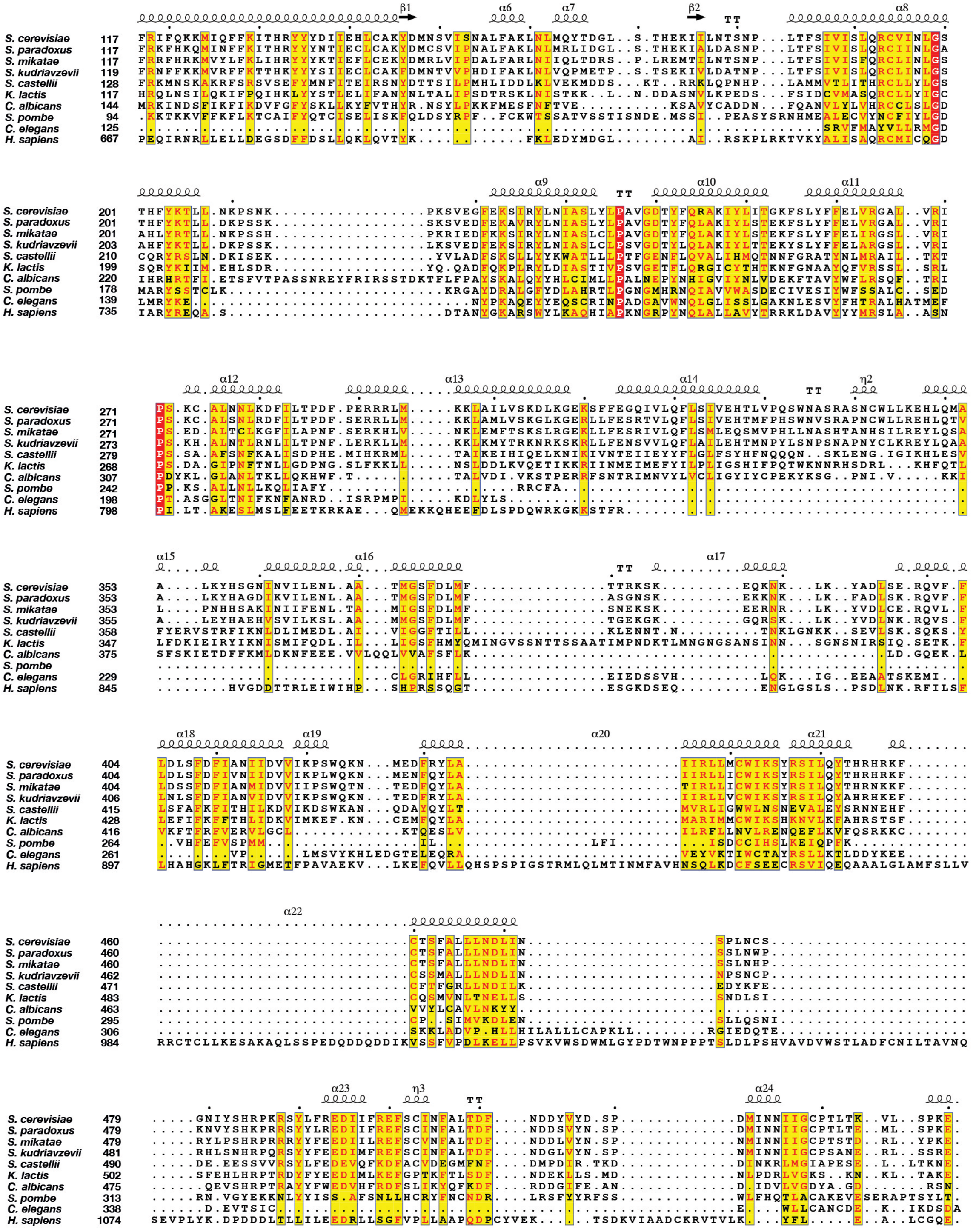

Est1 sequence alignment (continued)

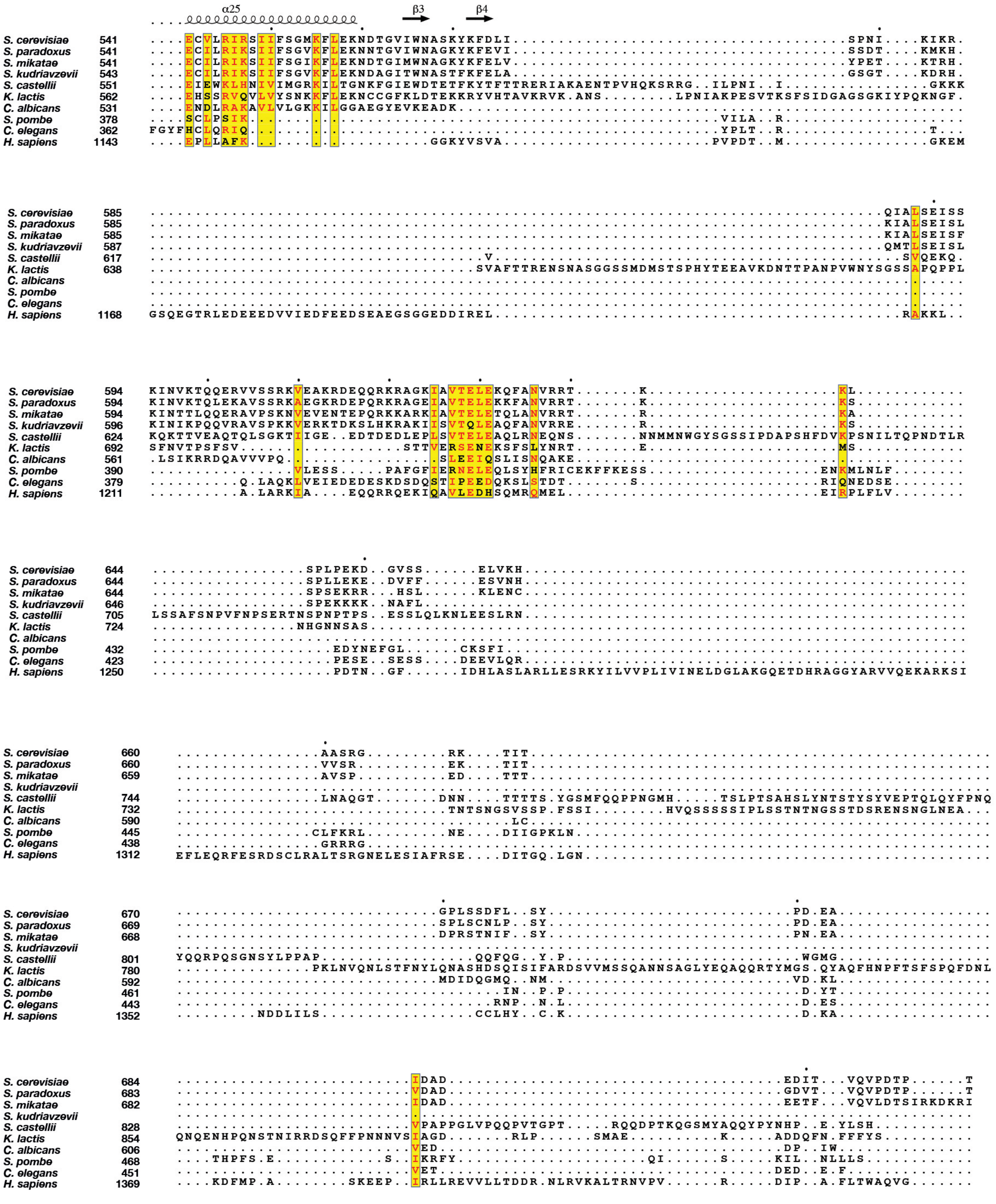

B

Pop1 sequence alignment

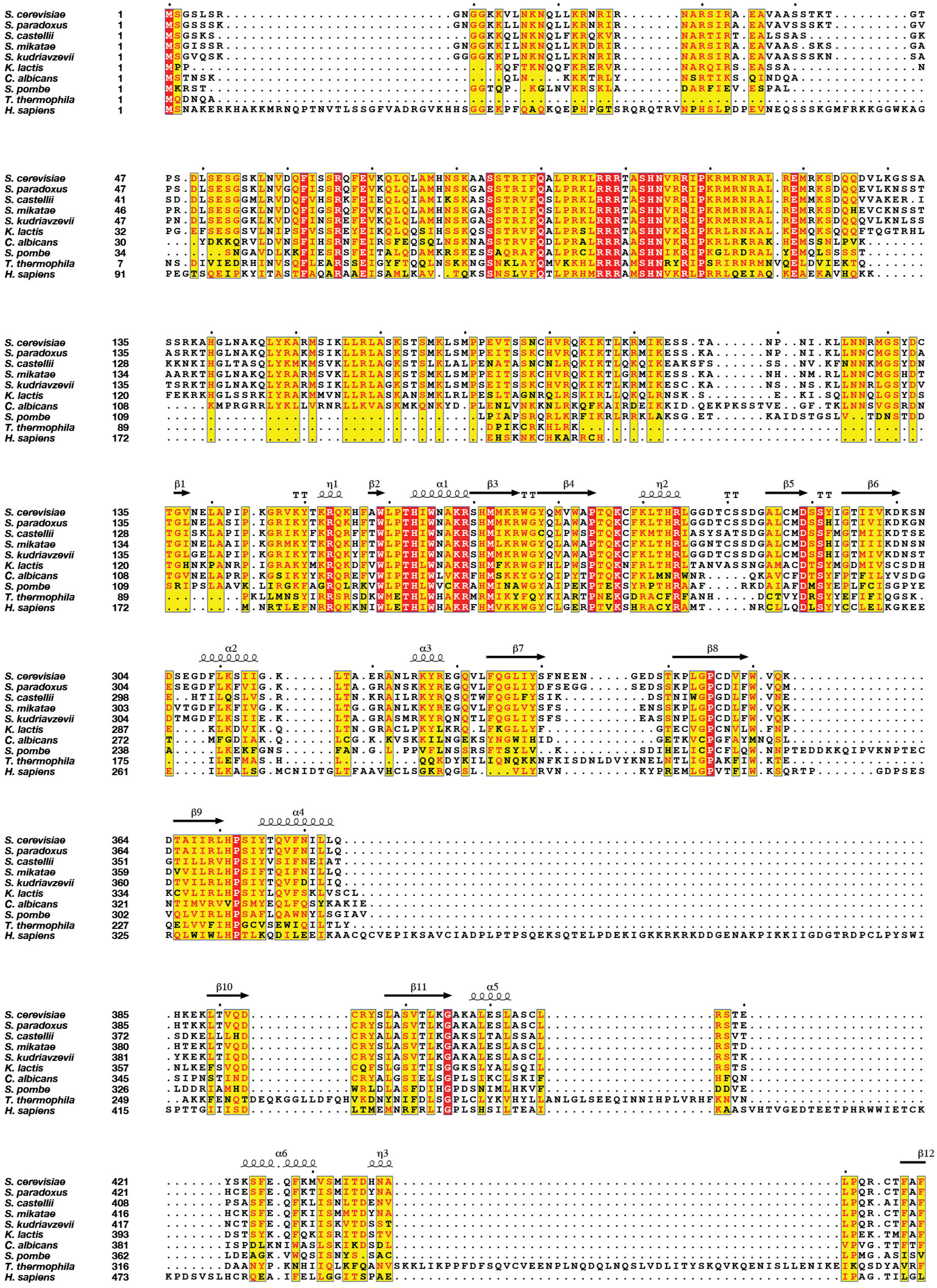

Pop1 sequence alignment (continued)

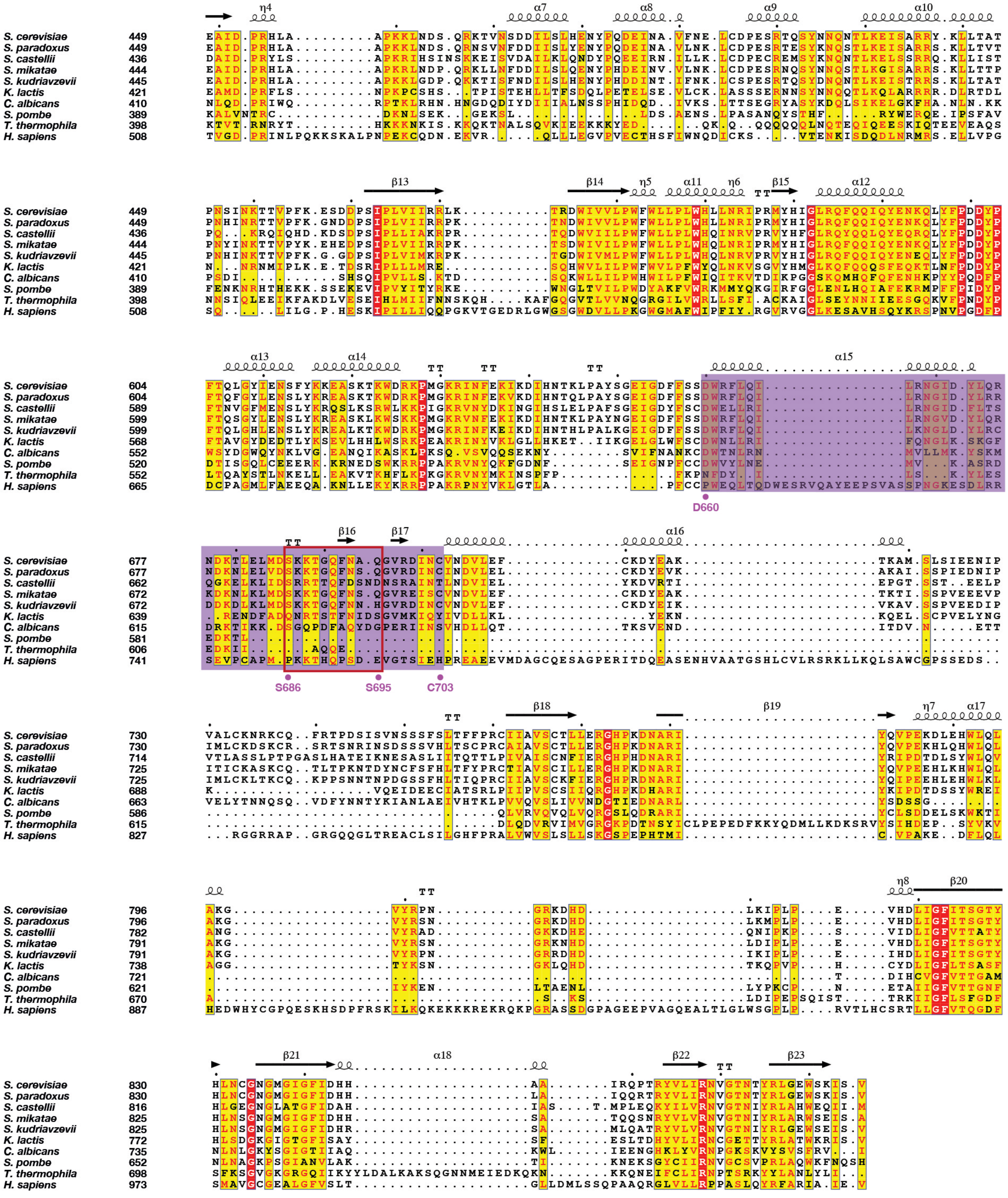

C

Pop6 sequence alignment

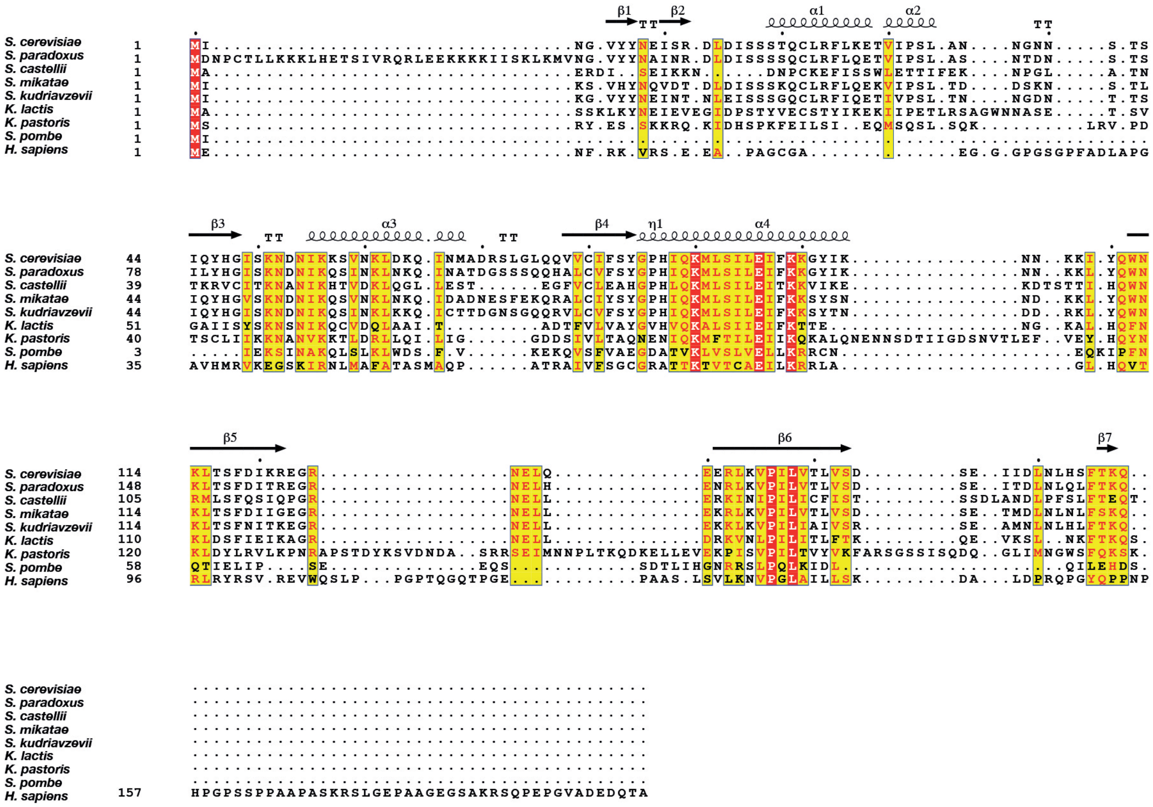

D

Pop7 sequence alignment

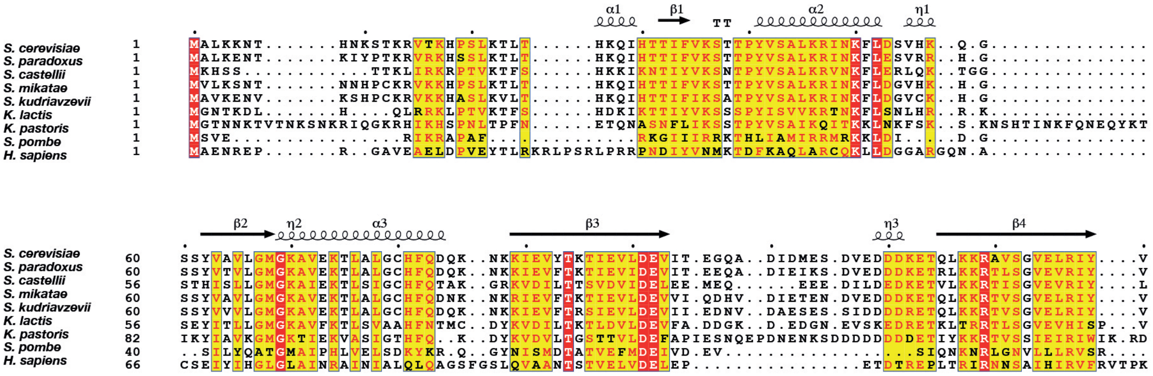

Fig. S15. Sequence alignment of Est1 and Pop proteins. (**A–D**) Sequence alignment of Est1, Pop1, Pop6 and Pop7, respectively. Residues in Est1 and Pop1 that are mutated in figs. S22 and S23 and Fig. 5 are highlighted.

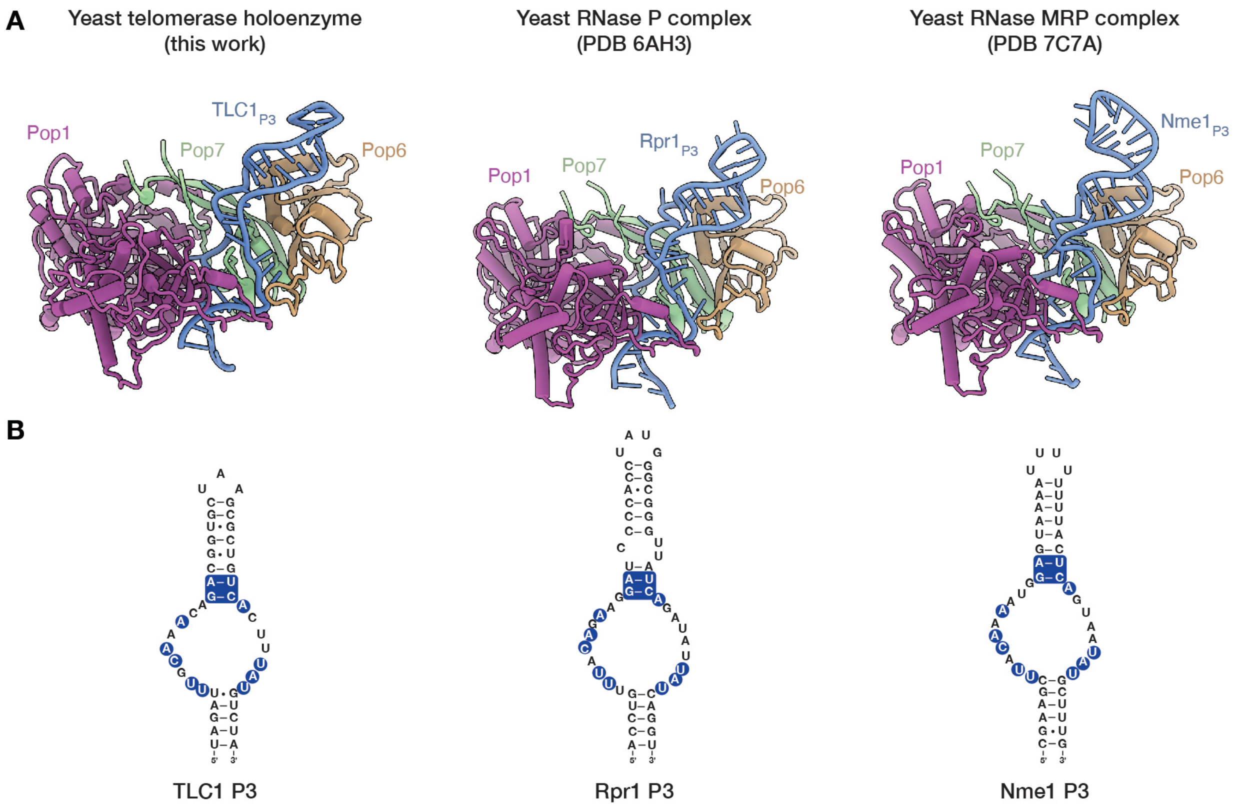

Fig. S16. Structural conservation of the RNA-bound Pop1/6/7 complex. (**A**) Comparison of the Pop1/6/7 complex bound to P3 domain of TLC1, Rpr1 and Nme1 in the structures of yeast telomerase and RNase P/MRP complexes, respectively (*72, 73*). (**B**) Comparison of the P3 domain of TLC1, Rpr1 and Nme1. Conserved residues are marked in blue.

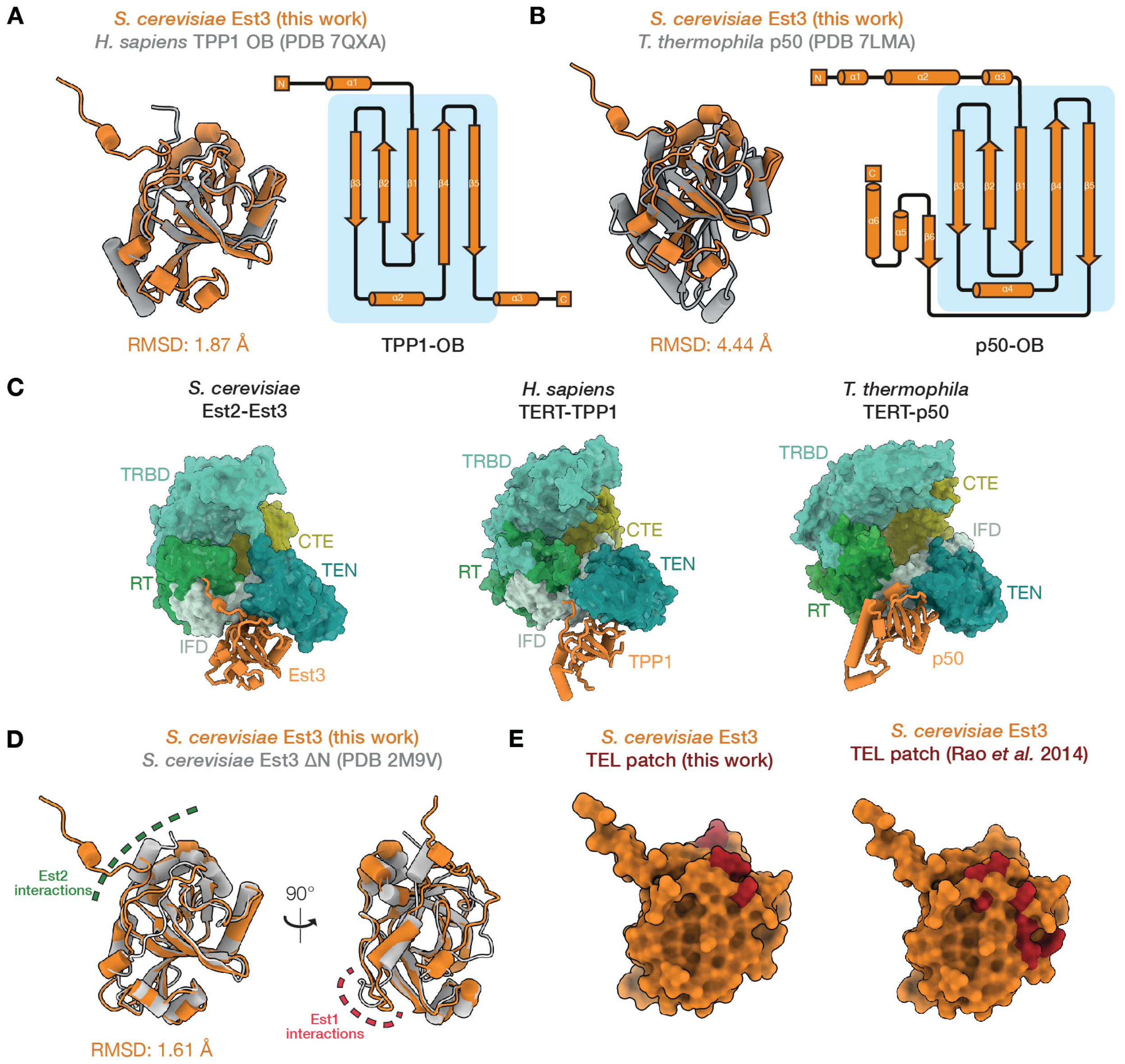

Fig. S17. Structure of Est3 in yeast telomerase. (**A**) Superposition of the structure of Est3 in the yeast telomerase holoenzyme onto the structure of TPP1 determined in complex with human telomerase (left panel) (*27*). The right panel shows the topological diagram of the OB-fold domain of TPP1. (**B**) Superposition of the structure of Est3 in the yeast telomerase holoenzyme onto the structure of *Tetrahymena* p50 as part of *Tetrahymena* telomerase (left panel) (*15*). The right panel shows the topological diagram of the OB-fold domain of p50. (**C**) Comparison of the Est2–Est3 interaction in yeast telomerase with the human TPP1–TERT interaction and the *Tetrahymena* p50–TERT interaction (*15, 27*). (**D**) Superposition of the structure of Est3 in the yeast telomerase holoenzyme onto the structure of Est3 determined alone (*60*). Regions that exhibit the largest change between the two structures are involved in Est2 and Est1 interactions, as indicated. (**E**)

Structure of Est3 in a space-filling presentation with the TEL patch redefined based on our yeast telomerase structure and that defined in previous work (*60*).

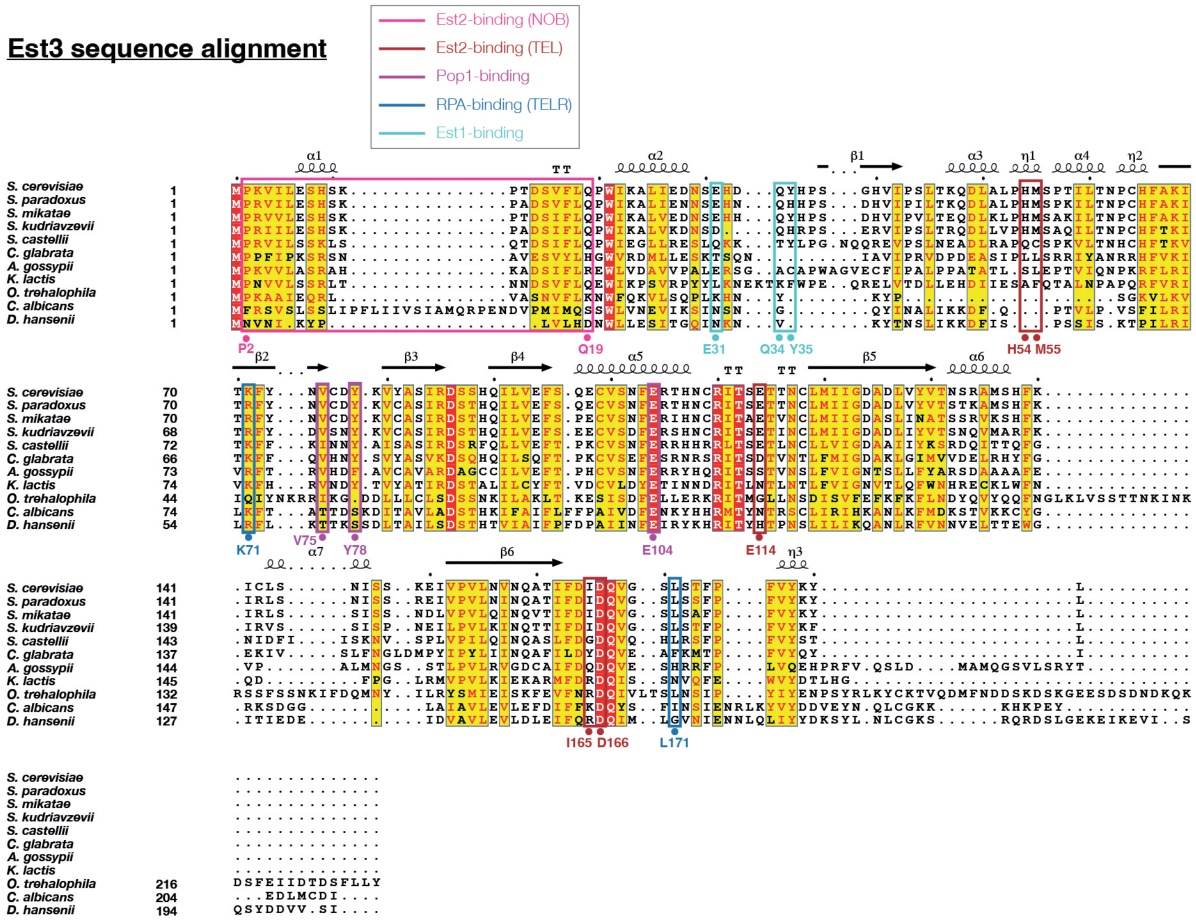

Fig. S18. Sequence alignments of Est3 among yeast species. Residues involved in interactions with Est1, Est2 and Pop1 are highlighted. We also highlight residues predicted to interact with RPA (*60, 86*).

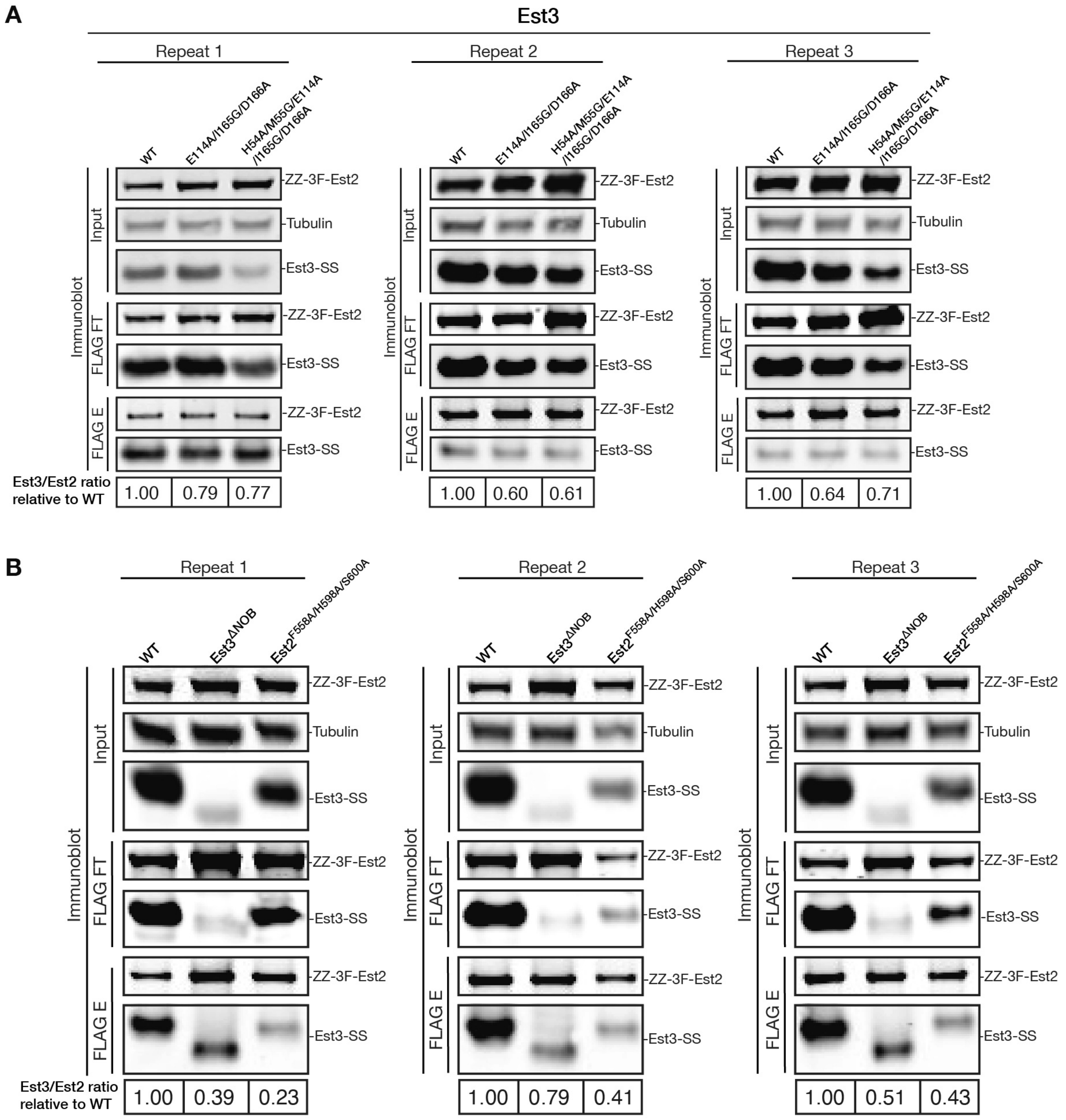

Fig. S19. Co-IP assays of Est2 and Est3. (**A**) Triplicates of co-IP assays of FLAG-tagged Est2 with Strep-tagged WT Est3 or Est3 TEL patch mutants. See also Fig. 4A. (**B**) Triplicates of co-IP assays of FLAG-tagged Est2 and Strep-tagged Est3 where the Est2–Est3–NOB interactions are disrupted using either Est3$^{\Delta NOB}$ mutant or Est2$^{F558A/H598A/S600A}$. See also Fig. 4C. In all co-IPs, Est2 and Est3 were added in excess of the beads as shown by the immunoblots of the FLAG flow-through (FLAG-FT) fractions. This ensures that similar amounts of Est2 were captured by the beads in each experiment and the levels of Est3 co-purified with Est2 were not limited by its initial expression.

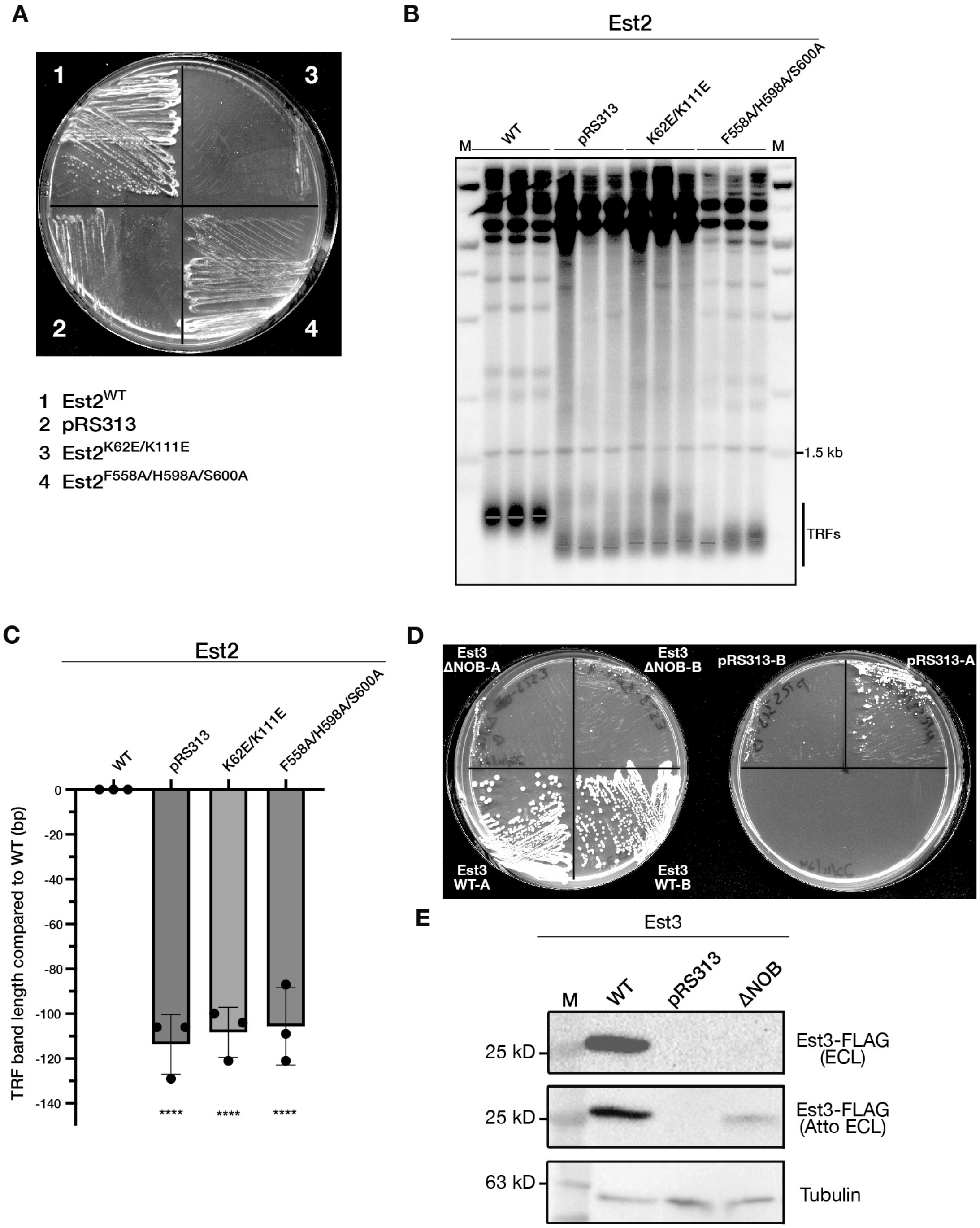

Fig. S20. Characterisation of the effects of disrupting Est2–Est3 interactions. (**A**) Yeast growth assay of *est2Δ* strain expressing either WT or mutant Est2 or empty pRS313 vector. Yeasts were plated after propagating for 120 generations. (**B**) TRF assay of *est2Δ* strain expressing either WT or mutant Est2 or empty pRS313 vector. Red lines indicate the mean telomere length for each lane. M, size markers. For each condition, TRFs from three independent clones are shown. (**C**) Quantification of the telomere length change in Est2 mutants relative to the WT Est2 in the TRF shown in **B**. (**D**) Yeast growth assay of *est3Δ* strain expressing either WT or Est3$^{\Delta NOB}$ or empty

pRS313 vector. Two clones are shown. (**E**) Immunoblots showing the levels of Est3 expression in the yeast strains shown in **D**. Error bars shown in **C** represent standard deviation. ****, $P \leq 0.0001$.

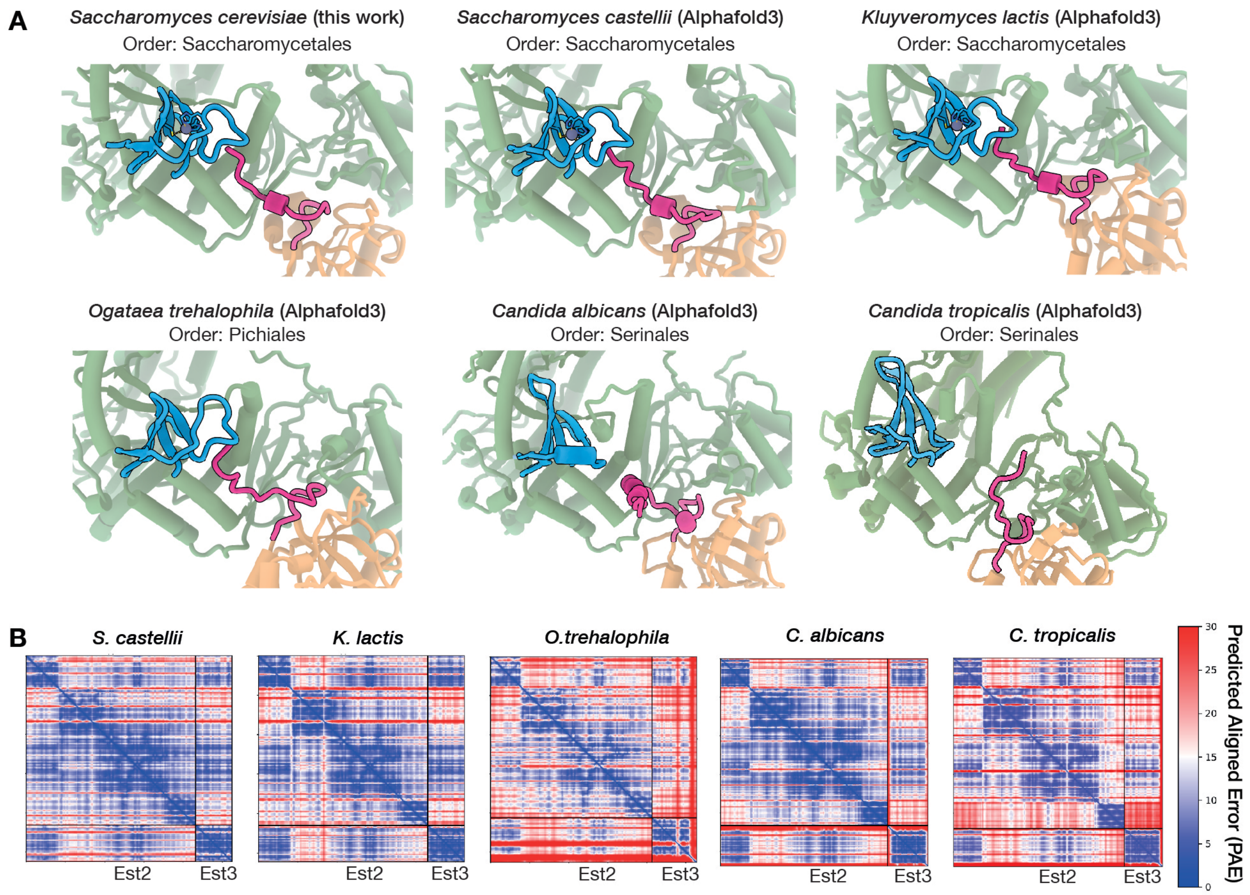

Fig. S21. The Est3 NOB and its interaction with the ZnF motif. (**A**) AlphaFold3 prediction Est2–Est3 complex for several yeast species. The interaction between the Est3 NOB and the ZnF motif of Est2 observed in our yeast telomerase structure is predicted to be conserved in species in the Saccharomycetales clade but not in other clades. (**B**) Predicted alignment error (PAE) plots for the predicted structures shown in **A**.

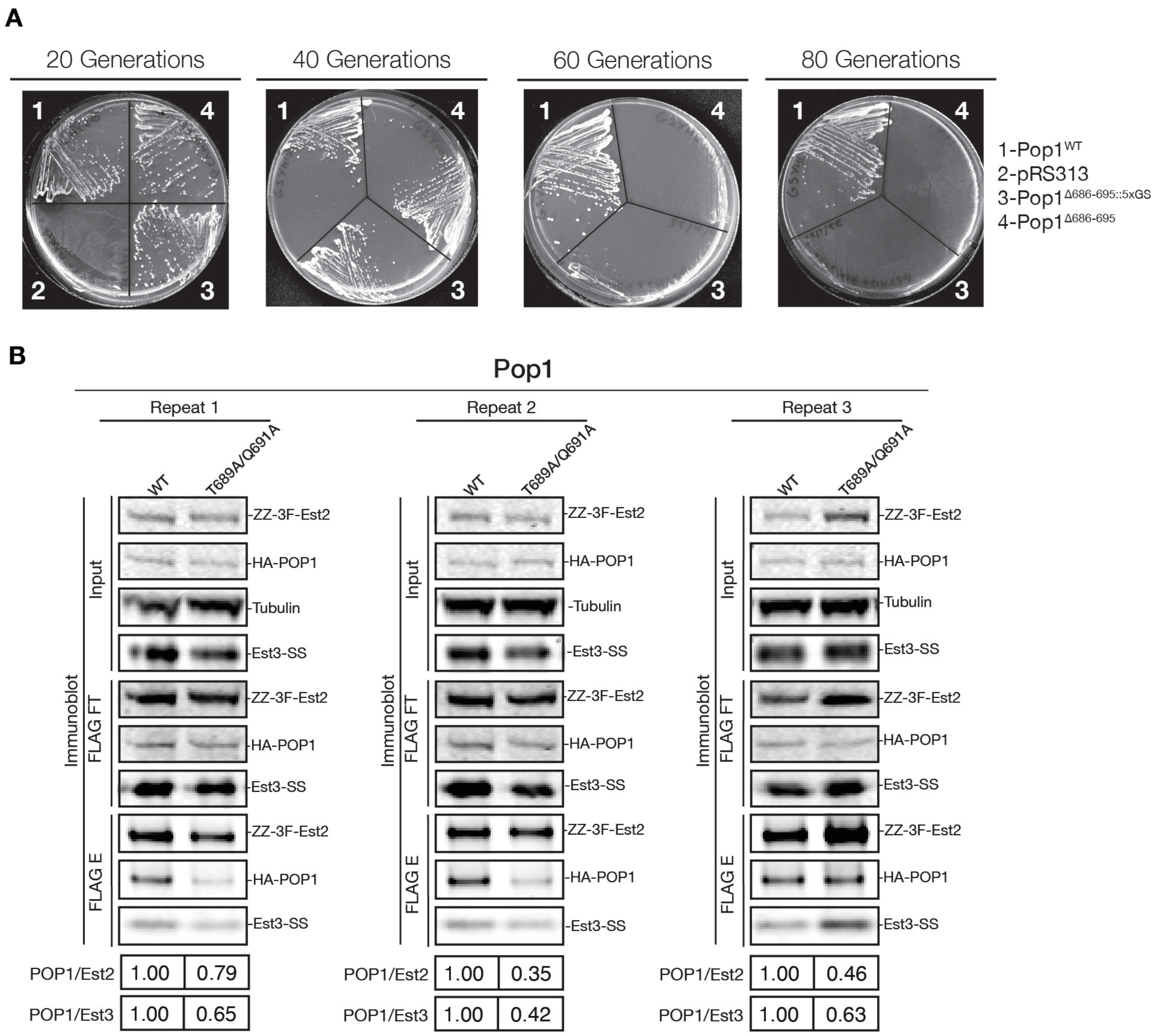

Fig. S22. Characterisation of the effects of disrupting the Pop1-hanger. (**A**) Yeast growth assay of *pop1Δ* strain expressing either WT or mutant Pop1 or empty pRS313 vector. The number of generations is indicated. See also Fig. 5D. (**B**) Triplicates of the co-IP assays of FLAG-tagged Est2 with HA-tagged Pop1 and Strep-tagged Est3. See also Fig. 5, G to I. In these assays, WT Est2 and Est3 are expressed in *pop1Δ* strain expressing either WT or Pop1$^{T689A/Q691A}$ mutant. Immunoblots detecting Est2, Est3 and Pop1 levels using antibodies against FLAG, Strep and HA tags, respectively, are shown. In all co-IPs, Est2, Est3 and Pop1 were added in excess of the beads as shown by the immunoblots of the FLAG flow-through (FLAG-FT) fractions. This ensures that similar amounts of Est2 were captured by the beads in each experiment and the levels of Est3 and Pop1 co-purified with Est2 were not limited by its initial expression.

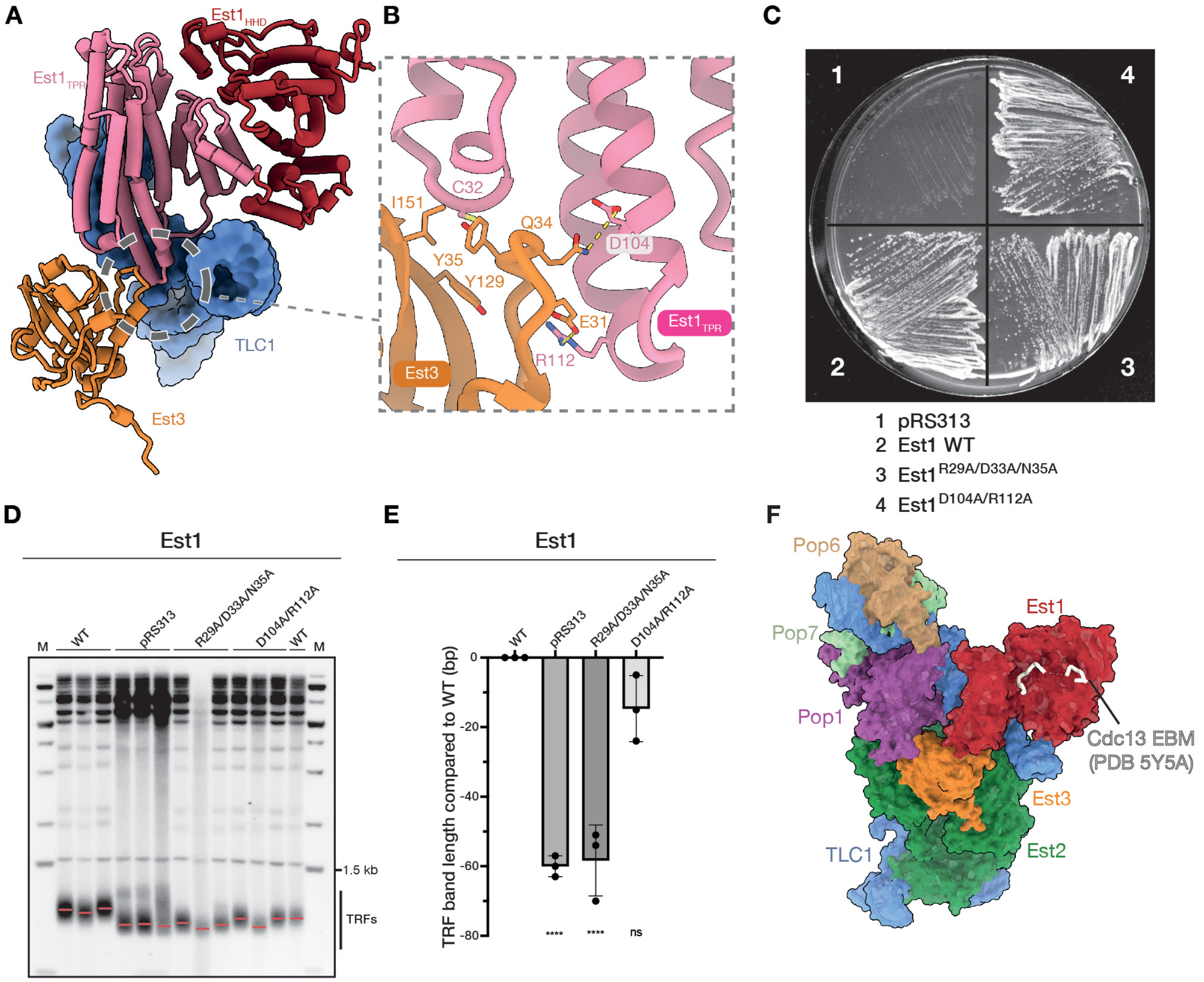

Fig. S23. Characterisation of Est3 interaction with Est1. (**A**) Overall view of Est1–Est3 interactions. (**B**) Close-up view of the Est1–Est3 binding interface. (**C**) Yeast growth assay of *est1Δ* strain expressing either WT or mutant Est1 or empty pRS313 vector. Yeasts were plated after propagating for 100 generations. (**D**) TRF assay of *est1Δ* strain expressing either WT Est1 or Est1 mutants or empty pRS313 vector. Residues mutated in Est1 are involved in interaction with Est3 as shown in **B**. Red lines indicate the mean telomere length for each lane. M, size markers. For each condition, TRFs from three independent clones are shown. (**E**) Quantification of the telomere length change in Est1 mutants relative to the WT Est1 in the TRF shown in **D**. (**F**) Model of the yeast telomerase holoenzyme with the Cdc13 Est1 binding motif (EBM) as determined in previous work *(25)*. Error bars shown in **E** represent standard deviation (SD). ****, $P \leq 0.0001$; ns, not significant.

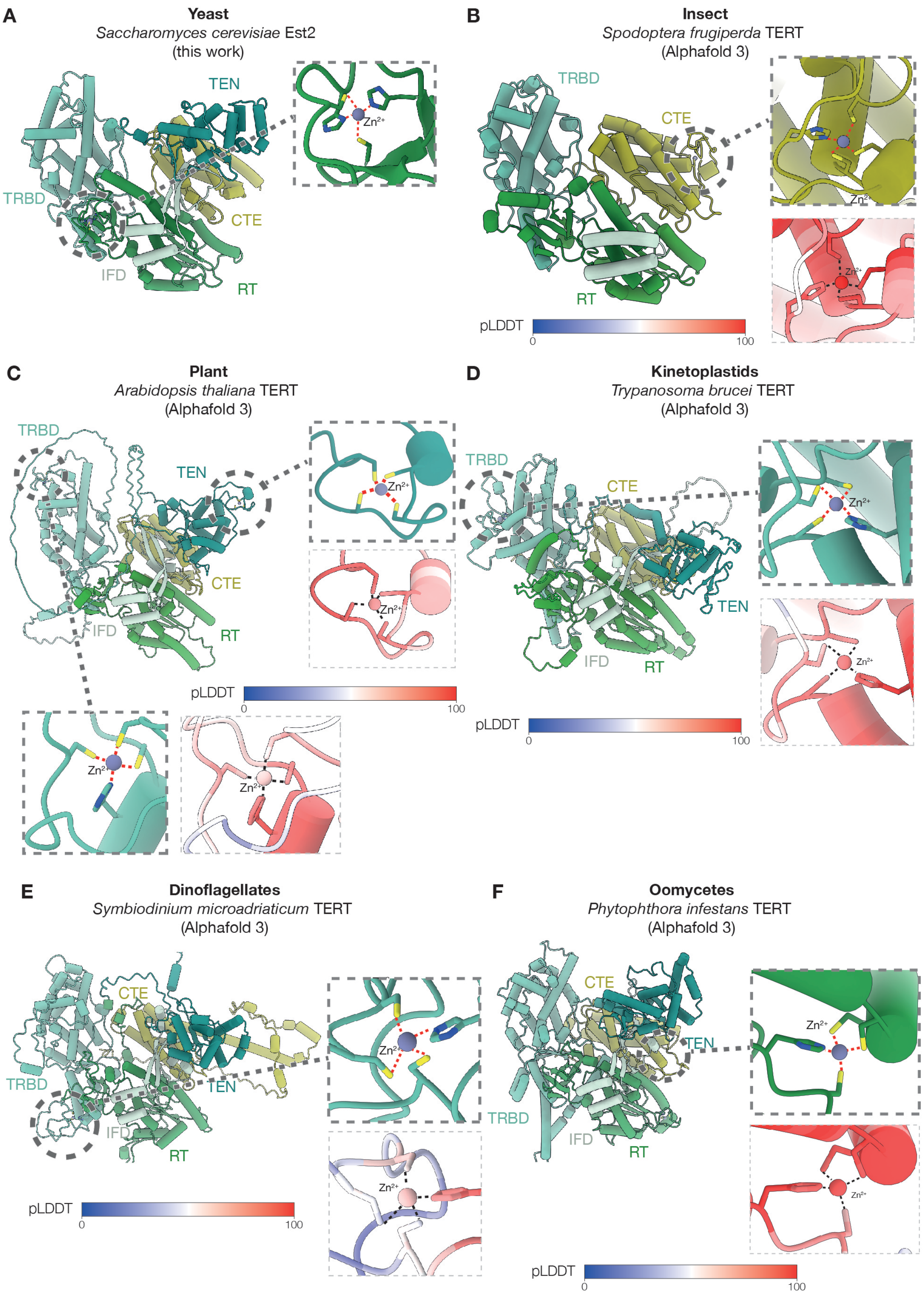

Fig. S24. ZnF motifs are predicted in TERT from different eukaryotic species. (**A**) Structure of the *Saccharomyces cerevisiae* Est2 (left) and its ZnF motif (right). (**B–F**), Alphafold3 prediction

of TERT from insect species *Spodoptera frugiperda*, plant species *Arabidopsis thaliana*, human-infective parasite *Trypanosoma brucei*, photosymbiotic microbe *Symbiodinium microadriaticum* and plant pathogen *Phytophthora infestans*, respectively. The dashed circle indicates the location of the predicted ZnF motif. For each ZnF motif, two insets with the close-up view of each predicted ZnF motif are shown. One inset is colored according to TERT domain colors as shown in Fig. 2A; the other inset is colored according to the pLDDT scores of the AlphaFold prediction.

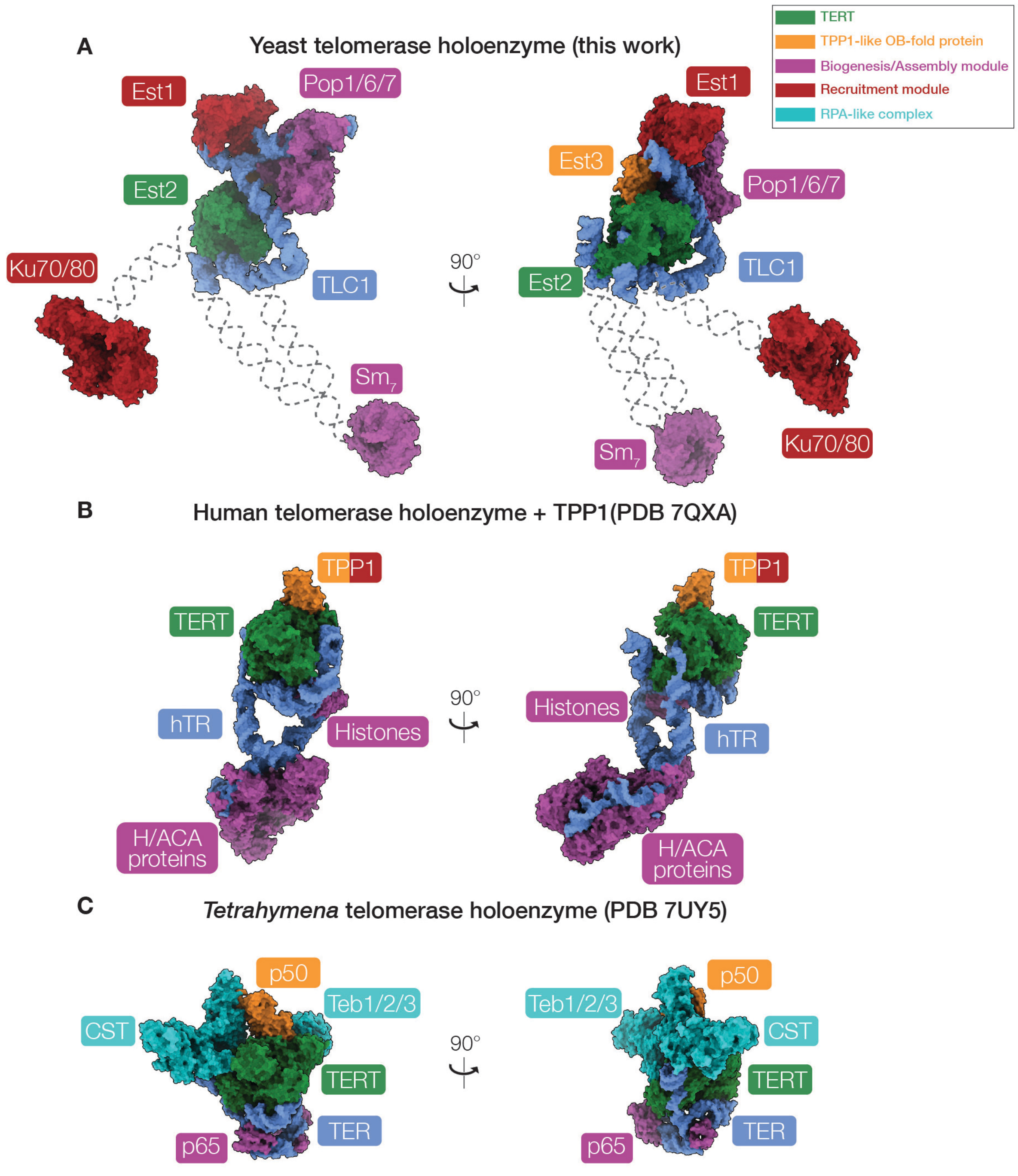

Fig. S25. Comparison of the structures of yeast, human and *Tetrahymena* telomerase holoenzymes. (**A–C**) Structures of the budding yeast (this study), human and *Tetrahymena* telomerases, respectively (*14, 27, 116*). The shown structures are aligned relative to TERT (or Est2 in yeast). Functional protein modules are colored as indicated. TPP1 is involved in telomerase recruitment in humans, thus it is also colored red.

Table S1. Mass spectrometry analysis of (A) the purified yeast telomerase sample in solution and (B) the gel band indicated in the red box.

A

Accession Code	Protein name	Number of peptides	Coverage (%)	Length (AA)	Molecular weight (kD)
Q06163	Est2	32	37	884	102.6
P41812	Pop1	32	38	875	100.3
Q06217	SmD2	4	28	110	12.8
P17214	Est1	16	23	699	81.7
P38291	Pop7	6	33	140	15.8
P40018	SmB	10	45	196	22.4
P53218	Pop6	6	47	158	18.2
Q02260	SmD1	5	30	146	16.3
P43321	SmD3	4	62	101	11.2
P54999	SmF	3	41	86	9.7
P40204	SmG	1	12	77	8.5
Q12330	SmE	1	16	94	10.4

B

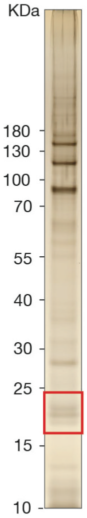

Accession Code	Protein name	Number of peptides	Coverage (%)	Length (AA)	Molecular weight (kD)
Q02260	SMD1	5	36	146	16.3
P53218	Pop6	5	24	158	18.2
P38291	Pop7	4	25	140	15.8
Q06217	SMD2	3	26	110	12.8
Q03096	Est3	2	9	181	20.5
O44437	SmD3	4	22	101	11.2

Table S2. Cryo-EM data collection, refinement, and validation statistics

	Yeast telomerase overall structure<br>PDB 9SWN<br>EMD-55321	Yeast telomerase catalytic core<br>PDB 9SWO<br>EMD-55322
**Data collection and Processing**		
Microscope	TFS Titan Krios	
Voltage (keV)	300	
Camera	Falcon 4i	
Magnification	130,000	
Pixel size at detector (Å/pixel)	0.955	
Total electron exposure ($e^{-}/Å^2$)	50	
Exposure rate (e-/ $Å^2$/sec)	11.12	
Number of frames	50	
Defocus range (µm)	1.0-2.4	
Automation software	EPU	
Energy filter slit width	10 eV	
Micrographs collected (no.)	24,936	
Total extracted particles (no.)	1,876,851	
**For each reconstruction**		
Final particles (no.)	125,104	125,104
Point-group	C1	C1
Resolution (global, Å)		
FSC 0.5 (unmasked/masked)	4.44/3.49	8.28/4.17
FSC 0.143 (unmasked/masked)	3.51/3.03	3.85/3.74
Resolution range (local, Å)	2.86-11.82	3.48-11.11
3DFSC Sphericity	0.952	0.733
Map sharpening *B* factor ($Å^2$)	-50	-50
Map sharpening methods	CryoSPARC	CryoSPARC
**Model composition**		
Protein (residues)	2,572	878
RNA (nucelotides)	232	98
Ligand	3	2
Water	2	-
**Model Refinement**		
Refinement package	Phenix/Servalcat	Phenix/Servalcat
Real or reciprocal space	Reciprocal	Reciprocal
Resolution cutoff	0.5	0.5
Model-Map scores		
CCvolume/mask	0.81/0.82	0.80/0.81
B factors ($Å^2$)		
Protein residues (min/max/mean)	26.04/327.97/138.65	28.49/799.36/208.04
RNA (min/max/mean)	30.45/339.83/193.60	131.51/845.02/509.00
Ligand (min/max/mean)	88.48/226.30/147.71	64.49/164.49/114.49
Water (min/max/mean)	50.63/51.37/51.00	-
R.m.s. deviations from ideal values		
Bond lengths (Å) (#>4σ)	0.009 (0)	0.008 (0)

Bond angles (°) (#>4σ)	1.350 (12)	1.291 (8)
**Validation**		
MolProbity score	1.50	1.56
CaBLAM outliers (%)	1.89	2.30
Clashscore	4.04	4.70
Poor rotamers (%)	0.04	0
C-beta deviations (%)	0	0
EMRinger score	3.14	2.05
Ramachandran plot		
Favored (%)	95.58	95.42
Outliers (%)	0	0

Table S3. AlphaFold3 prediction of TERTs in a diverse range of species

Classifications			Species	Accession code	Potential zinc finger motif
**Fungi**	Ascomycetes	Saccharomycetales	*Saccharomyces cerevisiae*	Q06163	+
			Saccharomyces castellii	G0VKP3	+
			Kluyveromyces lactis	Q6CSS0	+
		Saccharomycodales	*Hanseniaspora osmophila*	A0A1E5RAT1	+
			Hanseniaspora occidentalis	SRHL01000009.1	+
		Phaffomycetales	*Wickerhamomyces ciferrii*	K0KF00	-
		Serinales	*Candida albicans*	A0A1D8PEA0	-
			Candida tropicalis	C5MCQ7	-
			Debaryomyces hansenii	Q6BUF6	-
		Pichiales	*Ogataea trehalophila*	PPKI02000001.1	-
			Komagataella pastoris	A0A1B2JA37	-
		Dipodascales	*Yarrowia lipolytica*	A0A1D8NE89	-
		Schizosaccharomycetales	*Schizosaccharomyces pombe*	O13339	-
		Sordariales	*Neurospora crassa*	F5CST9	-
	Basidiomycetes	Ustilaginales	*Ustilago maydis*	A0A0D1EAT9	-
		Agaricales	*Agaricus bisporus*	A0A8H7F1V6	-
**Animals**	Insects	Lepidoptera	*Bombyx mori*	Q19S69	+
			Spodoptera frugiperda	A0A9R0D6U6	+
			Trichoplusia ni	A0A7E5VNM5	+
			Maniola jurtina	XP_045780426.1	+
			Vanessa cardui	XP_046973017.1	+
		Coleoptera	*Tenebrio molitor*	A0A8J6HWQ0	-
		Orthoptera	*Anabrus simplex*	XP_067004681.2	-
		Hemiptera	*Planococcus citri*	XP_065200193.1	-
		Hymenoptera	*Bombus affinis*	XP_050598260.1	-
			Apis cerana	A0A2A3E1G5	-
		Blattodea	*Embiratermes neotenicus*	A0A8B0H871	-
	Mammals	Primates	*Homo sapiens*	O14746	-
		Rodentia	*Mus musculus*	O70372	-
		Carnivora	*Canis familiaris*	Q6A548	-
		Monotremata	*Ornithorhynchus anatinus*	F2YSU1	-
	Birds	Galliformes	*Gallus gallus*	A0A8V0XWC6	-
	Reptiles	Squamata	*Anolis carolinensis*	G1K9H7	-
	Amphibians	Anura	*Xenopus laevis*	Q9DE32	-
	Fish	Cypriniformes	*Danio rerio*	A2THE9	-
	Crustaceans	Decapoda	*Metapenaeus ensis*	A0A1B1SN26	-
	Tardigrades	Parachela	*Ramazzottius varieornatus*	A0A1D1W0H3	-
	Arachnids	Ixodida	*Dermacentor andersoni*	XP_050046898.2	-
	Nematodes	Rhabditida	*Caenorhabditis elegans*	NP_492373.1	-
	Brachiopods	Lingulida	*Lingula anatina*	XP_013380608.1	-
	Mollusca	Architaenioglossa	*Pomacea canaliculata*	A0A2T7P9I0	-
		Ostreida	*Crassostrea virginica*	A0A7G6KMQ8	-
		Octopoda	*Octopus vulgaris*	A0A7E6FS56	+
			Octopus bimaculoides	A0A7G6KMQ9	+
	Echinoderm	Forcipulatida	*Asterias rubens*	XP_033637717.1	-
		Valvatida	*Acanthaster planci*	A0A8B7YJT6	-
			Patiria miniata	A0A913ZHK7	-
		Camarodonta	*Lytechinus pictus*	XP_063967610.1	-
	Cnidaria	Scleractinia	*Orbicella faveolata*	XP_020629467.1	-
	Porifera	Dictyoceratida	*Dysidea avara*	XP_065897050.1	-
**Green plants**	Angiosperms	Brassicales	*Arabidopsis thaliana*	Q9SPU7	+, multiple
			Brassica oleracea	A0A0D3EEX2	+, multiple
		Solanales	*Nicotiana tabacum*	F4YIM8	+, multiple
			Solanum lycopersicum	A0A3Q7EJC6	+, multiple
		Poales	*Oryza sativa*	Q8LKW0	+, multiple
			Zea mays	Q1EG35	+, multiple
			Triticum aestivum	A0A9R1MZ90	+, multiple
	Gymnosperms	Cupressales	*Cryptomeria japonica*	XP_057849916.2	+
	Fern	Polypodiales	*Adiantum capillus-veneris*	A0A9D4UBK4	-
	Liverwort	Marchantiales	*Marchantia polymorpha*	A0A2R6XL99	-
	Moss	Funariales	*Physcomitrella patens*	A0A0C5BXA6	-
**Red algae**	Cyanidiophyceae	Cyanidiales	*Cyanidium caldarium*	A0AAV9ISE6	+
			Cyanidioschyzon merolae	M1V6S8	+
			Galdieria sulphuraria	M2W5R3	-
**Green algae**	Mamiellophyceae	Mamiellales	*Ostreococcus tauri*	B5TFN0	-
**Excavates**	Kinetoplastids	Trypanosomatida	*Trypanosoma brucei*	A0A3L6KUB5	+
			Trypanosoma cruzi	A0A7J6XWD8	+
			Leishmania braziliensis	Q2NNR2	-
		Eubodonida	*Bodo saltans*	A0A0S4JIY5	-
	Oxymonads	Oxymonadida	*Monocercomonoides exilis*	XP_067734963.1	+
	Diplomonads	Diplomonadida	*Giardia intestinalis*	Q9NCP5	-
**Stramenopiles**	Oomycetes	Peronosporales	*Phytophthora infestans*	A0A833SSQ5	+
			Phytophthora cinnamomi	XP_067791751.1	+
			Phytophthora ramorum	H3H5W8	+
			Plasmopara halstedii	A0A0P1A6J1	+
		Saprolegniales	*Aphanomyces astaci*	A0A425CSN9	-
**Alveolates**	Dinoflagellates	Suessiales	*Symbiodinium microadriaticum*	A0A1Q9CMC1	+
			Polarella glacialis	A0A813DH25	+
	Ciliates	Hymenostomatida	*Tetrahymena thermophila*	O77448	-
		Sporadotrichida	*Sterkiella histriomuscorum*	O76332	-
**Rhizaria**	Cercozoa	Plasmodiophorida	*Spongospora subterranea*	A0A0H5RFS0	-
**Amoebas**	Discosea	Centramoebida	*Acanthamoeba castellanii*	L8GIB5	-

Table S4. Yeast strains used in this study

**Strain**	**Genotype**	**Source**
W3749	*MatA can1-100 ura3-1 his3-11,15 leu2-3,112 trp1-1 bar1Δ::LEU2*	Lisby et al. 2004 (*117*)
EDL221	*Mat alpha, can1-100, ura3-1, his3::NatR::TDH3p-MS2-PrA, leu2-3,112, trp1-1, bar1Δ::LEU2, TLC1/TLC1-10xMS2, est1Δ::TRP1+ p316-EST1wt (URA3)*	This study
EDL223	*Mat alpha, can1-100, ura3-1, his3::NatR::TDH3p-MS2-PrA, leu2-3,112, trp1-1, bar1Δ::LEU2, TLC1/TLC1-10xMS2, est3Δ::TRP1 + YPC33-EST3wt (URA3)*	This study
JNY262	*W3749A est2Δ::TRP1 +pRS316 EST2wt (URA3)*	This study
GSYH12	*W3749 pop1Δ::KanMX + pRS316-3HA-POP1wt (URA3)*	Neumann et al. 2023 (*83*)
BCY123	*MATα pep4::HIS3 prb1::LEU2 bar1::HIS6 lys2::GAL1/10-GAL4 can1 ade2 trp1 ura3 his3 leu2-3,112*	Wasserman and Wang, 2004 (*87*)

Table S5. Plasmids used in this study

Plasmid	Features	Source
pRS313	Single copy yeast vector with *HIS3* selectable marker	Sikorski and Hieter, 1989 (*118*)
pRS313-POP1	*HIS3, CEN, HA_3-POP1*	Lemieux et al. 2016 (*18*)
pRS313-POP1$^{Δ686\text{-}695}$	*HIS3, CEN, HA_3-POP1(Δ686-695)*	This study
pRS313-POP1$^{Δ686\text{-}695::5xGS}$	*HIS3, CEN, HA_3-POP1 (S_{686}KKTGQFNAQ_{695} to 5xGS)*	This study
pRS313-POP1$^{T689A/Q691A}$	*HIS3, CEN, HA_3-POP1 (Q691A T689A)*	This study
pRS313-EST1	*HIS3, CEN, EST1-18xMYC*	This study
pRS313-EST1$^{R29A/D33A/N35A}$	*HIS3, CEN, EST1-18xMYC (D33A N35A R29A)*	This study
pRS313-EST1$^{D104A/R112A}$	*HIS3, CEN, EST1-18xMYC (D104A R112A)*	This study
pRS313-EST2	*HIS3, CEN, $FLAG_3$-Gly6-EST2*	This study
pRS313-EST2$^{K62E/K111E}$	*HIS3, CEN, $FLAG_3$-Gly6-EST2 (K62E K111E)*	This study
pRS313-EST2 $^{F558A/H598A/S600A}$	*HIS3, CEN, $FLAG_3$-Gly6-EST2 (H598A S600A F558A)*	This study
pRS313-EST2-ZnF-M1	*HIS3, CEN, $FLAG_3$-Gly6-EST2 (C422A H425A H435A C623A)*	This study
pRS313-EST2-ZnF-M2	*HIS3, CEN, $FLAG_3$-Gly6-EST2 (C422A H425A)*	This study
pRS313-EST2-ZnF-M3	*HIS3, CEN, $FLAG_3$-Gly6-EST2 (H435A C623A)*	This study
pRS313-EST2-ZnF-M4	*HIS3, CEN, $FLAG_3$-Gly6-EST2 (C422A)*	This study
pRS313-EST2-ZnF-M5	*HIS3, CEN, $FLAG_3$-Gly6-EST2 (H425A)*	This study
pRS313-EST2-ZnF-M6	*HIS3, CEN, $FLAG_3$-Gly6-EST2 (H435A)*	This study
pRS313-EST2-ZnF-M7	*HIS3, CEN, $FLAG_3$-Gly6-EST2 (C623A)*	This study
YCPlac33-EST3	*URA3, CEN, EST3-$FLAG_3$*	This study
pRS313-EST3	*HIS3, CEN, EST3-$FLAG_3$*	This study
pRS313-EST3$^{E114A/I165G/D166A}$	*HIS3, CEN, EST3-$FLAG_3$ (E114A I165G D166A)*	This study
pRS313-EST3$^{H54A/M55G\ E114A/I165G/D166A}$	*HIS3, CEN, EST3-$FLAG_3$ (E114A I165G D166A H54A M55G)*	This study
pRS426-ZZ-3xF-Est2-Est1-Est3	*URA3, 2μ, ZZ-$FLAG_3$-Est2, Est1, Est3*	This study
pRS426-ZZ-3xF-Est2-ZnF M1-Est1-Est3-SS	*URA3, 2μ, ZZ-$FLAG_3$-Est2 (C422A H425A H435A C623A), Est1, Est3-Twin-Strep*	This study
pRS426-ZZ-3xF-Est2$^{K62E/K111E}$ -Est1-Est3-SS	*URA3, 2μ, ZZ-$FLAG_3$-Est2 (K62E K111E), Est1, Est3-Twin-Strep*	This study

Table S6. Sequences of DNA oligonucleotides use in this study

A. DNA primers used for cloning Est2, Est3, and Est1 from genomic DNA.

Purpose	Primers
Cloning of ZZ-Est2	5′-ATTATAACATGTCAATGAAAACCGCGGCTCTTGC-3′
	5′-CGAATAAGATTTTCATGGTACCAGAGGCAGTAGTTGG-3′
	5′-CCAACTACTGCCTCTGGTACCATGAAAATCTTATTCG-3′
	5′-TATTTAGGATCCCTAATTAACTATATGTATATATATATATATATATGC-3′
Cloning of Est3 gene	5′-ATTATACCATGGGCATGCCGAAAGTAATTCTGG-3′
	5′-TATACTGGATCCTCATAAATATTTATATACAAATGGG-3′
Cloning of Est1 gene	5′-TATTCACCATGGATAATGAAGAAGTTAACG-3′
	5′-ATTATAGGATCCTCAAGTAGGAGTATCTGGCACTTGG-3′

B. DNA primers used for the mutagenesis of Est2 and Est3 in co-immunoprecipitation and telomerase activity assay experiments.

Construct	Mutation/Deletion	Primers
pRS426-ZZ-3xF-Est2-Est1-Est3-SS	Est2 C623A/C422A	5’-TGAAGATAAGGCGTACATTAGAGAAGATGGTCTTTTTC -3′
		5′-ACCCACAAAGCTGTTTTAAATATTTC-3′
		5′-AAACAACGTAGCGAGAAACCATAATAGTTACACG-3′
		5′-TCGACTAAGTACGTCTTAAAATATTC-3′
pRS426-ZZ-3xF-Est2-Est1-Est3-SS	Est2 C623A/H435A	5’-TGAAGATAAGGCGTACATTAGAGAAGATGGTCTTTTTC -3′
		5′-ACCCACAAAGCTGTTTTAAATATTTC-3′
		5′-CAATTTCAATGCGAGCAAAATGAGGATTATACCAAAAAAAAG-3′
		5′-GACAACGTGTAACTATTATG-3′
pRS426-ZZ-3xF-Est2-Est1-Est3-SS	Est2 C422A/H435A	5′-AAACAACGTAGCGAGAAACCATAATAGTTACACG-3′
		5′-TCGACTAAGTACGTCTTAAAATATTC-3′
		5′-CAATTTCAATGCGAGCAAAATGAGGATTATACCAAAAAAAAG-3′
		5′-GACAACGTGTAACTATTATG-3′
pRS426-ZZ-3xF-Est2-Est1-Est3-SS	Est2 C422A/H425A/H435A/C623A	5’-TGAAGATAAGGCGTACATTAGAGAAGATGGTCTTTTTC -3′
		5′-ACCCACAAAGCTGTTTTAAATATTTC-3′
		5′-ACGTTGTCCAATTTCAATGCTAGCAAAATGAGGATTATACC-3′
		5′-GTAACTATTAGCGTTTCTAGCTACGTTGTTTTCGACTAAG-3′
pRS426-ZZ-3xF-Est2-Est1-Est3-SS	Est2 K62E/K111E	5′-CTTAAGCCACGAAGCAGTCATTG-3′
		5′-TCACCAGGAAGGCATG-3′
		5′-TACGTTACTGGAAGGCGCTGCTT-3′
		5′-ACGTTAACATTTGCAGAATGGC-3′
pRS426-ZZ-3xF-Est2-Est1-Est3-SS	Est2 F558A/H598A/S600A	5′-AAATGGGTTTGCGGTTAGATCTCAATATTTC-3′
		5′-TCATTTTTTAGCGCATCC-3′
		5′-TAGCAAATCAGGATGTTATAAACGTTG-3′
		5′-AAGCAACCGTCCTCACATTATC-3′
pRS426-ZZ-3xF-Est2-Est1-Est3-SS	Est3 E114A/I165G/D166A	5′-GCGACGACCAATTGCTTAATGATCATTGGCGATGCT-3′
		5′-TAAGCAATTGGTCGTCGCAGATGTGATCCTGCAATT-3′
		5′-GGTGCTCAAGTCGGATCGTTAAGTACTTTCCCATTT-3′
		5′-CGATCCGACTTGAGCACCATCAAATATCGTGGCCTG-3′
pRS426-ZZ-3xF-Est2-Est1-Est3-SS	Est3 E114A/I165G/D166A/H54A/M55G	5′-GCGACGACCAATTGCTTAATGATCATTGGCGATGCT-3′

		5′-TAAGCAATTGGTCGTCGCAGATGTGATCCTGCAATT-3′
		5′-GGTGCTCAAGTCGGATCGTTAAGTACTTTCCCATTT-3′
		5′-CGATCCGACTTGAGCACCATCAAATATCGTGGCCTG-3′
		5′-GCTGGGAGCCCGACAATTTTAACCAATCCGTGCCAT-3′
		5′-AATTGTCGGGCTCCCAGCCGGTAGCGCTAAGTCCTG-3′
pRS426-ZZ-3xF-Est2-Est1-Est3-SS	Est3 ΔNOB (Est3Δ2-19)	5′-CCATGGATAAAGGCATTAATTG-3′
		5′-CATGGTTTATTTATGTGTGTTTATTC-3′

C. Oligonucleotides used for purification and Southern blot

Names	Sequences	Note	Purpose
Yeast telomerase antisense template oligonucleotide	5′-biotin-CTAGACCTGTCATTTGUGUGUGGGUGUG-3′	Nucleotides without underlining are DNA, and underlined nucleotides are 2′-O-methyl RNA	Yeast telomerase purification
Yeast telomerase displacement oligo	5′-CACACCCACACACAAATGACAGGTCTAGddC-3′	ddC stands for 3′-dideoxycytidine	Yeast telomerase purification
Yeast telomerase substrate DNA	5′-TAGTAGTGTGTGGTGTGTGGG-3′	-	Yeast telomerase activity assay
pCT300_pVZ1_F	5′-TGCCTGCAGGTCGACTCTAG-3′	-	Primers to amplify pCT300 probe for Southern blots
pCT300_pVZ1_R	5′-AAACGACGGCCAGTGAATTG-3′	-	
CENIV_probe_F	5′-ATGCTGTCTCACCATAGAGAAT-3′	-	Primers to amplify CENIV probe from genomic DNA, also for Southern blots
CENIV_probe_R	5′-CGCTCCTAGGTAGTGCTTT-3′	-	

Movie S1.

3D variability analysis of the full yeast telomerase complex performed in CryoSPARC.

References

112. H. Ehara *et al.*, Structure of the complete elongation complex of RNA polymerase II with basal factors. *Science* **357**, 921–924 (2017).

113. J. L. Stamos, A. M. Lentzsch, A. M. Lambowitz, Structure of a Thermostable Group II Intron Reverse Transcriptase with Template-Primer and Its Functional and Evolutionary Implications. *Molecular Cell* **68**, 926–939.e924 (2017).

114. D. A. Opulente *et al.*, Genomic and ecological factors shaping specialism and generalism across an entire subphylum. *bioRxiv*, (2023).

115. X. Yang *et al.*, Structural basis for protein–protein interactions in the 14-3-3 protein family. *Proceedings of the National Academy of Sciences* **103**, 17237–17242 (2006).

116. Y. He *et al.*, Structure of Tetrahymena telomerase-bound CST with polymerase α-primase. *Nature* **608**, 813–818 (2022).

117. M. Lisby, J. H. Barlow, R. C. Burgess, R. Rothstein, Choreography of the DNA Damage Response: Spatiotemporal Relationships among Checkpoint and Repair Proteins. *Cell* **118**, 699–713 (2004).

118. R. S. Sikorski, P. Hieter, A system of shuttle vectors and yeast host strains designed for efficient manipulation of DNA in Saccharomyces cerevisiae. *Genetics* **122**, 19–27 (1989).
